# Immune-related genetic enrichment in frontotemporal dementia: An analysis of genome-wide association studies

**DOI:** 10.1371/journal.pmed.1002487

**Published:** 2018-01-09

**Authors:** Iris Broce, Celeste M. Karch, Natalie Wen, Chun C. Fan, Yunpeng Wang, Chin Hong Tan, Naomi Kouri, Owen A. Ross, Günter U. Höglinger, Ulrich Muller, John Hardy, International FTD-Genomics Consortium, Parastoo Momeni, Christopher P. Hess, William P. Dillon, Zachary A. Miller, Luke W. Bonham, Gil D. Rabinovici, Howard J. Rosen, Gerard D. Schellenberg, Andre Franke, Tom H. Karlsen, Jan H. Veldink, Raffaele Ferrari, Jennifer S. Yokoyama, Bruce L. Miller, Ole A. Andreassen, Anders M. Dale, Rahul S. Desikan, Leo P. Sugrue

**Affiliations:** 1 Neuroradiology Section, Department of Radiology and Biomedical Imaging, University of California, San Francisco, San Francisco, California, United States of America; 2 Department of Psychiatry, Washington University, St. Louis, Missouri, United States of America; 3 Department of Cognitive Sciences, University of California, San Diego, La Jolla, California, United States of America; 4 Norwegian Centre for Mental Disorders Research (NORMENT), Institute of Clinical Medicine, University of Oslo, Oslo, Norway; 5 Division of Mental Health and Addiction, Oslo University Hospital, Oslo, Norway; 6 Department of Neuroscience, Mayo Clinic, Jacksonville, Florida, United States of America; 7 Department of Neurology, Technical University of Munich, Munich, Germany; 8 German Center for Neurodegenerative Diseases (DZNE), Munich, Germany; 9 Munich Cluster for Systems Neurology (SyNergy), Munich, Germany; 10 Institut for Humangenetik, Justus-Liebig-Universität, Giessen, Germany; 11 Department of Molecular Neuroscience, Institute of Neurology, University College London, London, United Kingdom; 12 Laboratory of Neurogenetics, Department of Internal Medicine, Texas Tech University Health Sciences Center, Lubbock, Texas, United States of America; 13 Department of Neurology, University of California, San Francisco, San Francisco, California, United States of America; 14 Department of Pathology and Laboratory Medicine, Perelman School of Medicine, University of Pennsylvania, Philadelphia, Pennsylvania, United States of America; 15 Institute of Clinical Molecular Biology, Christian-Albrechts-Universität zu Kiel, Kiel, Germany; 16 Norwegian PSC Research Center, Research Institute of Internal Medicine, Division of Cancer Medicine, Surgery and Transplantation, Oslo University Hospital, Rikshospitalet, Oslo, Norway; 17 Division of Gastroenterology, Institute of Medicine, University of Bergen, Bergen, Norway; 18 K.G. Jebsen Inflammation Research Centre, Research Institute of Internal Medicine, Division of Cancer Medicine, Surgery and Transplantation, Oslo University Hospital, Rikshospitalet, Oslo, Norway; 19 Department of Neurology, Brain Center Rudolf Magnus, University Medical Center Utrecht, Utrecht, the Netherlands; 20 Department of Radiology, University of California, San Diego, La Jolla, California, United States of America; 21 Department of Neurosciences, University of California, San Diego, La Jolla, California, United States of America; University of Cambridge, UNITED KINGDOM

## Abstract

**Background:**

Converging evidence suggests that immune-mediated dysfunction plays an important role in the pathogenesis of frontotemporal dementia (FTD). Although genetic studies have shown that immune-associated loci are associated with increased FTD risk, a systematic investigation of genetic overlap between immune-mediated diseases and the spectrum of FTD-related disorders has not been performed.

**Methods and findings:**

Using large genome-wide association studies (GWASs) (total *n* = 192,886 cases and controls) and recently developed tools to quantify genetic overlap/pleiotropy, we systematically identified single nucleotide polymorphisms (SNPs) *jointly* associated with FTD-related disorders—namely, FTD, corticobasal degeneration (CBD), progressive supranuclear palsy (PSP), and amyotrophic lateral sclerosis (ALS)—and 1 or more immune-mediated diseases including Crohn disease, ulcerative colitis (UC), rheumatoid arthritis (RA), type 1 diabetes (T1D), celiac disease (CeD), and psoriasis. We found up to 270-fold genetic enrichment between FTD and RA, up to 160-fold genetic enrichment between FTD and UC, up to 180-fold genetic enrichment between FTD and T1D, and up to 175-fold genetic enrichment between FTD and CeD. In contrast, for CBD and PSP, only 1 of the 6 immune-mediated diseases produced genetic enrichment comparable to that seen for FTD, with up to 150-fold genetic enrichment between CBD and CeD and up to 180-fold enrichment between PSP and RA. Further, we found minimal enrichment between ALS and the immune-mediated diseases tested, with the highest levels of enrichment between ALS and RA (up to 20-fold). For FTD, at a conjunction false discovery rate < 0.05 and after excluding SNPs in linkage disequilibrium, we found that 8 of the 15 identified loci mapped to the *human leukocyte antigen* (*HLA)* region on Chromosome (Chr) 6. We also found novel candidate FTD susceptibility loci within *LRRK2 (leucine rich repeat kinase 2)*, *TBKBP1 (TBK1 binding protein 1)*, and *PGBD5 (piggyBac transposable element derived 5)*. Functionally, we found that the expression of FTD–immune pleiotropic genes (particularly within the *HLA* region) is altered in postmortem brain tissue from patients with FTD and is enriched in microglia/macrophages compared to other central nervous system cell types. The main study limitation is that the results represent only clinically diagnosed individuals. Also, given the complex interconnectedness of the *HLA* region, we were not able to define the specific gene or genes on Chr 6 responsible for our pleiotropic signal.

**Conclusions:**

We show immune-mediated genetic enrichment specifically in FTD, particularly within the *HLA* region. Our genetic results suggest that for a subset of patients, immune dysfunction may contribute to FTD risk. These findings have potential implications for clinical trials targeting immune dysfunction in patients with FTD.

## Abstract 2

Rahul Desikan and colleagues use summary data from genome-wide association studies to investigate genetic overlap between frontotemporal dementia and a several immune-mediated diseases, and identify microglia and inflammation-associated genes that may play a role in FTD pathogenesis.

## Author summary

**Why was this study done?:**

- Frontotemporal dementia (FTD) is the leading cause of dementia in individuals less than 65 years old.
- Currently, there is no approved treatment of FTD and no diagnostic tests for predicting disease onset or measuring progression.
- Increasing evidence suggests that inflammation and immune system dysfunction play an important role in the pathogenesis of FTD.

**What did the researchers do and find?:**

- We used summary data from genome-wide association studies to investigate genetic overlap, or “pleiotropy,” between FTD and a variety of immune-mediated diseases.
- Through this approach, we found extensive FTD–immune genetic overlap within the *HLA* region on Chromosome 6, an area rich in genes related to microglial function, as well as in 3 genes not previously identified as contributing to the pathophysiology of FTD.
- Pointing to the functional relevance of these genetic results, we found that these candidate FTD–immune genes are differentially expressed in postmortem brains from patients with FTD compared to controls, and in microglia/macrophages compared with other central nervous system cells.
- Using bioinformatics tools, we explored protein and genetic interactions among our candidate FTD–immune genes. These results suggest that rather than a few individual loci, large portions of the *HLA* region may be associated with increased FTD risk.

**What do these findings mean?:**

- Immune dysfunction may play a role in the pathophysiology of a subset of FTD cases.
- For a subset of patients in whom immune dysfunction in general—and microglial activation in particular—is central to disease pathophysiology, anti-inflammatory treatment is an important area for further investigation.

## Introduction

Frontotemporal dementia (FTD) is a fatal neurodegenerative disorder and the leading cause of dementia among individuals younger than 65 years of age [1]. Given rapid disease progression and the absence of disease-modifying therapies, there is an urgent need to better understand FTD pathobiology to accelerate development of novel preventive and therapeutic strategies.

Converging molecular, cellular, genetic, and clinical evidence suggests that neuroinflammation plays a role in FTD pathogenesis. Complement factors and activated microglia, key components of inflammation, have been established as histopathologic features in brains of patients [2] and in mouse models of FTD [3,4]. Genome-wide association studies (GWASs) have shown that single nucleotide polymorphisms (SNPs) within the immune-regulating *human leukocyte antigen* (*HLA)* region on Chromosome (Chr) 6 are associated with elevated FTD risk [5]. Importantly, there is increased prevalence of immune-mediated diseases among patients with FTD [6,7]. Together, these findings suggest that immune-related mechanisms may contribute to and potentially drive FTD pathology.

Recent work in human molecular genetics has emphasized “pleiotropy,” where variations in a single gene can affect multiple, seemingly unrelated phenotypes [8]. In the present study, we systematically evaluated genetic pleiotropy between FTD and immune-mediated diseases. Using large neurodegenerative GWASs and recently developed tools to estimate polygenic pleiotropy, we sought to identify SNPs *jointly* associated with FTD-related disorders [9,10]—namely, FTD, corticobasal degeneration (CBD), progressive supranuclear palsy (PSP), and amyotrophic lateral sclerosis (ALS)—and 1 or more immune-mediated diseases including Crohn disease (CD), ulcerative colitis (UC), rheumatoid arthritis (RA), type 1 diabetes (T1D), celiac disease (CeD), and psoriasis (PSOR).

## Methods

### Participant samples

We conducted a meta-analysis of summary data obtained from published data. More specifically, we evaluated complete GWAS results in the form of summary statistics (*p*-values and odds ratios) for FTD, CBD, PSP, and ALS and 6 immune-mediated diseases, including CD [11], UC [12], RA [13], T1D [14], CeD [15], and PSOR [16] (see Table 1). We obtained FTD GWAS summary statistic data from phase I of the International FTD-Genomics Consortium (IFGC), which consisted of 2,154 clinical FTD cases and 4,308 controls with genotyped and imputed data at 6,026,384 SNPs (Table 1; for additional details, see [5]). The FTD dataset included multiple clinically diagnosed FTD subtypes: behavioral variant (bvFTD), semantic dementia (sdFTD), primary nonfluent progressive aphasia (pnfaFTD), and FTD overlapping with motor neuron disease (mndFTD). These FTD cases and controls were recruited from 44 international research groups and diagnosed according to the Neary criteria [17]. The institutional review boards of all participating institutions approved the procedures for all IFGC sub-studies. Written informed consent was obtained from all participants or surrogates. We obtained CBD GWAS summary statistic data from 152 CBD cases and 3,311 controls at 533,898 SNPs (Table 1; for additional details, see [18]). The CBD cases were collected from 8 institutions, and controls were recruited from the Children’s Hospital of Philadelphia. CBD was neuropathologically diagnosed using the National Institutes of Health Office of Rare Diseases Research criteria [19]. The institutional review boards of all participating institutions approved the procedures for CBD GWAS data. Written informed consent was obtained from all participants or surrogates. We obtained PSP GWAS summary statistic data (stage 1) from the National Institute on Aging Genetics of Alzheimer’s Disease Data Storage Site (NIAGADS, https://www.niagads.org) for 1,114 individuals with autopsy-confirmed PSP and 3,247 controls at 531,451 SNPs (Table 1; for additional details, see [20]). The institutional review boards of all participating institutions approved the procedures for all NIAGADS sub-studies. Written informed consent was obtained from all participants or surrogates. We obtained ALS GWAS summary statistic data from 12,577 ALS cases and 23,475 controls at 18,741,501 SNPs (Table 1; for additional details, see [21]). The ALS GWAS summary statistics and sequenced variants are publicly available through the Project MinE Data Browser (http://databrowser.projectmine.com). The institutional review boards of all participating institutions approved the procedures for all ALS GWAS sub-studies. Written informed consent was obtained from all participants or surrogates.

**Table 1:** Summary data from all genome-wide association studies used in the current study.

Disorder/disease	Abbreviation	Total *N*	Number of SNPs	Reference
Frontotemporal dementia	FTD	6,462	6,026,384	[5]
Corticobasal degeneration	CBD	3,463	533,898	[18]
Progressive supranuclear palsy	PSP	4,361	531,451	[20]
Amyotrophic lateral sclerosis	ALS	36,052	18,741,501	[21]
Crohn disease	CD	51,109	942,858	[11]
Ulcerative colitis	UC	26,405	1,273,589	[12]
Rheumatoid arthritis	RA	25,708	2,554,714	[13]
Type 1 diabetes	T1D	16,559	841,622	[14]
Celiac disease	CeD	15,283	528,969	[15]
Psoriasis	PSOR	7,484	1,121,166	[16]

### Genetic enrichment statistical analyses

The pleiotropic enrichment strategies implemented here were derived from previously published stratified false discovery rate (FDR) methods [22,23]. For given phenotypes A and B, pleiotropic “enrichment” between phenotype A and phenotype B exists if the proportion of SNPs or genes associated with phenotype A increases as a function of increased association with phenotype B. To assess for enrichment, we constructed fold enrichment plots of nominal −log10(*p*)-values for all FTD-related-disorder SNPs and for subsets of SNPs determined by the significance of their association with the 6 immune-mediated diseases. In fold enrichment plots, the presence of enrichment is reflected as an upward deflection of the curve for phenotype A with increasing strength of association with phenotype B. To assess for polygenic effects below the standard GWAS significance threshold, we focused the fold enrichment plots on SNPs with nominal −log10(*p*) < 7.3 (corresponding to *p* > 5 × 10^−8^). The enrichment seen can be directly interpreted in terms of the true discovery rate (1 − FDR).

To identify specific loci jointly involved with each of the 4 FTD-related disorders and the 6 immune-mediated diseases, we computed conjunction FDRs. The conjunction FDR is a test of association between 2 traits [22]. Briefly, the conjunction FDR, denoted by FDR_trait1& trait2_, is defined as the posterior probability that a SNP is null for either trait or for both simultaneously, given that the *p*-values for both traits are as small, or smaller, than the observed *p*-values. Unlike the conditional FDR, which ranks disease/primary-phenotype-associated SNPs based on genetic “relatedness” with secondary phenotypes [24], the conjunction FDR minimizes the possibility/likelihood of a single phenotype driving the common association signal. The conjunction FDR therefore tends to be more conservative and specifically pinpoints pleiotropic loci shared between the traits/diseases of interest. We used an overall FDR threshold of <0.05, which means 5 expected false discoveries per 100 reported. To visualize the results of our conjunction FDR analysis, we constructed Manhattan plots to illustrate the genomic location of the pleiotropic loci. We ranked all SNPs based on the conjunction FDR and removed SNPs in linkage disequilibrium (*r*^2^ > 0.2) with any higher ranked SNP. Key aspects and detailed information on fold enrichment plots, Manhattan plots, and conjunction FDRs can be found in prior reports [22,23,25,26].

### Functional evaluation of shared risk loci

To assess whether SNPs that are shared between FTD and immune-mediated disease modify gene expression, we identified *cis*-expression quantitative trait loci (*cis*-eQTLs, defined as variants within 1 Mb of a gene’s transcription start site) associated with shared FTD–immune SNPs and measured their regional brain expression in a publicly available dataset of normal control brains (UK Brain Expression Consortium; http://braineac.org/) [27]. We also evaluated *cis*-eQTLs using a blood-based dataset [28]. We applied an analysis of covariance (ANCOVA) to test for associations between genotypes and gene expression. We tested SNPs using an additive model.

### Network-based functional association analyses

To evaluate potential protein and genetic interactions, co-expression, co-localization, and protein domain similarity for the functionally expressed (i.e., with significant *cis*-eQTLs) pleiotropic genes, we used GeneMANIA (http://genemania.org), an online web portal for bioinformatic assessment of gene networks [29]. In addition to visualizing the composite gene network, we also assessed the weights of individual components within the network [30].

### Gene expression alterations in FTD brains

To determine whether functionally expressed (i.e., with significant *cis*-eQTLs) pleiotropic genes are differentially expressed in the brains of FTD patients, we analyzed the gene expression of pleiotropic genes. Postmortem expression data from the brains of 17 patients with frontotemporal lobar degeneration with ubiquitinated inclusions (FTD-U) (with and without progranulin *[GRN]* mutations) and 11 controls were obtained from a publically available dataset (Gene Expression Omnibus [GEO] dataset GSE13162; for additional details, see [31]). These data consist of global gene expression profiles from all histopathologically available regions from human FTD-U and control brains (frontal cortex, hippocampus, and cerebellum) analyzed on the Affymetrix U133A microarray platform. Given the small sample size of each individual region, we combined all 3 regions to maximize statistical power. Details about this dataset and analysis—including the human brain samples used, RNA extraction and hybridization methods, microarray quality control, and microarray data analysis—are provided in the original report [31].

### Evaluation of cell classes within the brain

Using a publicly available RNA-sequencing transcriptome and splicing database [32], we ascertained whether the functionally expressed (i.e., with significant *cis*-eQTLs) pleiotropic genes were expressed by specific cell classes within the brain. The 8 cell types surveyed were neurons, fetal and mature astrocytes, oligodendrocyte precursor cells, newly formed oligodendrocytes, myelinating oligodendrocytes, microglia/macrophages (henceforth “microglia”), endothelial cells, and pericytes (for additional details, see [32]).

## Results

### Shared genetic risk between FTD and immune-mediated disease

**Fig 1:**
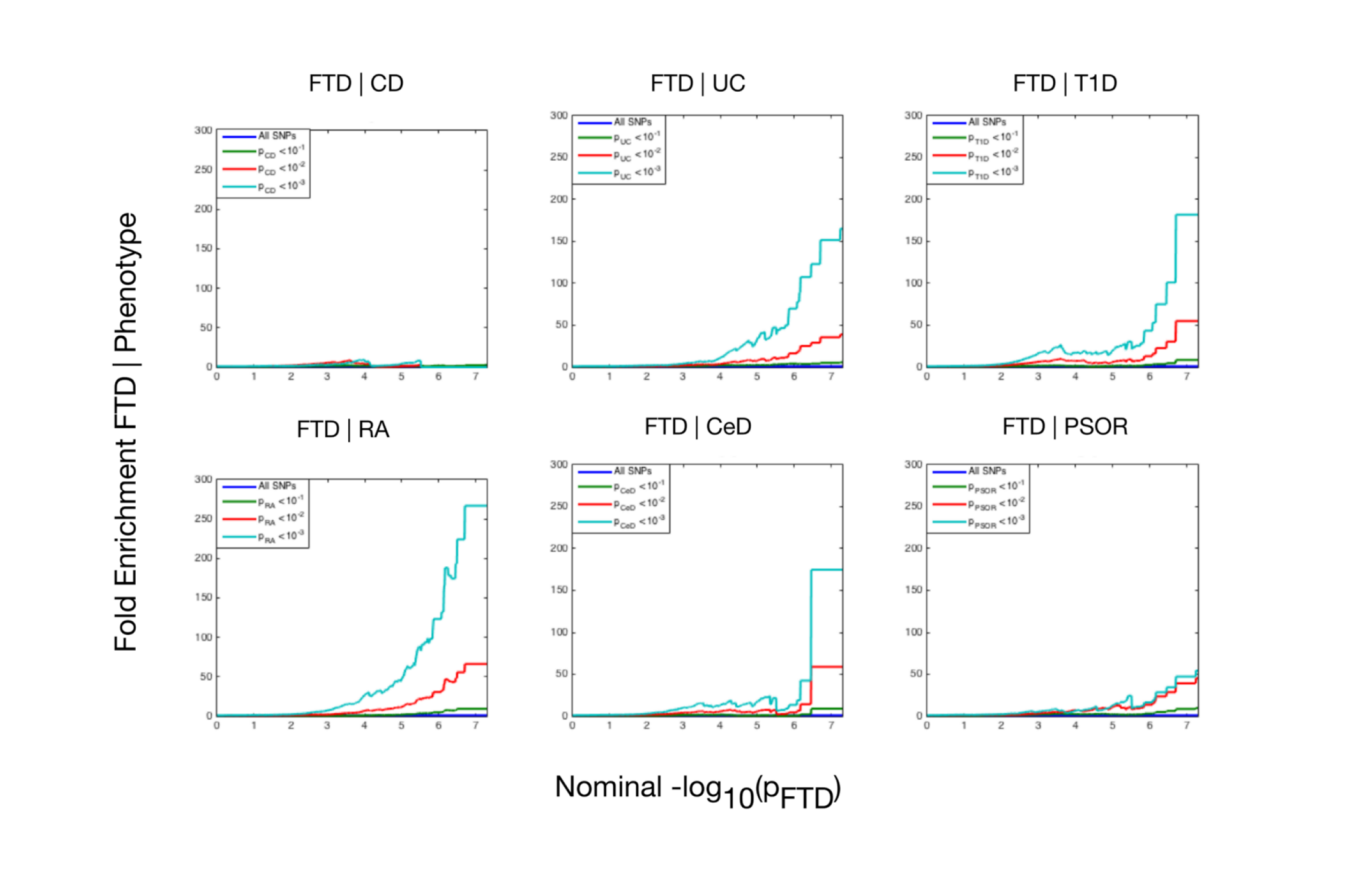
Fold enrichment plots of enrichment versus nominal −log_10_(*p*)-values (corrected for inflation) in frontotemporal dementia (FTD) below the standard genome-wide association study threshold of *p* < 5 × 10^−8^ as a function of significance of association with 6 immune-mediated diseases. The 6 immune-mediated diseases are Crohn disease (CD), ulcerative colitis (UC), type 1 diabetes (T1D), rheumatoid arthritis (RA), celiac disease (CeD), and psoriasis (PSOR). The levels of −log_10_(*p*) > 0, −log_10_(*p*) > 1, and −log_10_(*p*) > 2 correspond to *p* < 1, *p* < 0.1, and *p* < 0.01, respectively. The dark blue line indicates all SNPs.

Using progressively stringent *p*-value thresholds for FTD SNPs (i.e., increasing values of nominal −log_10_[*p*]), we observed genetic enrichment for FTD as a function of several immune-mediated diseases (Fig 1). More specifically, we found strong (up to 270-fold) genetic enrichment between FTD and RA, and comparable enrichment between FTD and UC, T1D, and CeD, with weaker enrichment between FTD and PSOR and CD.

**Fig 2:**
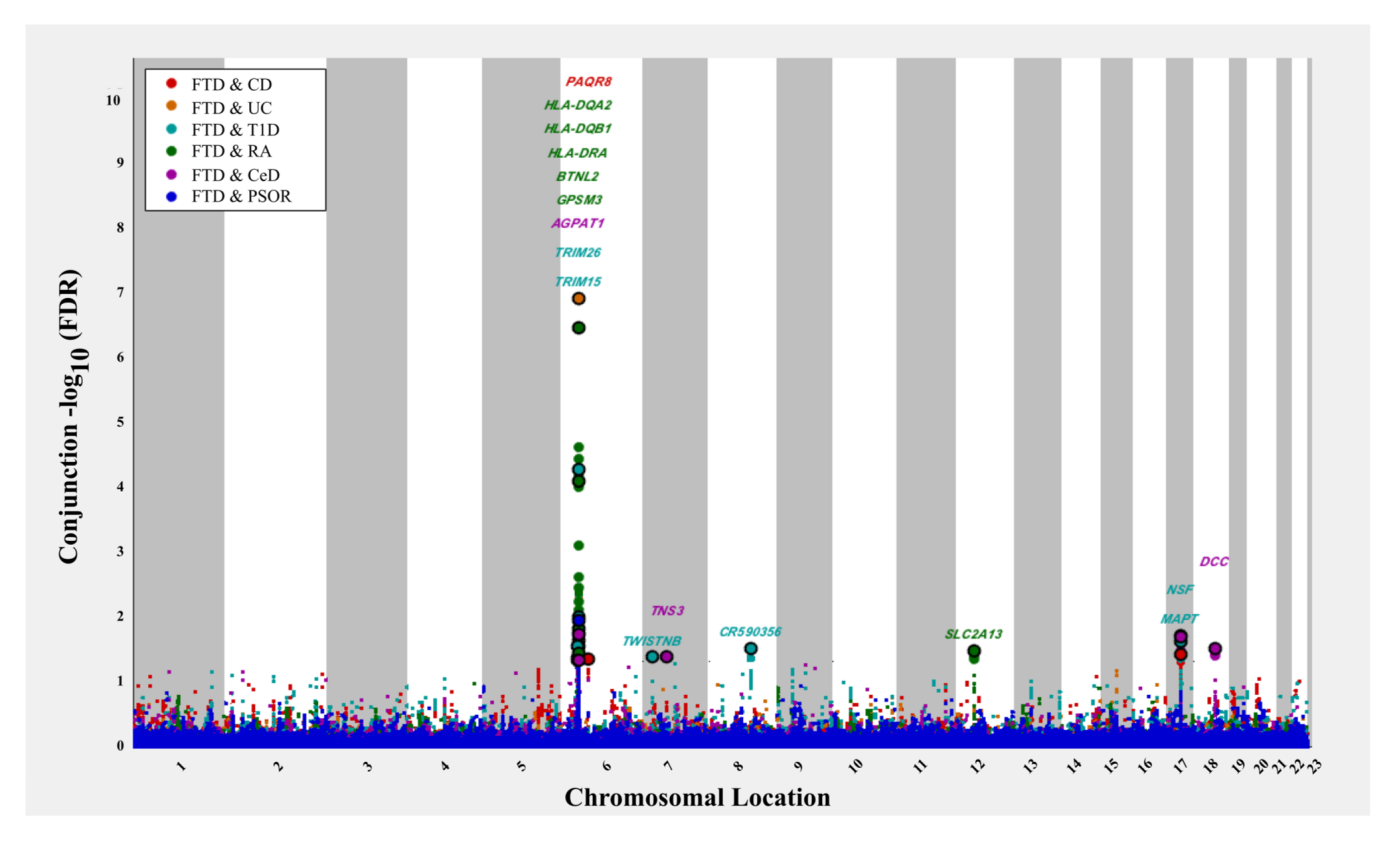
“Conjunction” Manhattan plot of conjunction −log_10_(FDR) values for frontotemporal dementia (FTD) given 6 immune-mediated diseases. The 6 immune-related diseases were Crohn disease (CD; FTD|CD, red), ulcerative colitis (UC, FTD|UC, orange), type 1 diabetes (T1D, FTD|T1D, teal), rheumatoid arthritis (RA, FTD|RA, green), celiac disease (CeD, FTD|CeD, magenta), and psoriasis (PSOR, FTD|PSOR, blue). SNPs with conjunction −log_10_(FDR) > 1.3 (i.e., FDR < 0.05) are shown with large points. A black line around the large points indicates the most significant SNP in each linkage disequilibrium block, and this SNP was annotated with the closest gene, which is listed above the symbols in each locus.

**Fig 3:**
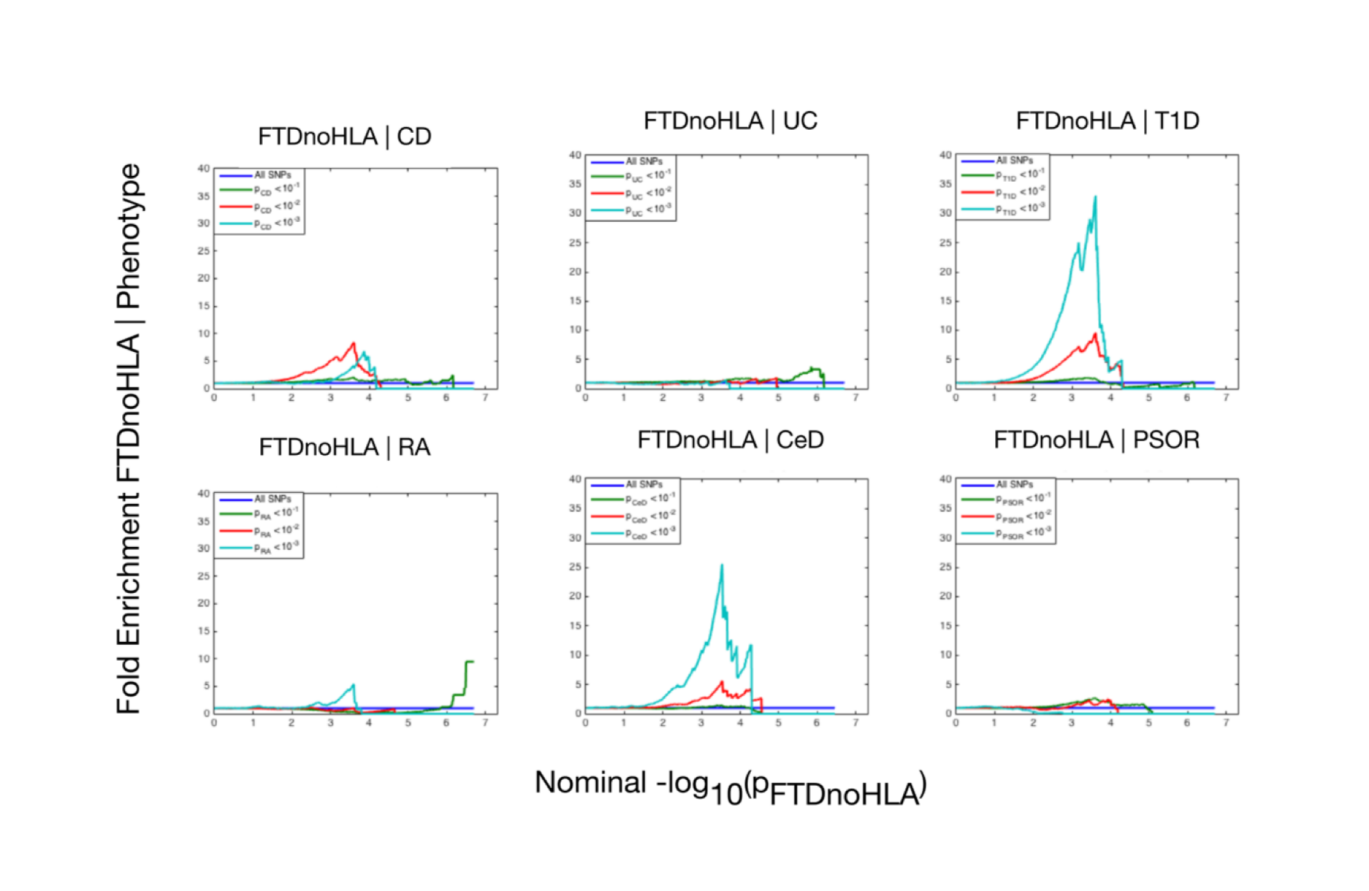
Fold enrichment plots of enrichment (after removing all regions in linkage disequilibrium with *HLA* on Chromosome 6) versus nominal −log_10_(*p*)-values (corrected for inflation) in frontotemporal dementia (FTD) below the standard genome-wide association study threshold of *p* < 5 × 10^−8^ as a function of significance of association with 6 immune-mediated diseases. The 6 immune-mediated diseases were Crohn disease (CD), ulcerative colitis (UC), type 1 diabetes (T1D), rheumatoid arthritis (RA), celiac disease (CeD), and psoriasis (PSOR). The levels of −log_10_(*p*) > 0, −log_10_(*p*) > 1, and −log_10_(*p*) > 2 correspond to *p* < 1, *p* < 0.1, and *p* < 0.01, respectively. The dark blue line indicates all SNPs.

At a conjunction FDR *<* 0.05, we identified 21 SNPs that were associated with both FTD and immune-mediated diseases (Fig 2; Table 2). Five of these SNPs demonstrated the opposite direction of allelic effect between FTD and the immune-mediated diseases (Table 2): (1) rs9261536, nearest gene = *TRIM15*; (2) rs3094138, nearest gene = *TRIM26*; (3) rs9268877, nearest gene = *HLA-DRA*; (4) rs10484561, nearest gene = *HLA-DQB1*; and (5) rs2269423, nearest gene = *AGPAT1*. Of the remaining 16, 2 SNPs showed strong linkage disequilibrium (LD), suggesting that they reflected the same signal: rs204991 and rs204989 (nearest gene: *GPSM3*; pairwise *D*′ = 1, *r*^2^ = 1). After excluding SNPs that demonstrated the opposite direction of allelic effect and SNPs that were in LD, we found that 8 of the remaining 15 identified loci mapped to the *HLA* region, suggesting that *HLA* markers were critical in driving our results. To test this hypothesis, we repeated our enrichment analysis after removing all SNPs in LD with *r*^2^ > 0.2 within 1 Mb of *HLA* variants (based on 1000 Genomes Project LD structure). After removing *HLA* SNPs, we saw considerable attenuation of genetic enrichment in FTD as a function of immune-mediated disease (Fig 3), suggesting that the observed overlap between immune-related diseases and FTD was largely driven by the *HLA* region. Further, to determine causal associations for FTD and the 6 immune-mediated diseases, we applied the recently developed summary-data-based Mendelian randomization (SMR; http://cnsgenomics.com/software/smr/) method. This approach is described in detail within the original report [33]. As shown in S1 Table, results from the SMR analysis have identified significant loci that are consistent with the main findings, which suggest that *HLA* markers on Chr 6 are critical in driving our pleiotropic results.

**Table 2:** Overlapping loci between FTD and immune-mediated disease at a conjunction FDR < 0.05.

SNP	Chr	Nearest gene	Reference immune disease	Reference immune disease *p*-value	Minimum conjunction FDR	FTD *p*-value	Direction of allelic effect
rs2269423	6	*AGPAT1*	CeD	4.63 × 10^−2^	4.63 × 10^−2^	6.28 × 10^−1^	+/−
rs3117097	6	*BTNL2*	RA	8.21 × 10^−5^	8.21 × 10^−5^	7.19 × 10^−3^	+/+
rs204989	6	*GPSM3*	UC	2.90 × 10^−2^	9.02 × 10^−3^	2.58 × 10^−1^	+/+
rs204991	6	*GPSM3*	T1D	1.00 × 10^−2^	9.02 × 10^−3^	2.58 × 10^−1^	+/+
rs17427887	6	*HLA-DQA2*	RA	3.70 × 10^−2^	3.70 × 10^−2^	6.02 × 10^−1^	−/−
rs10484561	6	*HLA-DQB1*	T1D	1.86 × 10^−2^	1.71 × 10^−2^	4.17 × 10^−1^	−/+
rs3135353	6	*HLA-DRA*	CD	2.29 × 10^−2^	3.58 × 10^−3^	1.35 × 10^−1^	+/+
rs9268852	6	*HLA-DRA*	UC	1.25 × 10^−7^	1.25 × 10^−7^	1.03 × 10^−4^	+/+
rs3129890	6	*HLA-DRA*	T1D	5.54 × 10^−5^	5.54 × 10^−5^	7.19 × 10^−3^	+/+
rs6457590	6	*HLA-DRA*	RA	1.52 × 10^−2^	1.52 × 10^−2^	3.80 × 10^−1^	+/+
rs9268877	6	*HLA-DRA*	RA	3.64 × 10^−7^	1.25 × 10^−7^	1.03 × 10^−4^	+/−
rs875142	6	*PAQR8*	CD	4.62 × 10^−2^	4.62 × 10^−2^	5.51 × 10^−1^	−/−
rs9261536	6	*TRIM15*	T1D	4.31 × 10^−2^	4.31 × 10^−2^	6.98 × 10^−1^	−/+
rs3094138	6	*TRIM26*	T1D	4.63 × 10^−2^	2.87 × 10^−2^	6.28 × 10^−1^	−/+
rs7778450	7	*TNS3*	CeD	4.26 × 10^−2^	4.26 × 10^−2^	6.17 × 10^−1^	−/−
rs2192493	7	*TWISTNB*	T1D	4.17 × 10^−2^	4.17 × 10^−2^	6.09 × 10^−1^	+/+
rs10216900	8	*CR590356*	T1D	3.09 × 10^−2^	3.09 × 10^−2^	6.40 × 10^−1^	+/+
rs10784359	12	*SLC2A13*	RA	3.33 × 10^−2^	3.33 × 10^−2^	5.90 × 10^−1^	+/+
rs17572851	17	*MAPT*	T1D	2.47 × 10^−2^	2.47 × 10^−2^	5.90 × 10^−1^	+/+
rs199533	17	*NSF*	CD	3.90 × 10^−2^	1.95 × 10^−2^	4.56 × 10^−1^	+/+
rs2134297	18	*DCC*	CeD	3.11 × 10^−2^	3.11 × 10^−2^	5.73 × 10^−1^	+/+

CD, Crohn disease; CeD, celiac disease; Chr, Chromosome; FDR, false discovery rate; FTD, frontotemporal dementia; RA, rheumatoid arthritis; SNP, single nucleotide polymorphism; T1D, type 1 diabetes; UC, ulcerative colitis; −, negative effect estimate; +, positive effect estimate.

Outside the *HLA* region, we found 7 other FTD- and immune-associated SNPs (Fig 2; Table 2), including 2 in strong LD that mapped to the H1 haplotype of *microtubule associated protein tau (MAPT)* (LD: rs199533 and rs17572851; nearest genes: *NSF* and *MAPT*, pairwise *D′* = 1, *r*^2^ = 0.94). Beyond *MAPT*, we found 5 additional novel loci associated with increased FTD risk, namely, (1) rs2192493 (Chr 7, nearest gene = *TWISTNB*), (2) rs7778450 (Chr 7, nearest gene = *TNS3*), (3) rs10216900 (Chr 8, nearest gene = *CR590356*), (4) rs10784359 (Chr 12, nearest gene = *SLC2A13*), and (5) rs2134297 (Chr 18, nearest gene = *DCC*) (see Table 2 for additional details).

### Modest genetic enrichment between immune-mediated disease and PSP, CBD, and ALS

To evaluate the specificity of the shared genetic overlap between FTD and immune-mediated disease, we also evaluated overlap between the 6 immune-mediated diseases and CBD, PSP, and ALS. For CBD and PSP, a few of the immune-mediated diseases produced genetic enrichment comparable to that seen for FTD (S1–S3 Figs; S2–S4 Tables). For example, we found 150-fold genetic enrichment between CBD and CeD and 180-fold enrichment between PSP and RA. In contrast, we found minimal enrichment between ALS and the immune-mediated diseases tested, with the highest levels of enrichment between ALS and RA (up to 20-fold) and between ALS and CeD (up to 15-fold).

At a conjunction FDR < 0.05, we identified several SNPs associated with both immune-mediated disease and CBD, PSP, or ALS (S4–S6 Figs; S2–S4 Tables). Few of the SNPs shared between CBD, PSP, or ALS and immune-mediated disease mapped to the *HLA* region. Only 2 PSP–immune SNPs mapped to the region of *MLN* and *IRF4* on Chr 6, and no CBD–immune or ALS–immune SNPs mapped to the *HLA* region (S4–S6 Figs; S2–S4 Tables).

Beyond the *HLA* region, we found several overlapping loci between the immune- mediated diseases and CBD, PSP, and ALS (S4–S6 Figs; S2–S4 Tables). For PSP, these were (1) rs7642229 with CeD (Chr 3, nearest gene = *XCR1*, FDR = 1.74 × 10^−2^); (2) rs11718668 with CeD (Chr 3, nearest gene = *TERC*, FDR = 3.00 × 10^−2^); (3) rs12203592 with CeD (Chr 6, nearest gene = *IRF4*, FDR = 4.17 × 10^−2^); (4) rs1122554 with RA (Chr 6, nearest gene = *MLN*, FDR = 2.09 × 10^−2^); and (5) rs3748256 with RA (Chr 11, nearest gene = *FAM76B*, FDR = 2.09 × 10^−2^). For ALS, these were (1) rs3828599 with CeD (Chr 5, nearest gene = *GPX3*, FDR = 2.27 × 10^−2^) and (2) rs10488631 with RA (Chr 7, nearest gene = *TNPO3*, FDR = 3.42 × 10^−2^).

### *cis*-eQTL expression

To investigate whether shared FTD–immune SNPs modify gene expression, we evaluated *cis-*eQTLs in both brain and blood tissue types. At a previously established conservative Bonferroni-corrected *p*-value < 3.9 × 10^−5^ [34], we found significant *cis*-associations between shared SNPs and genes in the *HLA* region on Chr 6 in peripheral blood mononuclear cells, lymphoblasts, and the human brain (see S5 Table for gene expression associated with each SNP). We also found that rs199533 and rs17572851 on Chr 17 were significantly associated with *MAPT* (*p* = 2 × 10^−12^) expression in the brain. Beyond the *HLA* and *MAPT* regions, notable *cis*-eQTLs included rs10784359 and *LRRK2* (*p* = 1.40 × 10^− 7^) and rs2192493 and *TBKBP1* (*p* = 1.29 × 10^−6^) (see S5 Table).

### Protein–protein and co-expression networks

**Fig 4:**
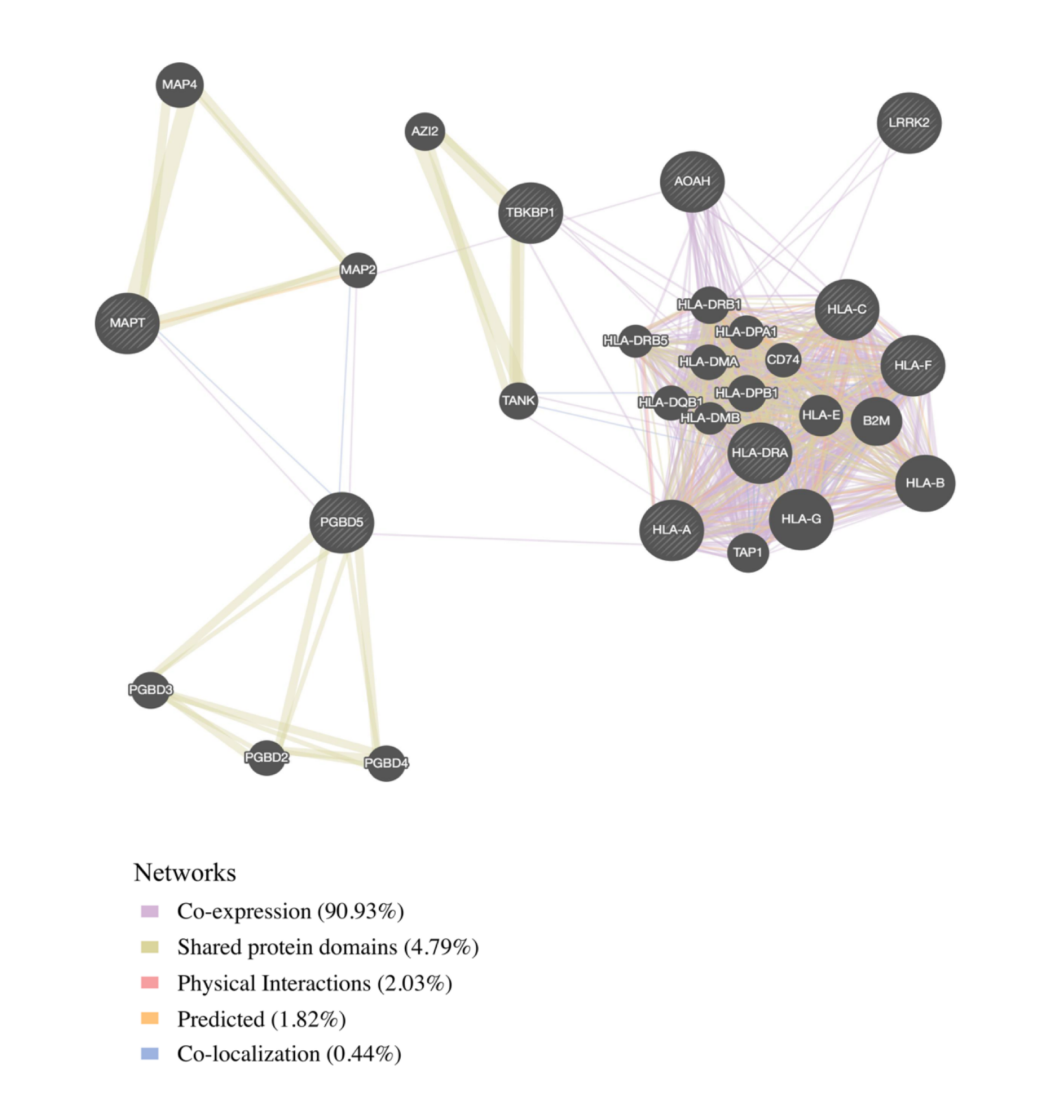
Network interaction graph predominantly illustrating co-expression and shared protein domains for functionally expressed pleiotropic genes between frontotemporal dementia (FTD) and immune-related diseases.

We found physical interaction and gene co-expression networks for the FTD–immune pleiotropic genes with significant *cis*-eQTLs (at a Bonferroni-corrected *p*-value < 3.9 × 10^−5^). We found robust co-expression between various *HLA* classes, further suggesting that large portions of the *HLA* region, rather than a few individual loci, may be involved with FTD (Fig 4; S6 Table). Interestingly, we found that several non-*HLA* functionally expressed FTD–immune genes, namely, *LRRK2*, *PGBD5*, and *TBKBP1*, showed co-expression with *HLA*-associated genes (Fig 4).

### Genetic expression in FTD brains compared to controls

**Fig 5:**
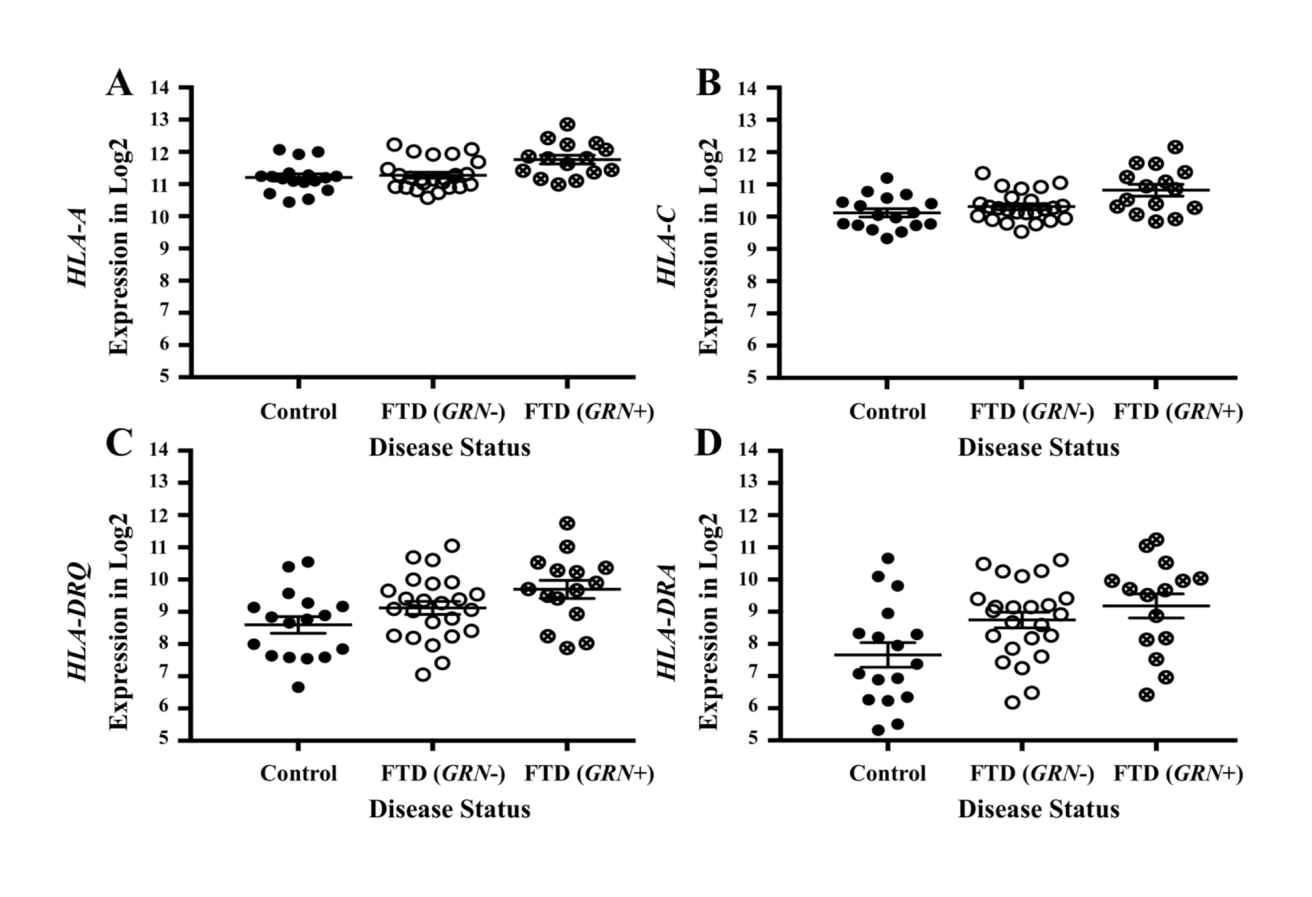
Pleiotropic genes between frontotemporal dementia (FTD) and immune-related diseases are elevated in brains of patients with FTD with *GRN* mutation. Expression for the genes with the largest effect sizes are plotted: (A) *HLA-A*, (B) *HLA-C*, (C) *HLA-DRQ*, and (D) *HLA-DRA*. Expression values were obtained from GSE13162 for FTD-U brains with and without *GRN* mutations and neuropathology-free controls. Horizontal bar represents mean ± SEM.

To investigate whether the FTD–immune pleiotropic genes with significant *cis*-eQTLs are differentially expressed in FTD brains, we compared gene expression in FTD-U brains to that in brains from neurologically healthy controls. We found significantly different levels of *HLA* gene expression in FTD-U brains compared to control brains (Table 3). This was true of FTD-U brains regardless of progranulin gene (*GRN*) mutation status. In spite of the fact that the FTD GWAS used to identify these genes was based on patients with sporadic FTD (without *GRN* mutations), *GRN* mutation carriers showed the greatest differences in *HLA* gene expression (Fig 5; Table 3). These findings are compatible with prior work showing microglial-mediated immune dysfunction in *GRN* mutation carriers [3].

**Table 3:** Genes associated with frontotemporal dementia (FTD) and immune-mediated disease differentially altered in patients with frontotemporal lobar degeneration with ubiquitinated inclusions (FTD-U) versus controls.

Gene	Control versus FTD-U *p*-value	Control versus *GRN*+ *p*-value
*AOAH*	0.631	**0.013**
*HLA-A*	0.622	**0.004**
*HLA-C*	0.372	**0.002**
*HLA-F*	0.592	**0.001**
*HLA-DRA (LOC101060835)*	**0.017**	**0.008**
*HLA-DRQ (HLA-DQB1)*	**0.009**	**0.001**
*MAPT*	0.376	0.090
*TBKBP1*	**0.005**	0.108
*PGBD5*	0.528	**0.016**
*LRRK2*	N/A	N/A

*p-*Values ≤ 0.05 are in bold.

N/A, not available.

### Microglial enrichment

**Fig 6:**
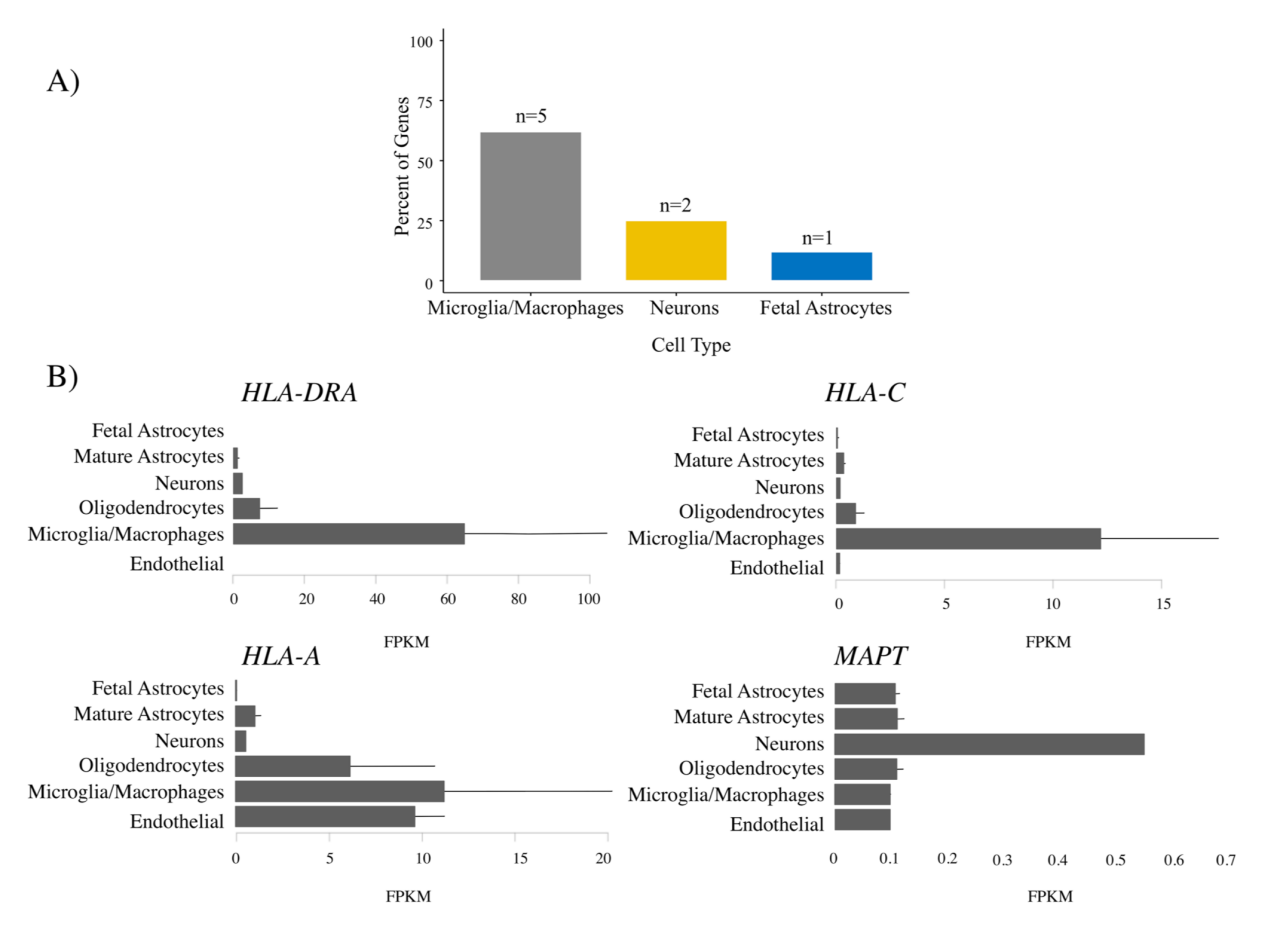
Microglia enrichment in genes associated with frontotemporal dementia (FTD) and immune-mediated disease. FTD–immune genes were analyzed to determine the cell type in which each gene was most highly expressed [32]. (A) Bar plots showing the relative number of genes most highly expressed in each cell type. No genes were most highly expressed in endothelial cells or oligodendrocytes. See Table 4 for individual gene names. (B) Individual bar plots showing cell-type-specific expression for genes with the largest effect size. Note that the horizontal scale is not the same in all the plots. FPKM, fragments per kilobase of transcript per million.

For the FTD–immune pleiotropic genes with significant *cis*-eQTLs, across different central nervous system (CNS) cell types, we found significantly greater expression within microglia compared to neurons or fetal astrocytes (Fig 6A; Table 4). Interestingly, *HLA* genes showed the greatest degree of differential expression. Four of the FTD–immune *HLA*-associated genes, namely *HLA-DRA*, *AOAH*, *HLA-A*, and *HLA-C*, showed highest expression in microglia (ranging from 10 to 60 fragments per kilobase of transcript per million; see Fig 6B). In addition, *MAPT* was predominantly expressed in neurons, *LRRK2* in microglia, *PGBD5* in neurons, and *TBKBP1* in fetal astrocytes (Figs 6B and S7–S9).

**Table 4:** Enrichment of genes associated with frontotemporal dementia (FTD) and immune-mediated disease in microglia compared with other central nervous system cells.

Gene	Most enriched cell type
*AOAH *	Microglia/macrophage
*HLA-A *	Microglia/macrophage
*HLA-C *	Microglia/macrophage
*HLA-F *	Neurons
*HLA-DRA *	Microglia/macrophage
*HLA-DRQ *	Not found
*LRRC37A3 *	Microglia/macrophage
*MAPT *	Neurons
*TRAM2*	Fetal astrocytes

No genes were most highly expressed in endothelial cells or oligodendrocytes.

## Discussion

We systematically assessed genetic overlap between 4 FTD-related disorders and several immune-mediated diseases. We found extensive genetic overlap between FTD and immune-mediated disease particularly within the *HLA* region on Chr 6, a region rich in genes associated with microglial function. This genetic enrichment was specific to FTD and did not extend to CBD, PSP, or ALS. Further, we found that shared FTD–immune gene variants were differentially expressed in FTD patients compared with controls, and in microglia compared with other CNS cells. Beyond the *HLA* region, by leveraging statistical power from large immune-mediated GWASs, we detected novel candidate FTD associations requiring validation within *LRRK2*, *TBKBP1*, and *PGBD5*. Considered together, these findings suggest that various microglia and inflammation-associated genes, particularly within the *HLA* region, may play a critical and selective role in FTD pathogenesis.

By combining GWASs from multiple studies and applying a pleiotropic approach, we identified genetic variants jointly associated with FTD-related disorders and immune-mediated disease. We found that the strength of genetic overlap with immune-mediated disease varies markedly across FTD-related disorders, with the strongest pleiotropic effects associated with FTD, followed by CBD and PSP, and the weakest pleiotropic effects associated with ALS. We identified 8 FTD- and immune-associated loci that mapped to the *HLA* region, a concentration of loci that was not observed for the other disorders. Indeed, only 2 PSP–immune pleiotropic SNPs and no CBD–immune or ALS–immune pleiotropic SNPs mapped to the *HLA* region. Previous work has identified particular *HLA* genes associated with CBD, PSP, and ALS [35,36]. In contrast, our current findings implicate large portions of the *HLA* region in the pathogenesis of FTD. Together, these results suggest that each disorder across the larger FTD spectrum has a unique relationship with the *HLA* region.

Our results also indicate that functionally expressed FTD–immune shared genetic variants are differentially expressed in FTD brains compared to controls and in microglia compared to other CNS cell types (Fig 6). Microglia play a role in the pathophysiology of *GRN*+ FTD. Progranulin is expressed in microglia [37], and *GRN* haploinsufficiency is associated with abnormal microglial activation and neurodegeneration [3]. It is perhaps expected, therefore, that *GRN+* brains show differential expression of FTD–immune genes, even though these genetic variants were derived from a GWAS of patients with sporadic FTD (who lack *GRN* or other established FTD mutations). More surprising is the presence of similar enrichment in *GRN*− brains, suggesting that dysfunction of microglial-centered immune networks may be a common feature of FTD pathogenesis.

By leveraging statistical power from the large immune-mediated GWASs, we identified novel candidate FTD associations requiring validation within *LRRK2*, *TBKBP1*, and *PGBD5* and confirmed previously shown FTD-associated signal within the *MAPT* region. *LRRK2* mutations are a cause of Parkinson disease [38] and CD [39]. We previously described a potential link between FTD and the *LRRK2* locus [40], and another study using a small sample showed that *LRRK2* mutations may increase FTD risk [41]. Together, these results suggest that the extended *LRRK2* locus might influence FTD despite common genetic variants within *LRRK2* not reaching genome-wide significance in a large FTD GWAS [5]. We suggest that increased expression of *LRRK2* in microglia results in proinflammatory responses, possibly by modulating TNF-alpha secretion [42]. *TBKBP1* also modulates TNF-alpha signaling by binding to *TBK1 (TANK binding kinase 1)* [43]; rare pathogenic variants in *TBK1* cause FTD-ALS [44–46]. Importantly, elevated CSF levels of TNF-alpha are a core feature of FTD [6,47]. Building on these findings, in our bioinformatics “network”-based analysis, we found that both *LRRK2* and *TBKBP1* interact with genes within the *HLA* region (Fig 4). Further, physical interactions between *MAPT* and the *HLA* network are compatible with research suggesting that under different conditions reactive microglia can either drive or mitigate tau pathology [48,49]. *MAPT* mutations, which disrupt the normal binding of tau protein to tubulin, account for a large proportion of familial FTD cases [50]. Together, these findings suggest that *LRRK2*, *TBKBP1*, and *MAPT* may, at least in part, influence FTD pathogenesis via *HLA*-related mechanisms.

This study has limitations. Particularly, in the original datasets that form the basis of our analysis, diagnosis of FTD was established clinically. Given the clinical overlap among neurodegenerative diseases, we cannot exclude the potential influence of clinical misdiagnosis. As such, our findings would benefit from confirmation in large pathologically confirmed cohorts. In addition, given the complex interconnectedness of the *HLA* region (see Fig 4), we also were not able to define the specific gene or genes on Chr 6 responsible for our pleiotropic signal. However, given the number of *HLA* loci associated with increased FTD risk, it may be the case that no single *HLA* variant will be clinically informative; rather, an *additive combination* of these microglia-associated genetic variants may better inform FTD risk. This possibility is supported by our observation that the expression levels of FTD-immune shared genetic variants differ *on average* between FTD brains and controls, but with considerable overlap between the two groups, again suggesting that no single variant is likely to be the determinant of FTD risk (Fig 5). Further, we acknowledge the lack of transcriptomic and epigenetic data that would help to identify possible causal associations and mechanisms driving our pleotropic signal.

In conclusion, across a large cohort (total *n* = 192,886 cases and controls), we used pleiotropy between FTD-related disorders and immune-mediated disease to identify several genes within the *HLA* region that are expressed within microglia and differentially expressed in the brains of patients with FTD. Building on prior work [6,7], our results support a disease model in which immune dysfunction is central to the pathophysiology of a subset of FTD cases. These findings have important implications for future work in FTD focused on monitoring microglial activation as a marker of disease progression and investigating anti-inflammatory treatments as modifiers of disease outcome.

## Supporting information

S1 Acknowledgments (DOCX)

S1 Fig Fold enrichment plots of enrichment versus nominal −log_10_(*p*)-values (corrected for inflation) in corticobasal degeneration (CBD) below the standard genome-wide association study threshold of *p* < 5 × 10^−8^ as a function of significance of association with 6 immune-mediated diseases. The 6 immune-mediated diseases are Crohn disease (CD), ulcerative colitis (UC), type 1 diabetes (T1D), rheumatoid arthritis (RA), celiac disease (CeD), and psoriasis (PSOR). The levels of −log_10_(*p*) > 0, −log_10_(*p*) > 1, and −log_10_(*p*) > 2 correspond to *p* < 1, *p* < 0.1, and *p* < 0.01, respectively. The dark blue line indicates all SNPs.
(TIFF)

S2 Fig Fold enrichment plots of enrichment versus nominal −log_10_(*p*)-values (corrected for inflation) in progressive supranuclear palsy (PSP) below the standard genome-wide association study threshold of *p* < 5 × 10^−8^ as a function of significance of association with 6 immune-mediated diseases. The 6 immune-mediated diseases are Crohn disease (CD), ulcerative colitis (UC), type 1 diabetes (T1D), rheumatoid arthritis (RA), celiac disease (CeD), and psoriasis (PSOR). The levels of −log_10_(*p*) > 0, −log_10_(*p*) > 1, and −log_10_(*p*) > 2 correspond to *p* < 1, *p* < 0.1, and *p* < 0.01, respectively. The dark blue line indicates all SNPs.
(TIFF)

S3 Fig Fold enrichment plots of enrichment versus nominal −log_10_(*p*)-values (corrected for inflation) in amyotrophic lateral sclerosis (ALS) below the standard genome-wide association study threshold of *p* < 5 × 10^−8^ as a function of significance of association with 6 immune-mediated diseases. The 6 immune-mediated diseases are Crohn disease (CD), ulcerative colitis (UC), type 1 diabetes (T1D), rheumatoid arthritis (RA), celiac disease (CeD), and psoriasis (PSOR). The levels of −log_10_(*p*) > 0, −log_10_(*p*) > 1, and −log_10_(*p*) > 2 correspond to *p* < 1, *p* < 0.1, and *p* < 0.01, respectively. The dark blue line indicates all SNPs.
(TIFF)

S4 Fig “Conjunction” Manhattan plot of conjunction and conditional −log_10_(FDR) values for corticobasal degeneration (CBD) given 6 immune-mediated diseases. The 6 immune-mediated diseases are Crohn disease (CD; CBD|CD, red), ulcerative colitis (UC, CBD|UC, orange), type 1 diabetes (T1D, CBD|T1D, teal), rheumatoid arthritis (RA, CBD|RA, green), celiac disease (CeD, CBD|CeD, magenta), and psoriasis (PSOR, CBD|PSOR, blue). SNPs with conditional and conjunction −log_10_(FDR) > 1.3 (i.e., FDR < 0.05) are shown with large points. A black line around the large points indicates the most significant SNP in each linkage disequilibrium block, and this SNP was annotated with the closest gene, which is listed above the symbols in each locus.
(TIFF)

S5 Fig “Conjunction” Manhattan plot of conjunction and conditional −log_10_(FDR) values for progressive supranuclear palsy (PSP) given 6 immune-mediated diseases. The 6 immune-mediated diseases are Crohn disease (CD; PSP|CD, red), ulcerative colitis (UC, PSP|UC, orange), type 1 diabetes (T1D, PSP|T1D, teal), rheumatoid arthritis (RA, PSP|RA, green), celiac disease (CeD, PSP|CeD, magenta), and psoriasis (PSOR, PSP|PSOR, blue). SNPs with conditional and conjunction −log_10_(FDR) > 1.3 (i.e., FDR < 0.05) are shown with large points. A black line around the large points indicates the most significant SNP in each linkage disequilibrium block, and this SNP was annotated with the closest gene, which is listed above the symbols in each locus.
(TIFF)

S6 Fig “Conjunction” Manhattan plot of conjunction and conditional −log_10_(FDR) values for amyotrophic lateral sclerosis (ALS) given 6 immune-mediated diseases. The 6 immune-mediated diseases are Crohn disease (CD; ALS|CD, red), ulcerative colitis (UC, ALS|UC, orange), type 1 diabetes (T1D, ALS|T1D, teal), rheumatoid arthritis (RA, ALS|RA, green), celiac disease (CeD, ALS|CeD, magenta), and psoriasis (PSOR, ALS|PSOR, blue). SNPs with conditional and conjunction −log_10_(FDR) > 1.3 (i.e., FDR < 0.05) are shown with large points. A black line around the large points indicates the most significant SNP in each linkage disequilibrium block, and this SNP was annotated with the closest gene, which is listed above the symbols in each locus.
(TIFF)

S7 Fig Individual bar plots showing cell-type-specific expression for *LRRK2*. (TIFF)

S8 Fig Individual bar plots showing cell-type-specific expression for *PGBD5*. (TIFF)

S9 Fig Individual bar plots showing cell-type-specific expression for *TBKBP1*. (TIFF)

S1 Table Summary-data-based Mendelian randomization results. (DOCX)

S2 Table Overlapping loci between CBD and immune-mediated diseases at a conjunction FDR < 0.05. (DOCX)

S3 Table Overlapping loci between PSP and immune-mediated diseases at a conjunction FDR < 0.05. (DOCX)

S4 Table Overlapping loci between ALS and immune-mediated diseases at a conjunction FDR < 0.05. (DOCX)

S5 Table *cis*-eQTLs between FTD and immune-mediated disease shared risk SNPs and associated genes across a variety of tissues. (DOCX)

S6 Table Physical interaction and gene co-expression networks for the pleiotropic genes with significant *cis*-eQTLs. (DOCX)

## Abbreviations

ALS: amyotrophic lateral sclerosis
CBD: corticobasal degeneration
CD: Crohn disease
CeD: celiac disease
Chr: Chromosome
*cis*-eQTL: *cis*-expression quantitative trait locus
CNS: central nervous system
FDR: false discovery rate
FTD: frontotemporal dementia
FTD-U: frontotemporal lobar degeneration with ubiquitinated inclusions
GWAS: genome-wide association study
IFGC: International FTD-Genomics Consortium
LD: linkage disequilibrium
NIAGADS: National Institute on Aging Genetics of Alzheimer’s Disease Data Storage Site
PSOR: psoriasis; PSP, progressive supranuclear palsy
RA: rheumatoid arthritis
SMR: summary-data-based Mendelian randomization
SNP: single nucleotide polymorphism
T1D: type 1 diabetes
UC: ulcerative colitis
