# Correction: Immune-related genetic enrichment in frontotemporal dementia: An analysis of genome-wide association studies

**DOI:** 10.1371/journal.pmed.1002504

**Published:** 2018-01-29

**Authors:** Iris Broce, Celeste M. Karch, Natalie Wen, Chun C. Fan, Yunpeng Wang, Chin Hong Tan, Naomi Kouri, Owen A. Ross, Günter U. Höglinger, Ulrich Muller, John Hardy, International FTD-Genomics Consortium, Parastoo Momeni, Christopher P. Hess, William P. Dillon, Zachary A. Miller, Luke W. Bonham, Gil D. Rabinovici, Howard J. Rosen, Gerard D. Schellenberg, Andre Franke, Tom H. Karlsen, Jan H. Veldink, Raffaele Ferrari, Jennifer S. Yokoyama, Bruce L. Miller, Ole A. Andreassen, Anders M. Dale, Rahul S. Desikan, Leo P. Sugrue

The sixth author’s name appears incorrectly in the citation. The correct citation is: Broce I, Karch CM, Wen N, Fan CC, Wang Y, Tan CH, et al. (2018) Immune-related genetic enrichment in frontotemporal dementia: An analysis of genome-wide association studies. PLoS Med 15(1): e1002487. https://doi.org/10.1371/journal.pmed.1002487
