# Supplementary material for: Immune-related genetic enrichment in frontotemporal dementia: An analysis of genome-wide association studies
Source: PLoS Med. 2018 Jan 9;15(1):e1002487. doi: 10.1371/journal.pmed.1002487 (PMC5760014; doi:10.1371/journal.pmed.1002487)
Supplement: S1 Acknowledgments — (DOCX) [file pmed.1002487.s001.docx]

**S1 Acknowledgments.**Detailed acknowledgments for the International FTD-Genomics Consortium (IFGC).

Intramural funding from the National Institute of Neurological Disorders and Stroke (NINDS) and National Institute on Aging (NIA), the Wellcome/MRC Centre on Parkinson’s disease, Alzheimer’s Research UK (ARUK, Grant ARUK-PG2012-18) and by the office of the Dean of the School of Medicine, Department of Internal Medicine, at Texas Tech University Health Sciences Center.

We thank Mike Hubank and Kerra Pearce at the Genomic core facility at the Institute of Child Health (ICH), University College of London (UCL), for assisting RF in performing Illumina genotyping experiments (FTD-GWAS genotyping). This study utilized the high-performance computational capabilities of the Biowulf Linux cluster at the National Institutes of Health, Bethesda, Md. (<http://biowulf.nih.gov>). North American Brain Expression Consortium (NABEC) - The work performed by the North American Brain Expression Consortium (NABEC) was supported in part by the Intramural Research Program of the National Institute on Aging, National Institutes of Health, part of the US Department of Health and Human Services; project number ZIA AG000932-04. In addition this work was supported by a Research Grant from the Department of Defense, W81XWH-09-2-0128. UK Brain Expression Consortium (UKBEC) - This work performed by the UK Brain Expression Consortium (UKBEC) was supported by the MRC through the MRC Sudden Death Brain Bank (C.S.), by a Project Grant (G0901254 to J.H. and M.W.) and by a Fellowship award (G0802462 to M.R.). D.T. was supported by the King Faisal Specialist Hospital and Research Centre, Saudi Arabia. Computing facilities used at King's College London were supported by the National Institute for Health Research (NIHR) Biomedical Research Centre based at Guy's and St Thomas' NHS Foundation Trust and King's College London. We would like to thank AROS Applied Biotechnology AS company laboratories and Affymetrix for their valuable input. JBJK was supported by the National Health and Medical Resarch Council (NHMRC) Australia, Project Grants 510217 and 1005769; CDS was supported by NHMRC Project Grants 630428 and 1005769; PRS was supported by NHMRC Project Grants 510217 and 1005769 and acknowledges that DNA samples were prepared by Genetic Repositories Australia, supported by NHMRC Enabling Grant 401184; GMH was supported by NHMRC Research Fellowship 630434, Project Grant 1029538, Program Grant 1037746; JRH was supported by the Australian Research Council Federation Fellowship, NHMRC Project Grant 1029538, NHMRC Program Grant 1037746; OP was supported by NHMRC Career Development Fellowship 1022684, Project Grant 1003139. IH, AR and MB acknowledge the patients and controls who participated in this project and the Trinitat Port-Carbó and her family who are supporting Fundació ACE research programs. CC was supported by Grant P30-NS069329-01 and acknowledges that the recruitment and clinical characterization of research participants at Washington University were supported by NIH P50 AG05681, P01 AG03991, and P01 AG026276. LB and GB were supported by the Ricerca Corrente, Italian Ministry of Health; RG was supported by Fondazione CARIPLO 2009-2633, Ricerca Corrente, Italian Ministry of Health; GF was supported by Fondazione CARIPLO 2009-2633. ES was supported by the Italian Ministry of Health; CF was supported by Fondazione Cariplo; MS was supported from the Italian Ministry of Health (Ricerca Corrente); MLW was supported by Government funding of clinical research within NHS Sweden (ALF); KN was supported by Thure Carlsson Foundation; CN was supported by Swedish Alzheimer Fund. IRAM and GYRH were supported by CIHR (grant 74580) PARF (grant C06-01). JG was supported by the NINDS intramural research funds for FTD research. CMM was supported by Medical Research Council UK, Brains for Dementia Research, Alzheimer's Society, Alzheimer's Research UK, National Institutes for Health Research, Department of Health, Yvonne Mairy Bequest and acknowledges that tissue made available for this study was provided by the Newcastle Brain Tissue Resource, which was funded in part by grants G0400074 and G1100540 from the UK MRC, the Alzheimer’s Research Trust and Alzheimer’s Society through the Brains for Dementia Research Initiative and an NIHR Biomedical Research Centre Grant in Ageing and Health, and NIHR Biomedical Research Unit in Lewy Body Disorders. CMM was supported by the UK Department of Health and Medical Research Council and the Research was supported by the National Institute for Health Research Newcastle Biomedical Research Centre based at Newcastle Hospitals Foundation Trust and Newcastle University and acknowledges that the views expressed are those of the authors and not necessarily those of the NHS, the NIHR or the Department of Health; JA was supported by MRC, Dunhill Medical Trust, Alzheimer's Research UK; TDG was supported by Wellcome Trust Senior Clinical Fellow; IGM was supported by NIHR Biomedical Research Centre and Unit on Ageing Grants and acknowledges the National Institute for Health Research Newcastle Biomedical Research Centre based at Newcastle Hospitals Foundation Trust and Newcastle University. The views expressed are those of the author(s) and not necessarily those of the NHS, the NIHR or the Department of Health; AJT was supported by Medical Research Council, Alzheimer's Society, Alzheimer's Research UK, National Institutes for Health Research. EJ was supported by NIHR, Newcastle Biomedical Research Centre. PP, CR, SOC and EA were supported partially by FIMA (Foundation for Applied Medical Research); PP acknowledges Manuel Seijo-Martínez (Department of Neurology, Hospital do Salnés, Pontevedra, Spain), Ramon Rene, Jordi Gascon and Jaume Campdelacreu (Department of Neurology, Hospital de Bellvitge, Barcelona, Spain) for providing FTD DNA samples. RP, JDS, PA and AK were supported by German Federal Ministry of Education and Research (BMBF; grant number FKZ 01GI1007A – German FTLD consortium). IR was supported by Ministero dell'Istruzione, dell'Università e della Ricerca (MIUR) of Italy. PStGH was supported by the Canadian Institutes of Health Research, Wellcome Trust, Ontario Research Fund. FT was supported by the Italian Ministry of Health (ricerca corrente) and MIUR grant RBAP11FRE9; GR and GG were supported by the Italian Ministry of Health (ricerca corrente). JBR was supported by Camrbidge NIHR Biomedical Research Centre and Wellcome Trust (088324). JU, JC, SM were supported by the MRC Prion Unit core funding and acknowledge MRC UK, UCLH Biomedical Research Centre, Queen Square Dementia BRU; SM acknowledges the work of John Beck, Tracy Campbell, Gary Adamson, Ron Druyeh, Jessica Lowe, Mark Poulter. AD acknowledges the work of Benedikt Bader and of Manuela Neumann, Sigrun Roeber, Thomas Arzberger and Hans Kretzschmar†; VMVD and JQT were supported by Grants AG032953, AG017586 and AG010124; MG was supported by Grants AG032953, AG017586, AG010124 and NS044266; VMVD acknowledges EunRan Suh, PhD for assistance with sample handling and Elisabeth McCarty-Wood for help in selection of cases; JQT acknowledges Terry Schuck, John Robinson and Kevin Raible for assistance with neuropathological evaluation of cases. CVB and the Antwerp site were in part funded by the MetLife Foundation for Medical Research Award (to CVB), the Belgian Science Policy Office (BELSPO) Interuniversity Attraction Poles program; the Alzheimer Research Foundation (SAO-FRA); the Medical Foundation Queen Elisabeth (GSKE); the Flemish Government initiated Methusalem Excellence Program (to CVB); the Research Foundation Flanders (FWO) and the University of Antwerp Research Fund.. CVB, MC and JvdZ acknowledge the neurologists S Engelborghs, PP De Deyn, A Sieben, R Vandenberghe and the neuropathologist JJ Martin for the clinical and pathological diagnoses. CVB, MC and JvdZ further thank the personnel of the Genetic Service Facility of the VIB Department of Molecular Genetics (<http://www.vibgeneticservicefacility.be>) and the Antwerp Biobank of the Institute Born-Bunge for their expert support. IL and AB were supported by the program “Investissements d’avenir” ANR-10-IAIHU-06 and acknowledges the contribution of The French research network on FTLD/FTLD-ALS for the contribution in samples collection. BN is founded by Fondazione Cassa di Risparmio di Pistoia e Pescia (grant 2014.0365), SS is founded by the Cassa di Risparmio di Firenze (grant 2014.0310) and a grant from Ministry of Health n° RF-2010-2319722. JEN was supported by the Novo Nordisk Foundation, Denmark. MR was supported by the German National Genome Network (NGFN); German Ministry for Education and Research Grant Number 01GS0465. JDR, MNR, NCF and JDW were supported by an MRC programme grant and the Dementia Platform UK, the NIHR Queen Square Dementia Biomedical Research Unit (BRU) and the Leonard Wolfson Experimental Neurology Centre. MGS was supported by MRC grant n G0301152, Cambridge Biomedical Research Centre and acknowledges Mrs K Westmore for extracting DNA. HM was supported by the Motor Neuron Disease Association (Grant 6057). RR was supported by P50 AG016574, R01 NS080882, R01 NS065782, P50 NS72187 and the Consortium for Frontotemporal Dementia; DWD was supported by P50NS072187, P50AG016574, State of Florida Alzheimer Disease Initiative, & CurePSP, Inc.; NRGR, JEP, RCP, DK, BFB were supported by P50 AG016574; KAJ was supported by R01 AG037491; WWS was supported by NIH AG023501, AG019724, Consortium for Frontotemporal Dementia Research; BLM was supported by P50AG023501, P01AG019724, Consortium for FTD Research; HR was supported by AG032306. JCvS was supported by Stichting Dioraphte Foundation (11 02 03 00), Nuts Ohra Foundation (0801-69), Hersenstichting Nederland (BG 2010-02) and Alzheimer Nederland. CG and HHC acknowledge families, patients, clinicians including Dr Inger Nennesmo and Dr Vesna Jelic, Professor Laura Fratiglioni for control samples and Jenny Björkström, Håkan Thonberg, Charlotte Forsell, Anna-Karin Lindström and Lena Lilius for sample handling. CG was supported by Swedish Brain Power (SBP), the Strategic Research Programme in Neuroscience at Karolinska Institutet (StratNeuro), the regional agreement on medical training and clinical research (ALF) between Stockholm County Council and Karolinska Institutet, Swedish Alzheimer Foundation, Swedish Research Council, Karolinska Institutet PhD-student funding, King Gustaf V and Queen Victoria’s Free Mason Foundation. FP, AR, VD and FL acknowledge Labex DISTALZ. RF acknowledges the help and support of Mrs. June Howard at the Texas Tech University Health Sciences Center Office of Sponsored Programs for tremendous help in managing Material Transfer Agreement at TTUHSC.

**IFGC members and affiliations**

Raffaele Ferrari (UCL, Department of Molecular Neuroscience, Russell Square House, 9-12 Russell Square House, London, WC1B 5EH), Dena G Hernandez (Laboratory of Neurogenetics, National Institute on Aging, National Institutes of Health, 35 Convent Drive, Bethesda, MD 20892, USA; Reta Lila Weston Research Laboratories, Department of Molecular Neuroscience, UCL Institute of Neurology, London WC1N 3BG, UK), Michael A Nalls (Laboratory of Neurogenetics, National Institute on Aging, National Institutes of Health, 35 Convent Drive, Bethesda, MD 20892, USA), Jonathan D Rohrer (Reta Lila Weston Research Laboratories, Department of Molecular Neuroscience, UCL Institute of Neurology, Queen Square, London WC1N 3BG, UK; Dementia Research Centre, Department of Neurodegenerative Disease, UCL Institute of Neurology), Adaikalavan Ramasamy (Reta Lila Weston Research Laboratories, Department of Molecular Neuroscience, UCL Institute of Neurology, London WC1N 3BG, UK; Department of Medical and Molecular Genetics, King’s College London Tower Wing, Guy’s Hospital, London SE1 9RT, UK; The Jenner Institute, University of Oxford, Roosevelt Drive, Oxford OX3 7BQ, UK), John BJ Kwok (Neuroscience Research Australia, Sydney, NSW 2031, Australia; School of Medical Sciences, University of New South Wales, Sydney, NSW 2052, Australia), Carol Dobson-Stone (Neuroscience Research Australia, Sydney, NSW 2031, Australia; School of Medical Sciences, University of New South Wales, Sydney, NSW 2052, Australia), William S Brooks Neuroscience Research Australia, Sydney, NSW 2031, Australia; Prince of Wales Clinical School, University of New South Wales, Sydney, NSW 2052, Australia), Peter R Schofield (Neuroscience Research Australia, Sydney, NSW 2031, Australia; School of Medical Sciences, University of New South Wales, Sydney, NSW 2052, Australia), Glenda M Halliday (Neuroscience Research Australia, Sydney, NSW 2031, Australia; School of Medical Sciences, University of New South Wales, Sydney, NSW 2052, Australia), John R Hodges (Neuroscience Research Australia, Sydney, NSW 2031, Australia; School of Medical Sciences, University of New South Wales, Sydney, NSW 2052, Australia), Olivier Piguet (Neuroscience Research Australia, Sydney, NSW 2031, Australia; School of Medical Sciences, University of New South Wales, Sydney, NSW 2052, Australia), Lauren Bartley (Neuroscience Research Australia, Sydney, NSW 2031, Australia), Elizabeth Thompson (South Australian Clinical Genetics Service, SA Pathology (at Women's and Children's Hospital), North Adelaide, SA 5006, Australia; Department of Paediatrics, University of Adelaide, Adelaide, SA 5000, Australia), Eric Haan (South Australian Clinical Genetics Service, SA Pathology (at Women's and Children's Hospital), North Adelaide, SA 5006, Australia; Department of Paediatrics, University of Adelaide, Adelaide, SA 5000, Australia), Isabel Hernández (Research Center and Memory Clinic of Fundació ACE, Institut Català de Neurociències Aplicades, Barcelona, Spain), Agustín Ruiz (Research Center and Memory Clinic of Fundació ACE, Institut Català de Neurociències Aplicades, Barcelona, Spain), Mercè Boada (Research Center and Memory Clinic of Fundació ACE, Institut Català de Neurociències Aplicades, Barcelona, Spain), Barbara Borroni (Neurology Clinic, University of Brescia, Brescia, Italy), Alessandro Padovani (Neurology Clinic, University of Brescia, Brescia, Italy), Carlos Cruchaga (Department of Psychiatry, Washington University, St. Louis, MO, USA; Hope Center, Washington University School of Medicine, St. Louis, MO, USA), Nigel J Cairns (Hope Center, Washington University School of Medicine, St. Louis, MO, USA; Department of Pathology and Immunology, Washington University, St. Louis, MO, USA), Luisa Benussi (Molecular Markers Laboratory, IRCCS Istituto Centro San Giovanni di Dio Fatebenefratelli,Brescia, Italy), Giuliano Binetti (MAC Memory Clinic, IRCCS Istituto Centro San Giovanni di Dio Fatebenefratelli,Brescia, Italy), Roberta Ghidoni (Molecular Markers Laboratory, IRCCS Istituto Centro San Giovanni di Dio Fatebenefratelli, Brescia, Italy), Gianluigi Forloni (Biology of Neurodegenerative Disorders, IRCCS Istituto di Ricerche Farmacologiche, "Mario Negri",Milano, Italy), Diego Albani (Biology of Neurodegenerative Disorders, IRCCS Istituto di Ricerche Farmacologiche "Mario Negri", Milano, Italy), Daniela Galimberti (University of Milan, Milan, Italy; Fondazione Cà Granda, IRCCS Ospedale Maggiore Policlinico, via F. Sforza 35, 20122, Milan, Italy), Chiara Fenoglio (University of Milan, Milan, Italy; Fondazione Cà Granda, IRCCS Ospedale Maggiore Policlinico, via F. Sforza 35, 20122, Milan, Italy), Maria Serpente (University of Milan, Milan, Italy; Fondazione Cà Granda, IRCCS Ospedale Maggiore Policlinico, via F. Sforza 35, 20122, Milan, Italy), Elio Scarpini (University of Milan, Milan, Italy; Fondazione Cà Granda, IRCCS Ospedale Maggiore Policlinico, via F. Sforza 35, 20122, Milan, Italy), Jordi Clarimón (Memory Unit, Neurology Department and Sant Pau Biomedical Research Institute, Hospital de la Santa Creu i Sant Pau, Universitat Autònoma de Barcelona, Barcelona, Spain; Center for Networker Biomedical Research in Neurodegenerative Diseases (CIBERNED), Madrid, Spain), Alberto Lleó (Memory Unit, Neurology Department and Sant Pau Biomedical Research Institute, Hospital de la Santa Creu i Sant Pau, Universitat Autònoma de Barcelona, Barcelona, Spain; Center for Networker Biomedical Research in Neurodegenerative Diseases (CIBERNED), Madrid, Spain), Rafael Blesa (Memory Unit, Neurology Department and Sant Pau Biomedical Research Institute, Hospital de la Santa Creu i Sant Pau, Universitat Autònoma de Barcelona, Barcelona, Spain; Center for Networker Biomedical Research in Neurodegenerative Diseases (CIBERNED), Madrid, Spain); Maria Landqvist Waldö (Unit of Geriatric Psychiatry, Department of Clinical Sciences, Lund University, Lund, Sweden), Karin Nilsson (Unit of Geriatric Psychiatry, Department of Clinical Sciences, Lund University, Lund, Sweden), Christer Nilsson (Clinical Memory Research Unit, Department of Clinical Sciences, Lund University, Lund, Sweden), Ian RA Mackenzie (Department of Pathology and Laboratory Medicine, University of British Columbia, Vancouver, Canada), Ging-Yuek R Hsiung (Division of Neurology, University of British Columbia, Vancouver, Canada), David MA Mann (Institute of Brain, Behaviour and Mental Health, University of Manchester, Salford Royal Hospital, Stott Lane, Salford, M6 8HD, UK), Jordan Grafman (Rehabilitation Institute of Chicago, Departments of Physical Medicine and Rehabilitation, Psychiatry, and Cognitive Neurology & Alzheimer's Disease Center; Feinberg School of Medicine, Northwestern University, Chicago, USA; Department of Psychology, Weinberg College of Arts and Sciences, Northwestern University, , Chicago, USA), Christopher M Morris (Newcastle Brain Tissue Resource, Institute for Ageing, Newcastle University, NE4 5PL, Newcastle upon Tyne, UK; Newcastle University, Institute of Neuroscience and Institute for Ageing, Campus for Ageing and Vitality, NE4 5PL, Newcastle upon Tyne, UK; Institute of Neuroscience, Newcastle University Medical School, Framlington Place NE2 4HH, Newcastle upon Tyne, UK), Johannes Attems (Newcastle University, Institute of Neuroscience and Institute for Ageing, Campus for Ageing and Vitality, NE4 5PL, Newcastle upon Tyne, UK); Timothy D Griffiths (Institute of Neuroscience, Newcastle University Medical School, Framlington Place, NE2 4HH, Newcastle upon Tyne, UK), Ian G McKeith (Newcastle University, Institute of Neuroscience and Institute for Ageing, Campus for Ageing and Vitality, Newcastle University, NE4 5PL, Newcastle upon Tyne, UK), Alan J Thomas (Newcastle University, Institute of Neuroscience and Institute for Ageing, Campus for Ageing and Vitality, NE4 5PL, Newcastle upon Tyne, UK), Pietro Pietrini (IMT School for Advanced Studies, Lucca, Lucca, Italy), Edward D Huey (Taub Institute, Departments of Psychiatry and Neurology, Columbia University, 630 West 168th Street New York, NY 10032), Eric M Wassermann (Behavioral Neurology Unit, National Insititute of Neurological Disorders and Stroke, National Insititutes of Health, 10 CENTER DR MSC 1440, Bethesda, MD 20892-1440), Atik Baborie (Department of Laboratory Medicine & Pathology, Walter Mackenzie Health Sciences Centre, 8440 - 112 St, University of Alberta Edmonton, Alberta T6G 2B7, Canada), Evelyn Jaros (Newcastle University, Institute for Ageing and Health, Campus for Ageing and Vitality, NE4 5PL, Newcastle upon Tyne, UK), Michael C Tierney (Behavioral Neurology Unit, National Insititute of Neurological Disorders and Stroke, National Insititutes of Health, 10 CENTER DR MSC 1440, Bethesda, MD 20892-1440), Pau Pastor (Center for Networker Biomedical Research in Neurodegenerative Diseases (CIBERNED), Madrid, Spain; Neurogenetics Laboratory, Division of Neurosciences, Center for Applied Medical Research, Universidad de Navarra, Pamplona, Spain; Department of Neurology, Clínica Universidad de Navarra, University of Navarra School of Medicine, Pamplona, Spain), Cristina Razquin (Neurogenetics Laboratory, Division of Neurosciences, Center for Applied Medical Research, Universidad de Navarra, Pamplona, Spain), Sara Ortega-Cubero (Center for Networker Biomedical Research in Neurodegenerative Diseases (CIBERNED), Madrid, Spain; Neurogenetics Laboratory, Division of Neurosciences, Center for Applied Medical Research, Universidad de Navarra, Pamplona, Spain), Elena Alonso (Neurogenetics Laboratory, Division of Neurosciences, Center for Applied Medical Research, Universidad de Navarra, Pamplona, Spain), Robert Perneczky (Neuroepidemiology and Ageing Research Unit, School of Public Health, Faculty of Medicine, The Imperial College of Science, Technology and Medicine, London W6 8RP, UK; West London Cognitive Disorders Treatment and Research Unit, West London Mental Health Trust, London TW8 8 DS, UK; Department of Psychiatry and Psychotherapy, Technische Universität München, Munich, 81675 Germany), Janine Diehl-Schmid (Department of Psychiatry and Psychotherapy, Technische Universität München, Munich, 81675 Germany), Panagiotis Alexopoulos (Department of Psychiatry and Psychotherapy, Technische Universität München, Munich, 81675 Germany), Alexander Kurz (Department of Psychiatry and Psychotherapy, Technische Universität München, Munich, 81675 Germany), Innocenzo Rainero (Neurology I, Department of Neuroscience, University of Torino, Italy, A.O. Città della Salute e della Scienza di Torino, Torino, Italy), Elisa Rubino (Neurology I, Department of Neuroscience, University of Torino, Italy, A.O. Città della Salute e della Scienza di Torino, Torino, Italy), Lorenzo Pinessi (Neurology I, Department of Neuroscience, University of Torino, Italy, A.O. Città della Salute e della Scienza di Torino, Torino, Italy), Ekaterina Rogaeva (Tanz Centre for Research in Neurodegenerative Diseases, University of Toronto 60 Leonard Street, Toronto, Ontario, Canada, M5T 2S8), Peter St George-Hyslop (Tanz Centre for Research in Neurodegenerative Diseases, University of Toronto, 60 Leonard Street, Toronto, Ontario, Canada, M5T 2S8; Cambridge Institute for Medical Research, and the Department of Clinical Neurosciences, University of Cambridge, Hills Road, Cambridge, UK CB2 0XY), Giacomina Rossi (Division of Neurology V and Neuropathology, Fondazione IRCCS Istituto Neurologico Carlo Besta, 20133 Milano, Italy), Fabrizio Tagliavini (Division of Neurology V and Neuropathology, Fondazione IRCCS Istituto Neurologico Carlo Besta, 20133 Milano, Italy), Giorgio Giaccone (Division of Neurology V and Neuropathology, Fondazione IRCCS Istituto Neurologico Carlo Besta, 20133 Milano, Italy), James B Rowe (Cambridge University Department of Clinical Neurosciences, Cambridge, CB2 0SZ, UK; MRC Cognition and Brain Sciences Unit, Cambridge, CB2 7EF, UK; Behavioural and Clinical Neuroscience Institute, Cambridge, CB2 3EB, UK), Johannes CM Schlachetzki (University of California San Diego, Department of Cellular & Molecular Medicine, 9500 Gilman Drive, La Jolla, CA, 92093), James Uphill (MRC Prion Unit, Department of Neurodegenerative Disease, UCL Institute of Neurology, Queen Square House, Queen Square, London, WC1N 3BG), John Collinge (MRC Prion Unit, Department of Neurodegenerative Disease, UCL Institute of Neurology Queen Square House, Queen Square, London, WC1N 3BG), Simon Mead (MRC Prion Unit, Department of Neurodegenerative Disease, UCL Institute of Neurology, Queen Square House, Queen Square, London, WC1N 3BG), Adrian Danek (Neurologische Klinik und Poliklinik, Ludwig-Maximilians-Universität, Munich, Germany, German Center for Neurodegenerative Diseases (DZNE)), Vivianna M Van Deerlin (University of Pennsylvania Perelman School of Medicine, Department of Pathology and Laboratory Medicine, Philadelphia, PA, USA), Murray Grossman (University of Pennsylvania Perelman School of Medicine, Department of Neurology and Penn Frontotemporal Degeneration Center, Philadelphia, PA, USA), John Q Trojanowski (University of Pennsylvania Perelman School of Medicine, Department of Pathology and Laboratory Medicine, Philadelphia, PA, USA), Julie van der Zee (Neurodegenerative Brain Diseases group, Department of Molecular Genetics, VIB, Antwerp, Belgium; Laboratory of Neurogenetics, Institute Born-Bunge, University of Antwerp, Antwerp, Belgium), Marc Cruts (Neurodegenerative Brain Diseases group, Department of Molecular Genetics, VIB, Antwerp, Belgium; Laboratory of Neurogenetics, Institute Born-Bunge, University of Antwerp, Antwerp, Belgium), Christine Van Broeckhoven (Neurodegenerative Brain Diseases group, Department of Molecular Genetics, VIB, Antwerp, Belgium; Laboratory of Neurogenetics, Institute Born-Bunge, University of Antwerp, Antwerp, Belgium), Stefano F Cappa (Neurorehabilitation Unit, Dept. Of Clinical Neuroscience, Vita-Salute University and San Raffaele Scientific Institute, Milan, Italy), Isabelle Leber (Inserm, UMR_S975, CRICM; UPMC Univ Paris 06, UMR_S975; CNRS UMR 7225, F-75013, Paris, France; AP-HP, Hôpital de la Salpêtrière, Département de neurologie-centre de références des démences rares, F-75013, Paris, France), Didier Hannequin (Service de Neurologie, Inserm U1079, CNR-MAJ, Rouen University Hospital, Rouen, France), Véronique Golfier (Service de neurologie, CH Saint Brieuc, France), Martine Vercelletto (Service de neurologie, CHU Nantes, France), Alexis Brice (Inserm, UMR_S975, CRICM; UPMC Univ Paris 06, UMR_S975; CNRS UMR 7225, F-75013, Paris, France; AP-HP, Hôpital de la Salpêtrière, Département de neurologie-centre de références des démences rares, F-75013, Paris, France), Benedetta Nacmias (Department of Neurosciences, Psychology, Drug Research and Child Health (NEUROFARBA) University of Florence, Florence, Italy), Sandro Sorbi (Department of Neurosciences, Psychology, Drug Research and Child Health (NEUROFARBA), University of Florence and IRCCS "Don Carlo Gnocchi" Firenze, Florence, Italy), Silvia Bagnoli (Department of Neurosciences, Psychology, Drug Research and Child Health (NEUROFARBA), University of Florence, Florence, Italy), Irene Piaceri (Department of Neurosciences, Psychology, Drug Research and Child Health (NEUROFARBA), University of Florence, Florence, Italy), Jørgen E Nielsen (Danish Dementia Research Centre, Neurogenetics Clinic, Department of Neurology, Rigshospitalet, Copenhagen University Hospital, Copenhagen, Denmark; Department of Cellular and Molecular Medicine, Section of Neurogenetics, The Panum Institute, University of Copenhagen, Copenhagen, Denmark), Lena E Hjermind (Danish Dementia Research Centre, Neurogenetics Clinic, Department of Neurology, Rigshospitalet, Copenhagen University Hospital, Copenhagen, Denmark; Department of Cellular and Molecular Medicine, Section of Neurogenetics, The Panum Institute, University of Copenhagen, Copenhagen, Denmark), Matthias Riemenschneider (Saarland University Hospital, Department for Psychiatry & Psychotherapy, Kirrberger Str.1, Bld.90, 66421 Homburg/Saar, Germany; Saarland University, Laboratory for Neurogenetics, Kirrberger Str.1, Bld.90, 66421 Homburg/Saar, Germany), Manuel Mayhaus (Saarland University, Laboratory for Neurogenetics, Kirrberger Str.1, Bld.90, 66421 Homburg/Saar, Germany), Bernd Ibach (University Regensburg, Department of Psychiatry, Psychotherapy and Psychosomatics, Universitätsstr. 84, 93053 Regensburg, Germany), Gilles Gasparoni (Saarland University, Laboratory for Neurogenetics, Kirrberger Str.1, Bld.90, 66421 Homburg/Saar, Germany), Sabrina Pichler (Saarland University, Laboratory for Neurogenetics, Kirrberger Str.1, Bld.90, 66421 Homburg/Saar, Germany), Wei Gu (Saarland University, Laboratory for Neurogenetics, Kirrberger Str.1, Bld.90, 66421 Homburg/Saar, Germany; Luxembourg Centre For Systems Biomedicine (LCSB), University of Luxembourg 7, avenue des Hauts-Fourneaux, 4362 Esch-sur-Alzette, Luxembourg), Martin N Rossor (Dementia Research Centre, Department of Neurodegenerative Disease, UCL Institute of Neurology, Queen Square, London, WC1N 3BG), Nick C Fox (Dementia Research Centre, Department of Neurodegenerative Disease, UCL Institute of Neurology Queen Square, London, WC1N 3BG), Jason D Warren (Dementia Research Centre, Department of Neurodegenerative Disease, UCL Institute of Neurology, Queen Square, London, WC1N 3BG), Maria Grazia Spillantini (University of Cambridge, Department of Clinical Neurosciences, John Van Geest Brain Repair Centre, Forvie Site, Robinson way, Cambridge CB2 0PY), Huw R Morris (UCL, Department of Molecular Neuroscience, Russell Square House, 9-12 Russell Square House, London, WC1B 5EH), Patrizia Rizzu (German Center for Neurodegenerative Diseases-Tübingen, Otfried Muellerstrasse 23, Tuebingen 72076, Germany), Peter Heutink (German Center for Neurodegenerative Diseases-Tübingen, Otfried Muellerstrasse 23, Tuebingen 72076, Germany), Julie S Snowden (Institute of Brain, Behaviour and Mental Health, Faculty of Medical and Human Sciences, University of Manchester, Manchester, UK), Sara Rollinson (Institute of Brain, Behaviour and Mental Health, Faculty of Medical and Human Sciences, University of Manchester, Manchester, UK), Anna Richardson (Salford Royal Foundation Trust, Faculty of Medical and Human Sciences, University of Manchester, Manchester, UK), Alexander Gerhard (Institute of Brain, Behaviour and Mental Health, The University of Manchester, 27 Palatine Road, Withington, Manchester, M20 3LJ, UK), Amalia C Bruni (Regional Neurogenetic Centre, ASPCZ, Lamezia Terme, Italy), Raffaele Maletta (Regional Neurogenetic Centre, ASPCZ, Lamezia Terme, Italy), Francesca Frangipane (Regional Neurogenetic Centre, ASPCZ, Lamezia Terme, Italy), Chiara Cupidi (Regional Neurogenetic Centre, ASPCZ, Lamezia Terme, Italy), Livia Bernardi (Regional Neurogenetic Centre, ASPCZ, Lamezia Terme, Italy), Maria Anfossi (Regional Neurogenetic Centre, ASPCZ, Lamezia Terme, Italy), Maura Gallo (Regional Neurogenetic Centre, ASPCZ, Lamezia Terme, Italy), Maria Elena Conidi Regional Neurogenetic Centre, ASPCZ, Lamezia Terme, Italy), Nicoletta Smirne (Regional Neurogenetic Centre, ASPCZ, Lamezia Terme, Italy), Rosa Rademakers (Department of Neuroscience, Mayo Clinic Jacksonville, 4500 San Pablo Road, Jacksonville, FL 32224), Matt Baker (Department of Neuroscience, Mayo Clinic Jacksonville, 4500 San Pablo Road, Jacksonville, FL 32224), Dennis W Dickson (Department of Neuroscience, Mayo Clinic Jacksonville, 4500 San Pablo Road, Jacksonville, FL 32224), Neill R Graff-Radford (Department of Neurology, Mayo Clinic Jacksonville, 4500 San Pablo Road, Jacksonville, FL 32224), Ronald C Petersen (Department of Neurology, Mayo Clinic Rochester, 2001st street SW Rochester MN 5905), David Knopman (Department of Neurology, Mayo Clinic Rochester, 2001st street SW Rochester MN 5905), Keith A Josephs (Department of Neurology, Mayo Clinic Rochester, 2001st street SW Rochester MN 5905), Bradley F Boeve (Department of Neurology, Mayo Clinic Rochester, 2001st street SW Rochester MN 5905), Joseph E Parisi (Department of Pathology, Mayo Clinic Rochester, 2001st street SW Rochester MN 5905), William W Seeley (Department of Neurology, Box 1207, University of California, San Francisco, CA 94143, USA), Bruce L Miller (Memory and Aging Center, Department of Neurology, University of California, San Francisco, CA 94158, USA), Anna M Karydas (Memory and Aging Center, Department of Neurology, University of California, San Francisco, CA 94158, USA), Howard Rosen (Memory and Aging Center, Department of Neurology, University of California, San Francisco, CA 94158, USA), John C van Swieten (Department of Neurology, Erasmus Medical Centre, Rotterdam, The Netherlands; Department of Medical Genetics, VU university Medical Centre, Amsterdam, The Netherlands), Elise GP Dopper (Department of Neurology, Erasmus Medical Centre, Rotterdam, The Netherlands), Harro Seelaar (Department of Neurology, Erasmus Medical Centre, Rotterdam, The Netherlands), Yolande AL Pijnenburg (Alzheimer Centre and department of neurology, VU University medical centre, Amsterdam, The Netherlands), Philip Scheltens (Alzheimer Centre and department of neurology, VU University medical centre, Amsterdam, The Netherlands), Giancarlo Logroscino (Department of Basic Medical Sciences, Neurosciences and Sense Organs of the "Aldo Moro" University of Bari, Bari, Italy), Rosa Capozzo (Department of Basic Medical Sciences, Neurosciences and Sense Organs of the "Aldo Moro" University of Bari, Bari, Italy), Valeria Novelli (Medical Genetics Unit, Fondazione Policlinico Universitario A. Gemelli, Rome, Italy), Annibale A Puca (Cardiovascular Research Unit, IRCCS Multimedica, Milan, Italy; Department of Medicine and Surgery, University of Salerno, Baronissi (SA), Italy), Massimo Franceschi (Neurology Dept, IRCCS Multimedica, Milan, Italy), Alfredo Postiglione (Department of Clinical Medicine and Surgery, University of Naples Federico II, Naples, Italy), Graziella Milan (Geriatric Center Frullone- ASL Napoli 1 Centro, Naples, Italy), Paolo Sorrentino (Geriatric Center Frullone- ASL Napoli 1 Centro, Naples, Italy), Mark Kristiansen (UCL Genomics, Institute of Child Health (ICH), UCL, London, UK), Huei-Hsin Chiang (Karolinska Institutet, Dept NVS, Alzheimer Research Center, Novum, SE-141 57, Stockholm, Sweden; Dept of Geriatric Medicine, Genetics Unit, M51, Karolinska University Hospital, SE-14186, Stockholm), Caroline Graff (Karolinska Institutet, Dept NVS, Alzheimer Research Center, Novum, SE-141 57, Stockholm, Sweden; Dept of Geriatric Medicine, Genetics Unit, M51, Karolinska University Hospital, SE-14186, Stockholm), Florence Pasquier (Univ Lille, Inserm 1171, DISTALZ, CHU 59000 Lille, France), Adeline Rollin (Univ Lille, Inserm 1171, DISTALZ, CHU 59000 Lille, France), Vincent Deramecourt (Univ Lille, Inserm 1171, DISTALZ, CHU 59000 Lille, France), Thibaud Lebouvier (Univ Lille, Inserm 1171, DISTALZ, CHU 59000 Lille, France), Dimitrios Kapogiannis (National Institute on Aging (NIA/NIH), 3001 S. Hanover St, NM 531, Baltimore, MD, 21230), Luigi Ferrucci (Clinical Research Branch, National Institute on Aging, Baltimore, MD, USA), Stuart Pickering-Brown (Institute of Brain, Behaviour and Mental Health, Faculty of Medical and Human Sciences, University of Manchester, Manchester, UK), Andrew B Singleton (Laboratory of Neurogenetics, National Institute on Aging, National Institutes of Health, Building 35, Room 1A215, 35 Convent Drive, Bethesda, MD 20892, USA), John Hardy (UCL, Department of Molecular Neuroscience, Russell Square House, 9-12 Russell Square House, London, WC1B 5EH), Parastoo Momeni (Laboratory of Neurogenetics, Department of Internal Medicine, Texas Tech University Health Science Center, 4th street, Lubbock, Texas 79430, USA)
