# Supplementary figures and images for: Immune-related genetic enrichment in frontotemporal dementia: An analysis of genome-wide association studies
Source: PLoS Med. 2018 Jan 9;15(1):e1002487. doi: 10.1371/journal.pmed.1002487 (PMC5760014; doi:10.1371/journal.pmed.1002487)

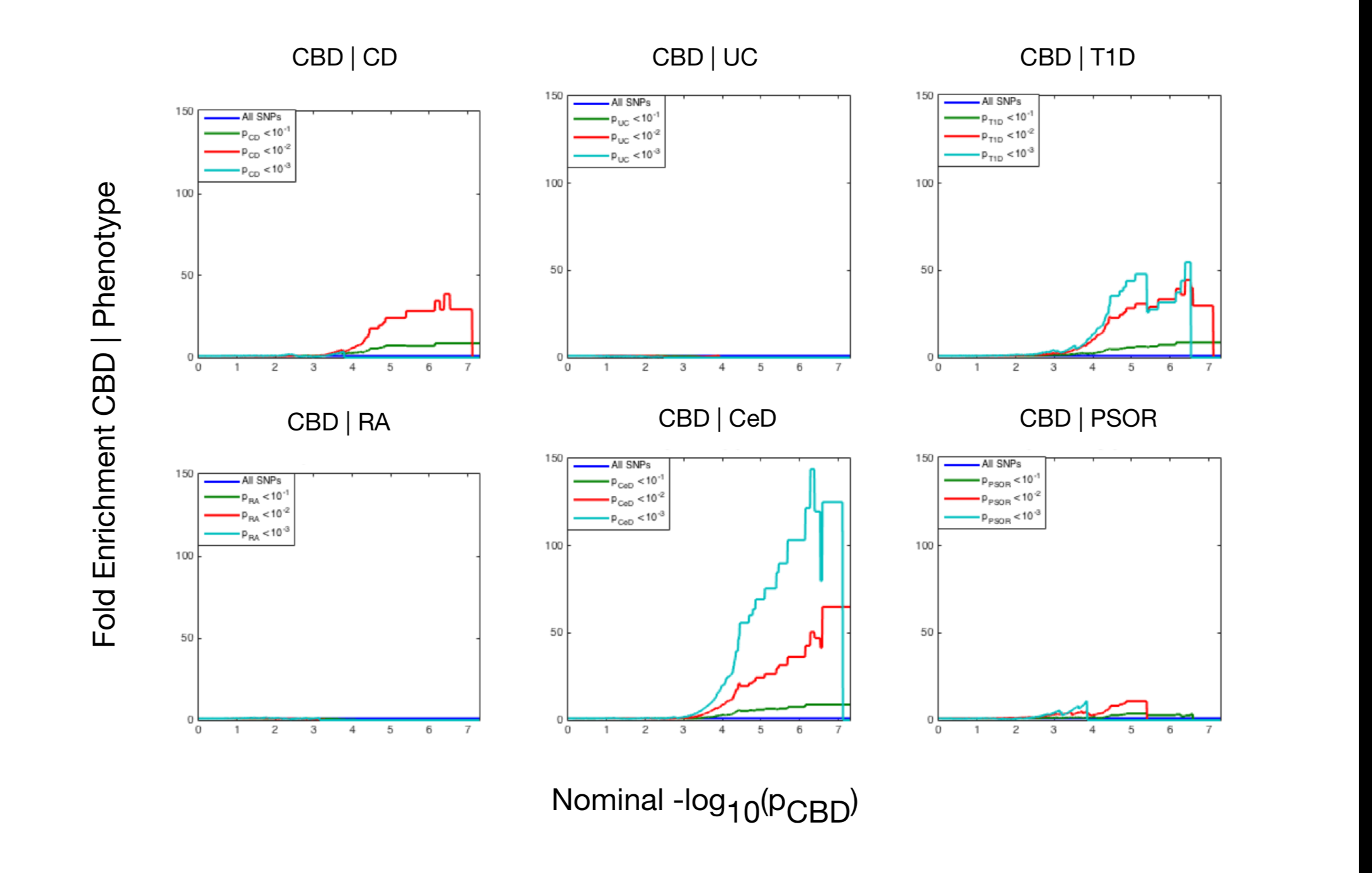

Supplement: S1 Fig — The 6 immune-mediated diseases are Crohn disease (CD), ulcerative colitis (UC), type 1 diabetes (T1D), rheumatoid arthritis (RA), celiac disease (CeD), and psoriasis (PSOR). The levels of −log10(p) > 0, −log10(p) > 1, and −log10(p) > 2 correspond to p < 1, p < 0.1, and p < 0.01, respectively. The dark blue line indicates all SNPs. (TIFF) [file pmed.1002487.s002.tiff]

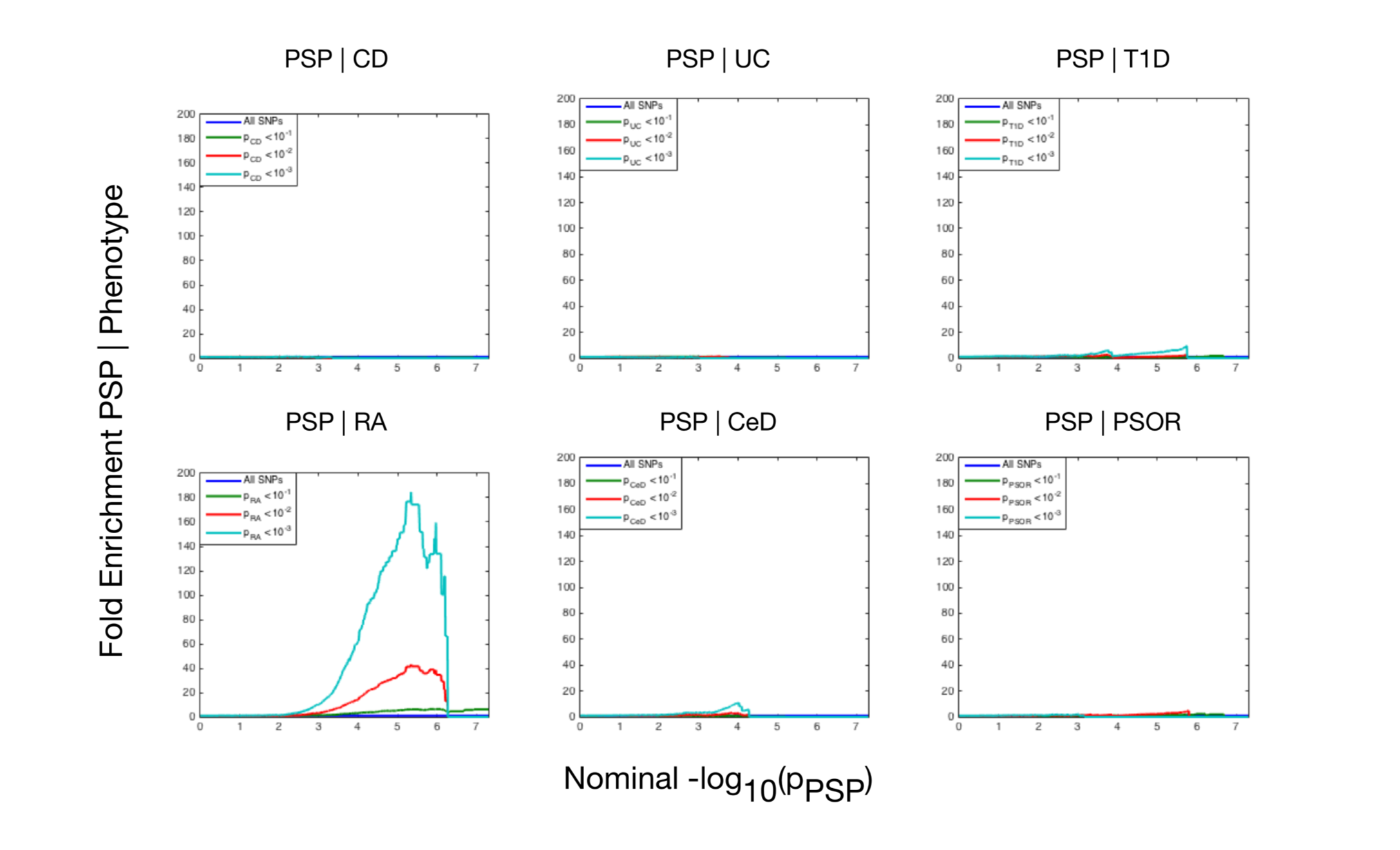

Supplement: S2 Fig — The 6 immune-mediated diseases are Crohn disease (CD), ulcerative colitis (UC), type 1 diabetes (T1D), rheumatoid arthritis (RA), celiac disease (CeD), and psoriasis (PSOR). The levels of −log10(p) > 0, −log10(p) > 1, and −log10(p) > 2 correspond to p < 1, p < 0.1, and p < 0.01, respectively. The dark blue line indicates all SNPs. (TIFF) [file pmed.1002487.s003.tiff]

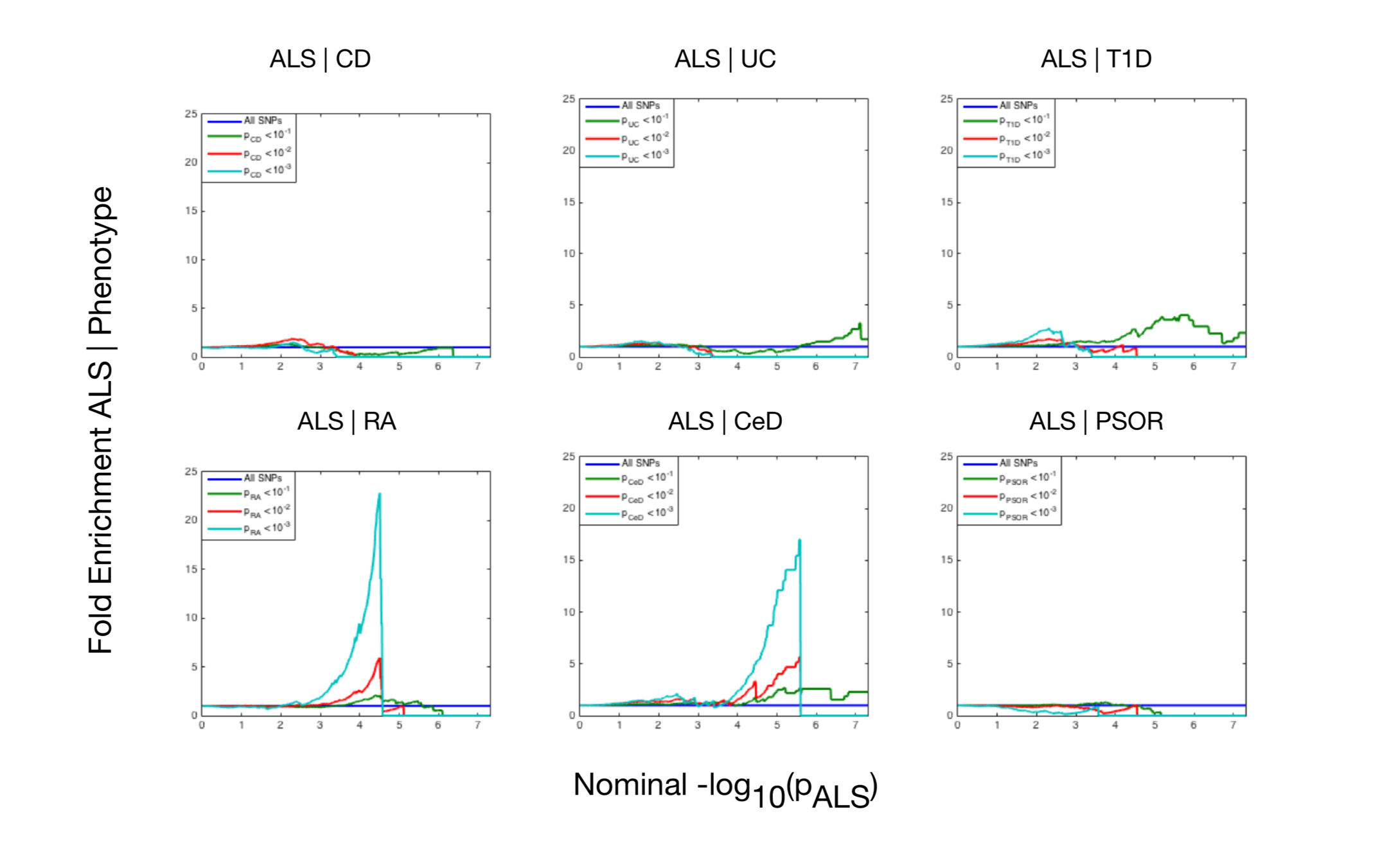

Supplement: S3 Fig — The 6 immune-mediated diseases are Crohn disease (CD), ulcerative colitis (UC), type 1 diabetes (T1D), rheumatoid arthritis (RA), celiac disease (CeD), and psoriasis (PSOR). The levels of −log10(p) > 0, −log10(p) > 1, and −log10(p) > 2 correspond to p < 1, p < 0.1, and p < 0.01, respectively. The dark blue line indicates all SNPs. (TIFF) [file pmed.1002487.s004.tiff]

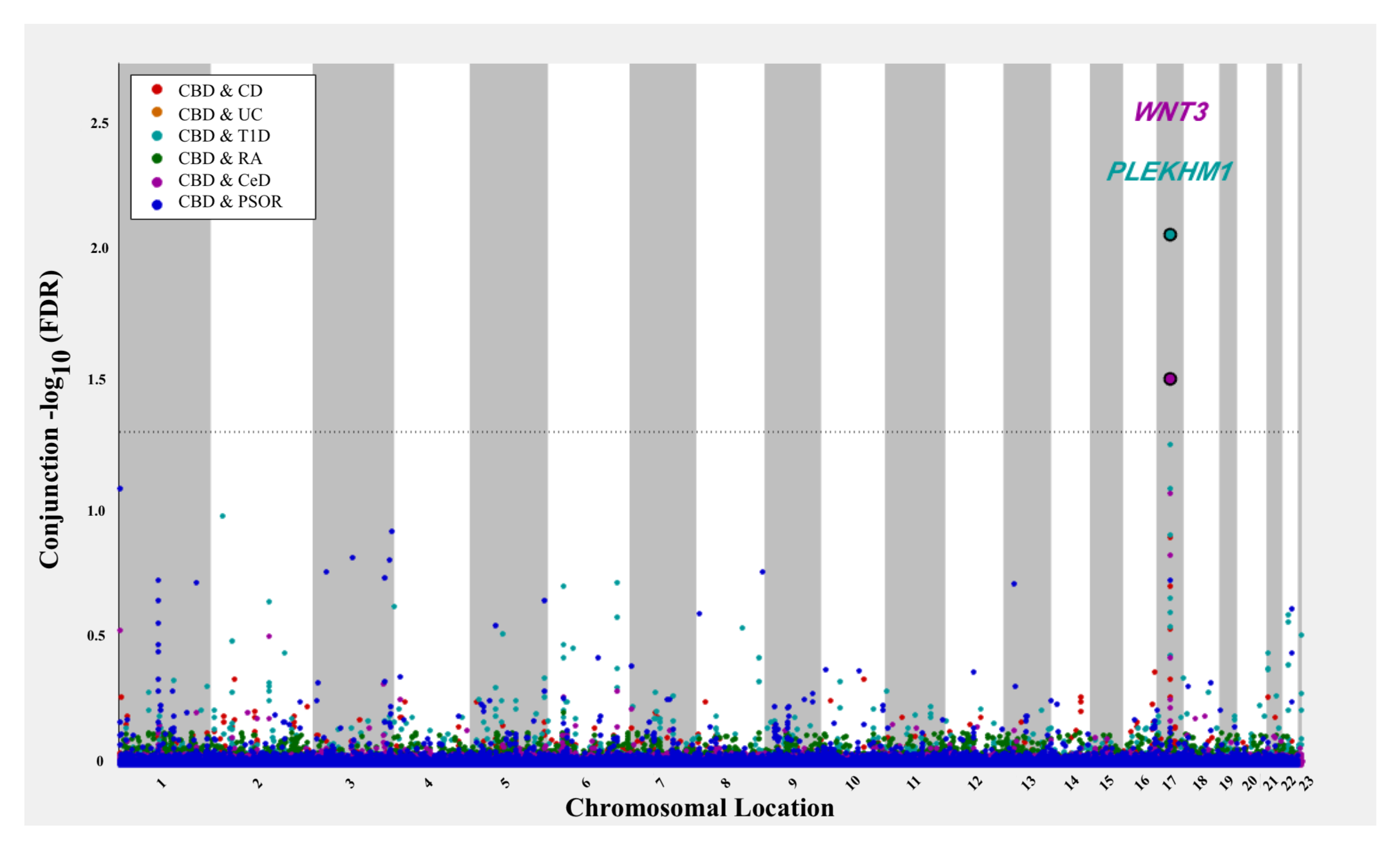

Supplement: S4 Fig — The 6 immune-mediated diseases are Crohn disease (CD; CBD|CD, red), ulcerative colitis (UC, CBD|UC, orange), type 1 diabetes (T1D, CBD|T1D, teal), rheumatoid arthritis (RA, CBD|RA, green), celiac disease (CeD, CBD|CeD, magenta), and psoriasis (PSOR, CBD|PSOR, blue). SNPs with conditional and conjunction −log10(FDR) > 1.3 (i.e., FDR < 0.05) are shown with large points. A black line around the large points indicates the most significant SNP in each linkage disequilibrium block, and this SNP was annotated with the closest gene, which is listed above the symbols in each locus. (TIFF) [file pmed.1002487.s005.tiff]

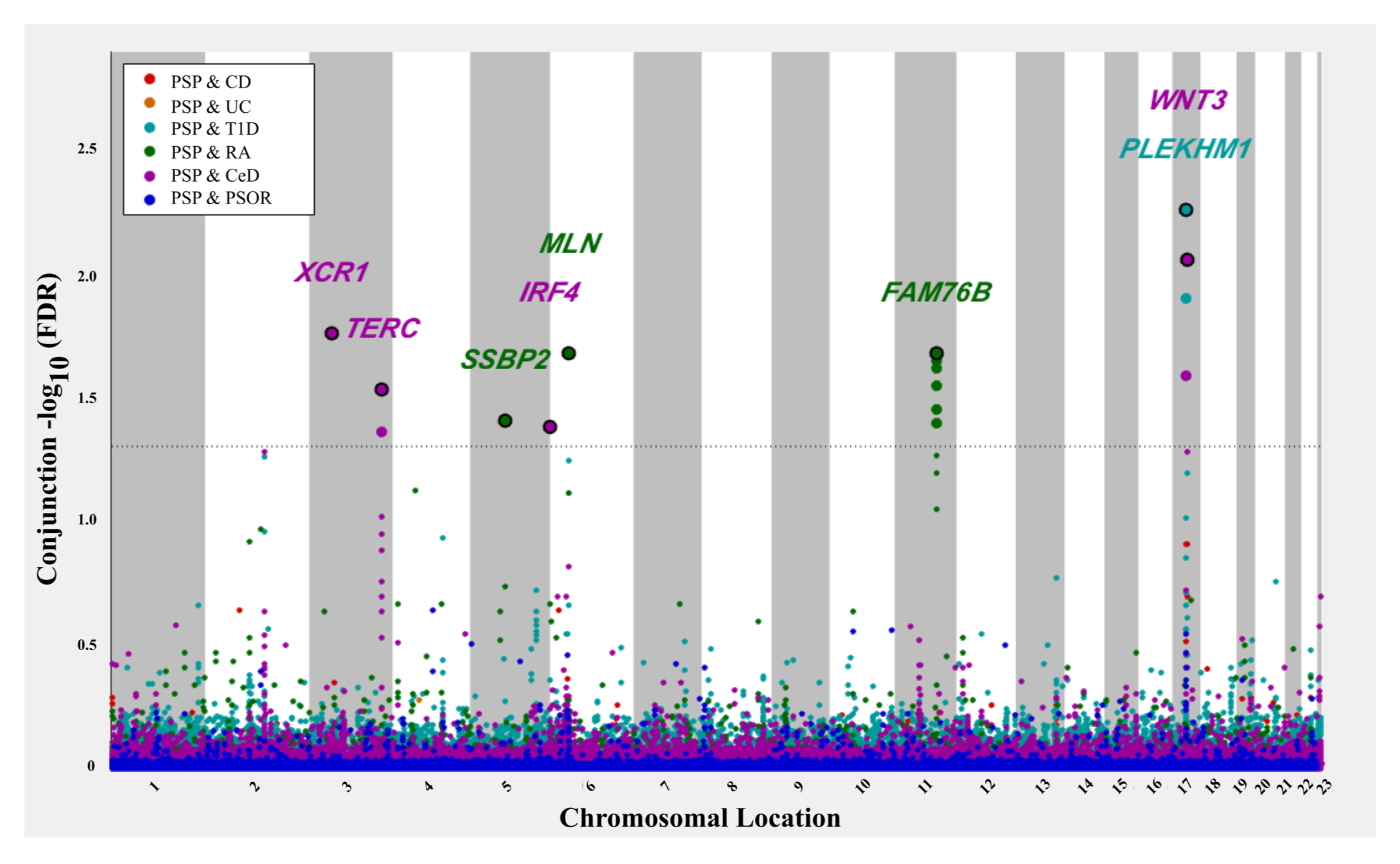

Supplement: S5 Fig — The 6 immune-mediated diseases are Crohn disease (CD; PSP|CD, red), ulcerative colitis (UC, PSP|UC, orange), type 1 diabetes (T1D, PSP|T1D, teal), rheumatoid arthritis (RA, PSP|RA, green), celiac disease (CeD, PSP|CeD, magenta), and psoriasis (PSOR, PSP|PSOR, blue). SNPs with conditional and conjunction −log10(FDR) > 1.3 (i.e., FDR < 0.05) are shown with large points. A black line around the large points indicates the most significant SNP in each linkage disequilibrium block, and this SNP was annotated with the closest gene, which is listed above the symbols in each locus. (TIFF) [file pmed.1002487.s006.tiff]

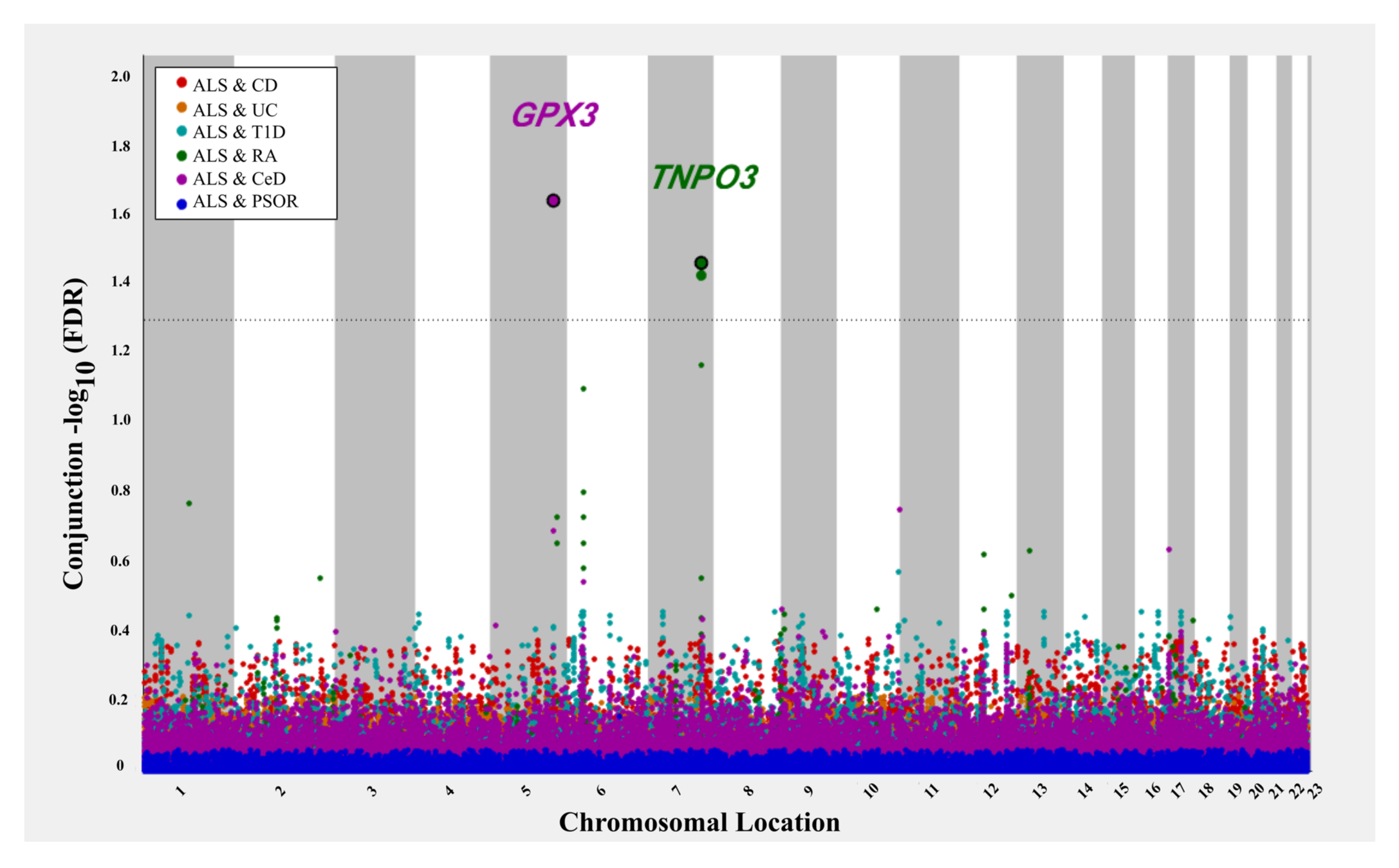

Supplement: S6 Fig — The 6 immune-mediated diseases are Crohn disease (CD; ALS|CD, red), ulcerative colitis (UC, ALS|UC, orange), type 1 diabetes (T1D, ALS|T1D, teal), rheumatoid arthritis (RA, ALS|RA, green), celiac disease (CeD, ALS|CeD, magenta), and psoriasis (PSOR, ALS|PSOR, blue). SNPs with conditional and conjunction −log10(FDR) > 1.3 (i.e., FDR < 0.05) are shown with large points. A black line around the large points indicates the most significant SNP in each linkage disequilibrium block, and this SNP was annotated with the closest gene, which is listed above the symbols in each locus. (TIFF) [file pmed.1002487.s007.tiff]

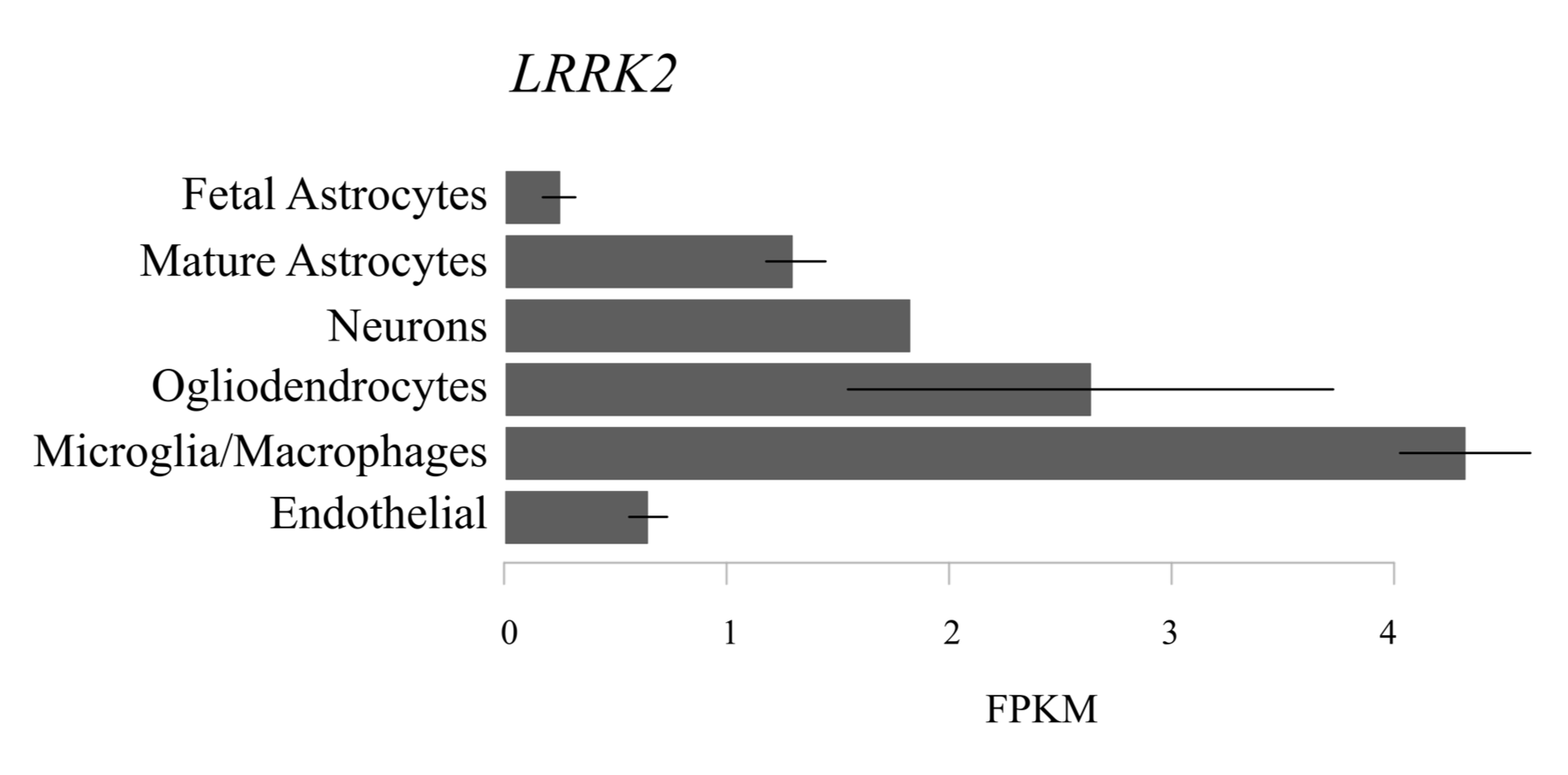

Supplement: S7 Fig — (TIFF) [file pmed.1002487.s008.tiff]

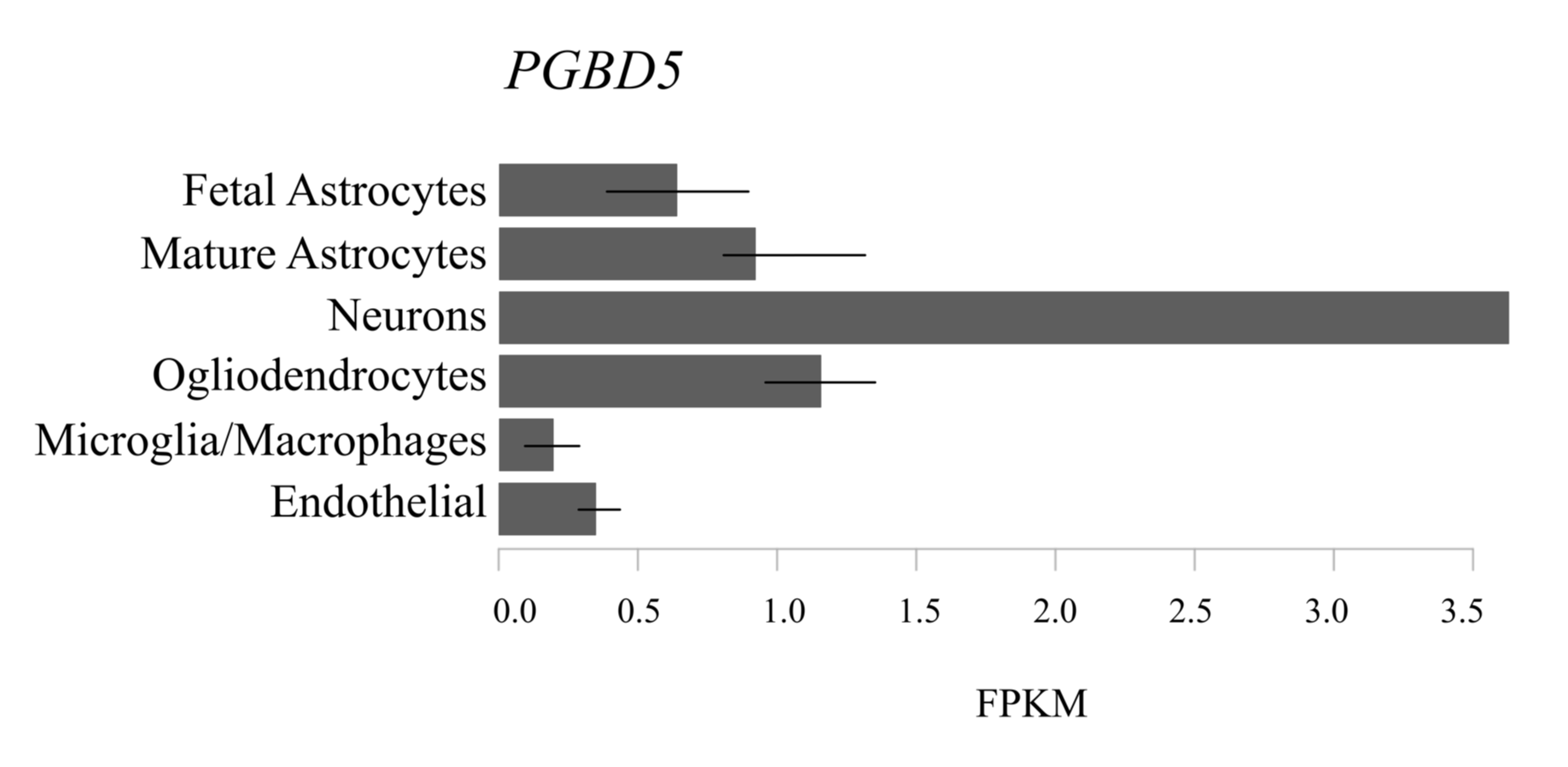

Supplement: S8 Fig — (TIFF) [file pmed.1002487.s009.tiff]

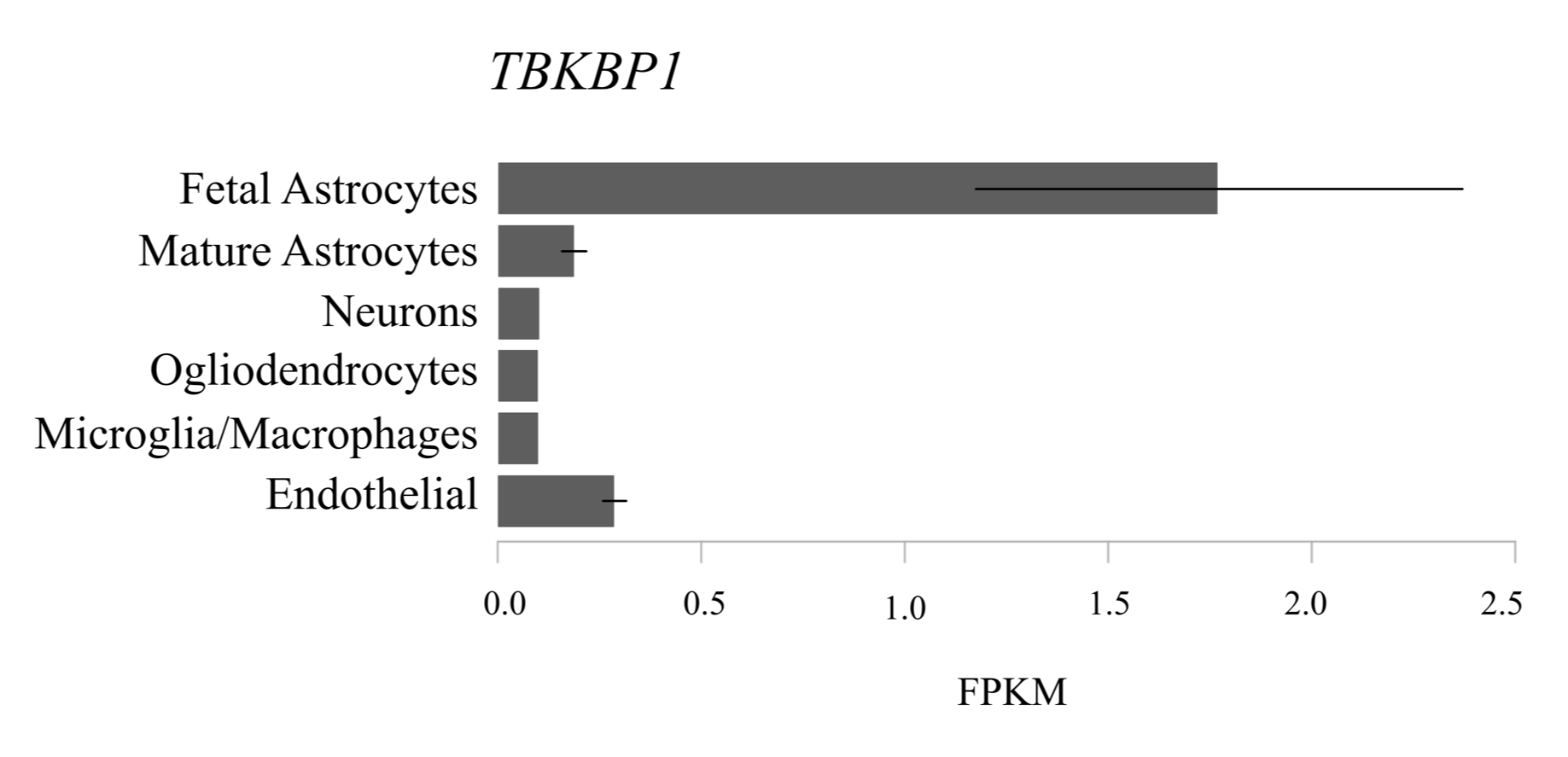

Supplement: S9 Fig — (TIFF) [file pmed.1002487.s010.tiff]
