# Supplementary material for: Immune-related genetic enrichment in frontotemporal dementia: An analysis of genome-wide association studies
Source: PLoS Med. 2018 Jan 9;15(1):e1002487. doi: 10.1371/journal.pmed.1002487 (PMC5760014; doi:10.1371/journal.pmed.1002487)
Supplement: S1 Table — (DOCX) [file pmed.1002487.s011.docx]

S1 Table. Summary-based Mendelian Randomization Results.

| **Probe**  **ID** | **SNP** | **Chr** | **SNP**  **bp** | **b**  **GWAS** | **p**  **GWAS** | **b**  **eQTL** | **se**  **eQTL** | **p**  **eQTL** | **b**  **SMR** | **p**  **SMR** | **p**  **HET** |
| --- | --- | --- | --- | --- | --- | --- | --- | --- | --- | --- | --- |
| RA | rs9268877 | 6 | 32431147 | 0.286 | 6.61E-10 | -14.77 | 1 | 2.43E-49 | -0.02 | 1.22E-08 | 1.80E-12 |
| UC | rs9268852 | 6 | 32429594 | 0.280 | 6.73E-10 | 13.72 | 1 | 7.93E-43 | 0.02 | 1.81E-08 | NA |
| CeD | rs204991 | 6 | 32161366 | 0.222 | 6.85E-05 | -146.40 | 1 | 0.0E+00 | -0.002 | 6.90E-05 | 6.62E-06 |
| PSOR | rs3094138 | 6 | 30199016 | -0.205 | 1.71E-03 | -6.80 | 1 | 1.05E-11 | 0.03 | 4.40E-03 | NA |
