# Supplementary material for: Immune-related genetic enrichment in frontotemporal dementia: An analysis of genome-wide association studies
Source: PLoS Med. 2018 Jan 9;15(1):e1002487. doi: 10.1371/journal.pmed.1002487 (PMC5760014; doi:10.1371/journal.pmed.1002487)
Supplement: S2 Table — (DOCX) [file pmed.1002487.s012.docx]

S2 Table. Overlapping loci between CBD and immune-mediated diseases at a conjunction FDR < 0.05.

| **SNP** | **Chr** | **Nearest Gene** | **Associated Phenotype** | **Associated Phenotype *p*-value** | **Min Conj FDR** | **CBD**  ***p*-value** |
| --- | --- | --- | --- | --- | --- | --- |
| rs11012 | 17 | *PLEKHM1* | T1D | 8.45E-03 | 8.45E-03 | 2.16E-01 |
| rs2074404 | 17 | *WNT3* | CeD | 3.74E-01 | 3.10E-02 | 3.39E-02 |

Abbreviations: CeD, Celiac disease; Chr, Chromosome location; CBD, Corticobasal degeneration; Min Conj FDR, minimum conjunction false discovery rate; SNP, Single-nucleotide polymorphism; T1D, Type 1 diabetes.
