# Supplementary material for: Immune-related genetic enrichment in frontotemporal dementia: An analysis of genome-wide association studies
Source: PLoS Med. 2018 Jan 9;15(1):e1002487. doi: 10.1371/journal.pmed.1002487 (PMC5760014; doi:10.1371/journal.pmed.1002487)
Supplement: S3 Table — (DOCX) [file pmed.1002487.s013.docx]

S3 Table. Overlapping loci between PSP and immune-mediated diseases at a conjunction FDR < 0.05.

| **SNP** | **Chr** | **Nearest Gene** | **Associated Phenotype** | **Associated Phenotype *p*-value** | **Min Conj FDR** | **PSP *p*-value** |
| --- | --- | --- | --- | --- | --- | --- |
| rs7642229 | 3 | *XCR1* | Ced | 1.74E-02 | 1.74E-02 | 3.92E-01 |
| rs11718668 | 3 | *TERC* | Ced | 0.030 | 2.95E-02 | 5.55E-01 |
| rs12203592 | 6 | *IRF4* | Ced | 4.17E-02 | 4.17E-02 | 6.59E-01 |
| rs1122554 | 6 | *MLN* | RA | 2.09E-02 | 2.09E-02 | 6.59E-01 |
| rs3748256 | 11 | *FAM76B* | RA | 2.09E-02 | 2.09E-02 | 3.30E-02 |
| rs11012 | 17 | *PLEKHM1* | T1D | 5.50E-03 | 5.50E-03 | 3.61E-06 |
| rs2074404 | 17 | *WNT3* | Ced | 8.83E-03 | 8.83E-03 | 3.61E-06 |

Abbreviations: CeD, Celiac disease; Chr, Chromosome location; Min Conj FDR, minimum conjunction false discovery rate; PSP, Progressive supranuclear palsy; RA, Rheumatoid arthritis; SNP, Single-nucleotide polymorphism; T1D, Type 1 diabetes.
