# Supplementary material for: Immune-related genetic enrichment in frontotemporal dementia: An analysis of genome-wide association studies
Source: PLoS Med. 2018 Jan 9;15(1):e1002487. doi: 10.1371/journal.pmed.1002487 (PMC5760014; doi:10.1371/journal.pmed.1002487)
Supplement: S4 Table — (DOCX) [file pmed.1002487.s014.docx]

S4 Table. Overlapping loci between ALS and immune-mediated diseases at a conjunction FDR < 0.05.

| **SNP** | **Chr** | **Nearest Gene** | **Associated Phenotype** | **Associated Phenotype *p*-value** | **Min Conj FDR** | **ALS *p*-value** |
| --- | --- | --- | --- | --- | --- | --- |
| rs3828599 | 5 | *GPX3* | Ced | 2.27E-02 | 2.27E-02 | 7.93E-02 |
| rs10488631 | 7 | *TNPO3* | RA | 3.42E-02 | 3.42E-02 | 2.97E-01 |

Abbreviations: ALS, Amyotrophic lateral sclerosis; CeD, Celiac disease; Chr, Chromosome location; Min Conj FDR, minimum conjunction false discovery rate; RA, Rheumatoid arthritis; SNP, Single-nucleotide polymorphism.
