# Supplementary material for: Immune-related genetic enrichment in frontotemporal dementia: An analysis of genome-wide association studies
Source: PLoS Med. 2018 Jan 9;15(1):e1002487. doi: 10.1371/journal.pmed.1002487 (PMC5760014; doi:10.1371/journal.pmed.1002487)
Supplement: S5 Table — (DOCX) [file pmed.1002487.s015.docx]

S5 Table. cis-eQTLs between FTD and immune-mediated disease shared risk SNPs and associated genes across a variety of tissues.

| **SNP** | **Gene** | **Trait** | **Gene** | ***p*-value** |
| --- | --- | --- | --- | --- |
| rs10784359 | *SLC2A13* | Whole Blood | *LRRK2* | 1.40E-07 |
|  |  | Whole Blood | *LRRK2* | 1.18E-07 |
| rs2192493 | *TWISTNB* | PBMC | *TBKBP1* | 1.29E-06 |
| rs7778450 | *TNS3* | Whole Blood | *TNS3* | 2.16E-03 |
| rs2134297 | *DCC* | PBMC | *PGBD5* | 6.04E-06 |
| rs199533 | *NSF* | Lymphoblastoid | *LOC644246* [probe 230388_s_at] | 1.90E-28 |
|  |  | Lymphoblastoid | *LRRC37A4* [probe 220220_at] | 9.80E-19 |
|  |  | Lymphoblastoid | *MAPK8IP1* [probe 213013_at] | 1.70E-12 |
|  |  | PBMC | *NSF* [probe 3724236] | 1.73E-08 |
|  |  | Frontal Cortex | *LRRC37A* | 2.84E-13 |
|  |  | Cerebellum | *MAPK8IP1* | 2.99E-12 |
|  |  | Cerebellum | *MAPT* | 4.77E-10 |
|  |  | Frontal Cortex | *MAPT* | 6.02E-09 |
|  |  | Whole Blood | *NSF* | 2.80E-07 |
|  |  | Whole Blood | *KANSL1-AS1* | 8.37E-69 |
|  |  | Whole Blood | *LRRC37A* | 2.84E-10 |
|  |  | Whole Blood | *LRRC37A2* | 5.53E-27 |
|  |  | Whole Blood | *RP11-259G18.2* | 1.01E-68 |
|  |  | Whole Blood | *RP11-259G18.3* | 4.56E-60 |
|  |  | Whole Blood | *NSF* | 2.63E-11 |
|  |  | Cerebellum | *KANSL1-AS1* | 1.98E-20 |
|  |  | Cerebellum | *LRRC37A* | 4.13E-13 |
|  |  | Cerebellum | *LRRC37A2* | 4.16E-19 |
|  |  | Cerebellum | *MAPT* | 6.31E-12 |
|  |  | Cerebellum | *MAPT-AS1* | 2.56E-08 |
|  |  | Brain Cortex | *KANSL1-AS1* | 1.32E-18 |
|  |  | Brain Cortex | *LRRC37A2* | 4.26E-14 |
|  |  | Brain Frontal Cortex BA9 | *KANSL1-AS1* | 4.64E-21 |
|  |  | Brain Frontal Cortex BA9 | *LRRC37A2* | 3.30E-16 |
|  |  | Brain Frontal Cortex BA9 | *MAPT* | 8.24E-08 |
| rs3135353 | *HLA-DRA* | Lymphoblastoid | *HLA-DQA1* [probe 203290_at] | 1.30E-12 |
|  |  | Lymphoblastoid | *HLA-DRB1* [probe 204670_x_at] | 4.00E-15 |
|  |  | Lymphoblastoid | *HLA-DRB1* [probe 209312_x_at] | 1.00E-08 |
|  |  | Lymphoblastoid | *HLA-DRB1* [probe 221491_x_at] | 1.90E-17 |
|  |  | Lymphoblastoid | *HLA-DRB2* [probe 204670_x_at] | 4.00E-15 |
|  |  | Lymphoblastoid | *HLA-DRB2* [probe 209312_x_at] | 1.00E-08 |
|  |  | Lymphoblastoid | *HLA-DRB2* [probe 221491_x_at] | 1.90E-17 |
|  |  | Lymphoblastoid | *HLA-DRB3* [probe 204670_x_at] | 4.00E-15 |
|  |  | Lymphoblastoid | *HLA-DRB3* [probe 209312_x_at] | 1.00E-08 |
|  |  | Lymphoblastoid | *HLA-DRB3* [probe 221491_x_at] | 1.90E-17 |
|  |  | Lymphoblastoid | *HLA-DRB4* [probe 204670_x_at] | 4.00E-15 |
|  |  | Lymphoblastoid | *HLA-DRB4* [probe 209312_x_at] | 1.00E-08 |
|  |  | Lymphoblastoid | *HLA-DRB4* [probe 209728_at] | 8.60E-09 |
|  |  | Lymphoblastoid | *HLA-DRB4* [probe 221491_x_at] | 1.90E-17 |
|  |  | Lymphoblastoid | *HLA-DRB5* [probe 204670_x_at] | 4.00E-15 |
|  |  | Lymphoblastoid | *HLA-DRB5* [probe 209312_x_at] | 1.00E-08 |
|  |  | Lymphoblastoid | *HLA-DRB5* [probe 221491_x_at] | 1.90E-17 |
|  |  | Lymphoblastoid | *RNASE2* [probe 204670_x_at] | 4.00E-15 |
|  |  | Lymphoblastoid | *RNASE2* [probe 209312_x_at] | 1.00E-08 |
|  |  | Lymphoblastoid | *RNASE2* [probe 221491_x_at] | 1.90E-17 |
|  |  | Lymphoblastoid | *ZNF749* [probe 204670_x_at] | 4.00E-15 |
|  |  | Lymphoblastoid | *ZNF749* [probe 209312_x_at] | 1.00E-08 |
|  |  | Lymphoblastoid | *ZNF749* [probe 221491_x_at] | 1.90E-17 |
|  |  | Whole Blood | *HLA-DRA* | 6.90E-04 |
|  |  | Whole Blood | *HLA-DRB5/HLA* | 6.80E-54 |
|  |  | Whole Blood | *RNF5/AGER* | 1.60E-05 |
|  |  | Whole Blood | *XRCC6* | 1.70E-09 |
|  |  | Cerebellum | *C4A* | 3.14E-07 |
|  |  | Cerebellum | *HLA-DMA* | 4.82E-07 |
|  |  | Whole Blood | *C4A* | 2.43E-11 |
|  |  | Whole Blood | *C4B* | 6.01E-12 |
|  |  | Whole Blood | *CYP21A1P* | 2.93E-07 |
|  |  | Whole Blood | *HLA-DQA2* | 8.68E-09 |
|  |  | Whole Blood | *HLA-DRB6* | 3.08E-07 |
|  |  | Whole Blood | *HLA-DRA* | 7.38E-20 |
| rs875142 | *PAQR8* | Whole Blood | *PAQR8* | 4.65E-58 |
|  |  | Whole Blood | *TRAM2* | 0.0032170 |
| rs204989 | *GPSM3* | PMBC | *BTN3A2* | 3.09E-07 |
|  |  | PMBC | *HCG27* | 4.76E-15 |
|  |  | PMBC | *HLA-C* | 9.64E-12 |
|  |  | PMBC | *HLA-DPB1* | 9.63E-19 |
|  |  | PMBC | *HLA-DQA1* | 4.59E-08 |
|  |  | PMBC | *HLA-DQB1* | 1.78E-28 |
|  |  | PMBC | *HLA-DRB1* | 4.64E-25 |
|  |  | PMBC | *HLA-DRB3* | 1.98E-08 |
|  |  | PMBC | *HLA-DRB4* | 7.00E-40 |
|  |  | PMBC | *HLA-DRB5* | 1.14E-14 |
|  |  | PMBC | *RFPL3* | 7.83E-07 |
|  |  | PMBC | *VARS2* | 9.77E-08 |
|  |  | Whole Blood | *C4A* | 4.14E-07 |
|  |  | Whole Blood | *C4B* | 1.03E-11 |
|  |  | Whole Blood | *CYP21A2* | 1.60E-06 |
|  |  | Whole Blood | *AGER* | 9.07E-04 |
|  |  | Whole Blood | *HLA-DRA* | 2.14E-07 |
| rs204991 | *GPSM3* | Lymphoblastoid | *HLA-DQB1* [probe 209480_at] | 3.20E-09 |
|  |  | Lymphoblastoid | *HLA-DQB1* [probe 211654_x_at] | 3.10E-10 |
|  |  | Lymphoblastoid | *HLA-DQB1* [probe 212998_x_at] | 9.90E-08 |
|  |  | Lymphoblastoid | *HLA-DQB1* [probe 212999_x_at] | 8.20E-11 |
|  |  | PBMC | *BTN3A2* | 3.38E-07 |
|  |  | PBMC | *HCG27* | 7.13E-15 |
|  |  | PBMC | *HLA-C* | 8.86E-12 |
|  |  | PBMC | *HLA-DPB1* | 9.28E-19 |
|  |  | PBMC | *HLA-DQA1* | 4.68E-08 |
|  |  | PBMC | *HLA-DQB1* | 1.76E-28 |
|  |  | PBMC | *HLA-DRB1* | 1.08E-24 |
|  |  | PBMC | *HLA-DRB3* | 2.93E-08 |
|  |  | PBMC | *HLA-DRB4* | 5.68E-40 |
|  |  | PBMC | *HLA-DRB5* | 2.79E-14 |
|  |  | PBMC | *RFPL3* | 8.84E-07 |
|  |  | PBMC | *VARS2* | 1.38E-07 |
|  |  | Whole Blood | *AGER* | 2.70E-05 |
|  |  | Whole Blood | *HLA-DRA* | 6.50E-21 |
|  |  | Whole Blood | *C4A* | 3.89E-07 |
|  |  | Whole Blood | *C4B* | 1.04E-11 |
|  |  | Whole Blood | *CYP21A2* | 1.54E-06 |
|  |  | Whole Blood | *AGER* | 9.65E-04 |
| rs9268852 | *HLA-DRA* | Brain Anterior cingulate cortex BA24 | *HLA-DRB1* | 8.77E-08 |
|  |  | Cerebellum | *HLA-DQA2* | 1.32E-06 |
|  |  | Cerebellum | *HLA-DRB1* | 5.23E-08 |
|  |  | Whole Blood | *HLA-DQA1* | 2.84E-08 |
|  |  | Whole Blood | *HLA-DQA2* | 1.94E-23 |
|  |  | Whole Blood | *HLA-DQB1* | 1.23E-12 |
|  |  | Whole Blood | *HLA-DQB1-AS1* | 2.50E-10 |
|  |  | Whole Blood | *HLA-DQB2* | 6.54E-08 |
|  |  | Whole Blood | *HLA-DRB1* | 2.91E-21 |
|  |  | Whole Blood | *HLA-DRB6* | 7.54E-10 |
|  |  | Lymphoblastoid | *HLA-DQA1* | 1.02E-07 |
|  |  | Lymphoblastoid | *HLA-DQA1* | 1.03E-06 |
|  |  | Lymphoblastoid | *HLA-DQA1* | 1.10E-07 |
|  |  | Lymphoblastoid | *HLA-DQA1* | 1.13E-06 |
|  |  | Lymphoblastoid | *HLA-DQA1* | 7.76E-06 |
|  |  | Lymphoblastoid | *HLA-DQB1* | 1.33E-10 |
|  |  | Lymphoblastoid | *HLA-DQB1* | 5.80E-10 |
|  |  | Lymphoblastoid | *HLA-DQB1* | 6.30E-07 |
|  |  | Lymphoblastoid | *HLA-DQB1* | 8.40E-08 |
| rs9268877 | *HLA-DRA* | Lymphoblastoid | *HLA-DQA1* | 1.35E-17 |
|  |  | Lymphoblastoid | *HLA-DQA2* | 2.34E-11 |
|  |  | Lymphoblastoid | *Spc25* | 1.18E-08 |
|  |  | PBMC | *AOAH* | 1.41E-21 |
|  |  | PBMC | *HCG27* | 1.52E-09 |
|  |  | PBMC | *HLA-DMA* | 2.92E-06 |
|  |  | PBMC | *HLA-DOB* | 4.88E-07 |
|  |  | PBMC | *HLA-DPB1* | 1.85E-07 |
|  |  | PBMC | *HLA-DQA1* | 7.56E-18 |
|  |  | PBMC | *HLA-DQB1* | 1.53E-73 |
|  |  | PBMC | *HLA-DRB1* | 1.39E-70 |
|  |  | PBMC | *HLA-DRB3* | 7.45E-22 |
|  |  | PBMC | *HLA-DRB4* | 7.70E-27 |
|  |  | PBMC | *HLA-DRB5* | 2.30E-74 |
|  |  | PBMC | *HLA-DRB6* | 5.47E-12 |
|  |  | PBMC | *SSRP1* | 6.69E-07 |
|  |  | Whole Blood | *AOAH* | 6.90E-09 |
|  |  | Whole Blood | *HLA-DRB5* | 2.40E-15 |
|  |  | Whole Blood | *HLA-DRB5/HLA-DRB1* | 5.50E-18 |
|  |  | Whole Blood | *KRT18* | 5.30E-06 |
|  |  | Whole Blood | *LIMS1* | 4.40E-12 |
|  |  | Whole Blood | *TRBV18* | 5.10E-09 |
|  |  | Whole Blood | *HLA-DQB1* (probeID ILMN_1661266) | 3.24E-45 |
|  |  | Whole Blood | *HLA-DRB1* (probeID ILMN_1715169) | 1.32E-61 |
|  |  | Whole Blood | *HLA-DRB5* (probeID ILMN_1697499) | 1.10E-34 |
|  |  | Brain Anterior cingulate cortex BA24 | *HLA-DRB1* | 8.77E-08 |
|  |  | Cerebellum | *HLA-DQA2* | 1.32E-06 |
|  |  | Cerebellum | *HLA-DRB1* | 5.23E-08 |
|  |  | Whole Blood | *HLA-DQA1* | 2.84E-08 |
|  |  | Whole Blood | *HLA-DQA2* | 1.94E-23 |
|  |  | Whole Blood | *HLA-DQB1* | 1.23E-12 |
|  |  | Whole Blood | *HLA-DQB1-AS1* | 2.50E-10 |
|  |  | Whole Blood | *HLA-DQB2* | 6.54E-08 |
|  |  | Whole Blood | *HLA-DRB1* | 2.91E-21 |
|  |  | Whole Blood | *HLA-DRB6* | 7.54E-10 |
|  |  | Lymphoblastoid | *HLA-DQA1* | 2.82E-14 |
|  |  | Whole Blood | *HLA-DRA* | 4.04E-05 |
| rs3117097 | *BTNL2* | Whole Blood | *C4A* | 2.44E-07 |
|  |  | Whole Blood | *C4B* | 1.97E-10 |
|  |  | Whole Blood | *HLA-DRB6* | 1.91E-07 |
|  |  | Whole Blood | *HLA-DRA* | 1.85E-19 |
| rs3094138 | *TRIM26* | PBMC | *ABCF1* | 5.10E-10 |
|  |  | PBMC | *HLA-A* | 2.38E-09 |
|  |  | PBMC | *HLA-A29.1* | 1.45E-25 |
|  |  | PBMC | *HLA-DRB1* | 2.13E-07 |
|  |  | PBMC | *HLA-DRB5* | 5.52E-11 |
|  |  | PBMC | *HLA-F* | 8.52E-13 |
|  |  | PBMC | *HLA-H* | 8.30E-21 |
|  |  | PBMC | *KIT* | 1.79E-06 |
|  |  | PBMC | *MDC1* | 5.13E-08 |
|  |  | PBMC | *PPP1R11* | 9.86E-06 |
|  |  | Whole Blood | *TRIM10* | 3.20E-04 |
|  |  | Cerebellum | *HLA-K* | 2.46E-06 |
|  |  | Brain_Cortex | *HCG4P5* | 2.55E-07 |
|  |  | Brain_Cortex | *HLA-K* | 1.72E-07 |
|  |  | Whole Blood | *HCG4P5* | 2.55E-20 |
|  |  | Whole Blood | *HLA-A* | 3.18E-13 |
|  |  | Whole Blood | *HLA-J* | 5.27E-09 |
|  |  | Whole Blood | *MICD* | 2.75E-06 |
|  |  | Whole Blood | *PPP1R11* | 1.53E-76 |
| rs9261536 | *TRIM15* | PBMC | *ABCF1* | 3.45E-10 |
|  |  | PBMC | *HCG27* | 2.05E-09 |
|  |  | PBMC | *HLA-A* | 2.59E-08 |
|  |  | PBMC | *HLA-A29.1* | 6.95E-24 |
|  |  | PBMC | *HLA-DRB1* | 3.07E-07 |
|  |  | PBMC | *HLA-DRB5* | 2.48E-11 |
|  |  | PBMC | *HLA-F* | 1.18E-11 |
|  |  | PBMC | *HLA-H* | 8.60E-21 |
|  |  | PBMC | *KIT* | 2.01E-09 |
|  |  | PBMC | *MDC1* | 6.05E-09 |
|  |  | PBMC | *NDUFS1* | 5.96E-08 |
|  |  | PBMC | *PPP1R11* | 5.88E-09 |
|  |  | PBMC | *VARS2* | 6.97E-07 |
|  |  | Whole Blood | *HLA-H (probeID ILMN_1666078)* | 5.15E-17 |
|  |  | Cerebellar Hemisphere | *HCG4P5* | 3.76E-07 |
|  |  | Cerebellar Hemisphere | *HLA-A* | 4.09E-06 |
|  |  | Cerebellum | *HCG4P5* | 4.33E-08 |
|  |  | Cerebellum | *HLA-A* | 4.46E-06 |
|  |  | Cerebellum | *HLA-H* | 6.73E-07 |
|  |  | Cerebellum | *HLA-K* | 5.46E-07 |
|  |  | Brain_Cortex | *HCG4P5* | 1.08E-07 |
|  |  | Brain_Cortex | *HLA-K* | 1.73E-08 |
|  |  | Brain Frontal Cortex BA9 | *HCG4P5* | 3.51E-07 |
|  |  | Brain Frontal Cortex BA9 | *HLA-K* | 3.01E-07 |
|  |  | Whole Blood | *DDX39BP2* | 1.10E-05 |
|  |  | Whole Blood | *HCG4P5* | 9.42E-26 |
|  |  | Whole Blood | *HLA-A* | 3.15E-17 |
|  |  | Whole Blood | *HLA-H* | 1.09E-05 |
|  |  | Whole Blood | *HLA-J* | 2.72E-11 |
|  |  | Whole Blood | *MICD* | 7.80E-07 |
|  |  | Whole Blood | *PPP1R11* | 2.98E-110 |
| rs2269423 | *AGPAT1* | Lymphoblastoid | *HLA-DQA1* [probe 213831_at] | 4.00E-13 |
|  |  | Lymphoblastoid | *HLA-DQA1* [probe 236203_at] | 4.10E-16 |
|  |  | Lymphoblastoid | *HLA-DQA1* probe[213831_at] | 3.74E-12 |
|  |  | Lymphoblastoid | *HLA-DQA1* probe[236203_at] | 4.23E-15 |
|  |  | Lymphoblastoid | *HLA-DQB1* [probe 209480_at] | 5.40E-19 |
|  |  | Lymphoblastoid | *HLA-DQB1* [probe 209823_x_at] | 2.60E-10 |
|  |  | Lymphoblastoid | *HLA-DQB1* [probe 211654_x_at] | 8.90E-12 |
|  |  | Lymphoblastoid | *HLA-DQB1* [probe 212999_x_at] | 9.70E-18 |
|  |  | Lymphoblastoid | *HLA-DQB1* probe[209480_at] | 6.07E-18 |
|  |  | Lymphoblastoid | *HLA-DQB1* probe[209823_x_at] | 2.15E-09 |
|  |  | Lymphoblastoid | *HLA-DQB1* probe[211654_x_at] | 7.78E-11 |
|  |  | Lymphoblastoid | *HLA-DQB1* probe[212999_x_at] | 1.06E-16 |
|  |  | Lymphoblastoid | *HLA-DRB1* [probe 204670_x_at] | 3.20E-08 |
|  |  | Lymphoblastoid | *HLA-DRB1* [probe 209312_x_at] | 1.60E-08 |
|  |  | Lymphoblastoid | *HLA-DRB1* probe[204670_x_at] | 2.29E-07 |
|  |  | Lymphoblastoid | *HLA-DRB1* probe[209312_x_at] | 1.17E-07 |
|  |  | Lymphoblastoid | *HLA-DRB1* probe[238900_at] | 7.55E-10 |
|  |  | Lymphoblastoid | *HLA-DRB2* [probe 204670_x_at] | 3.20E-08 |
|  |  | Lymphoblastoid | *HLA-DRB2* [probe 209312_x_at] | 1.60E-08 |
|  |  | Lymphoblastoid | *HLA-DRB3* [probe 204670_x_at] | 3.20E-08 |
|  |  | Lymphoblastoid | *HLA-DRB3* [probe 209312_x_at] | 1.60E-08 |
|  |  | Lymphoblastoid | *HLA-DRB3* probe[238900_at] | 7.55E-10 |
|  |  | Lymphoblastoid | *HLA-DRB4* [probe 204670_x_at] | 3.20E-08 |
|  |  | Lymphoblastoid | *HLA-DRB4* [probe 209312_x_at] | 1.60E-08 |
|  |  | Lymphoblastoid | *HLA-DRB5* [probe 204670_x_at] | 3.20E-08 |
|  |  | Lymphoblastoid | *HLA-DRB5* [probe 209312_x_at] | 1.60E-08 |
|  |  | Lymphoblastoid | *RNASE2* [probe 204670_x_at] | 3.20E-08 |
|  |  | Lymphoblastoid | *RNASE2* [probe 209312_x_at] | 1.60E-08 |
|  |  | Lymphoblastoid | *ZNF749* [probe 204670_x_at] | 3.20E-08 |
|  |  | Lymphoblastoid | *ZNF749* [probe 209312_x_at] | 1.60E-08 |
|  |  | Whole Blood | *HLA-DQA1* | 1.73E-09 |
|  |  | Whole Blood | *HLA-DQA2* | 1.10E-08 |
|  |  | Whole Blood | *HLA-DQB1* | 2.10E-10 |
|  |  | Whole Blood | *HLA-DQB1-AS1* | 2.25E-08 |
|  |  | Whole Blood | *HLA-DQB2* | 1.75E-10 |
|  |  | Whole Blood | *HLA-DRB1* | 8.59E-08 |
|  |  | Lymphoblastoid | *HLA-DQA1* | 2.25E-07 |
|  |  | Lymphoblastoid | *HLA-DQA1* | 4.21E-07 |
|  |  | Lymphoblastoid | *HLA-DQA1* | 7.20E-06 |
|  |  | Lymphoblastoid | *HLA-DQA1* | 7.62E-07 |
|  |  | Lymphoblastoid | *HLA-DQA1* | 8.21E-07 |
|  |  | Lymphoblastoid | *HLA-DQB1* | 2.44E-07 |
|  |  | Lymphoblastoid | *HLA-DQB1* | 6.29E-06 |
|  |  | Lymphoblastoid | *HLA-DQB1* | 7.69E-07 |
|  |  | Lymphoblastoid | *HLA-DQB1* | 8.88E-06 |
|  |  | Whole Blood | *GPSM3\|NOTCH4* | 5.95E-05 |
| rs17572851 | *MAPT* | Cortex (normal controls) | *MAPT* probe [GI_8400714-A] | 3.61E-12 |
|  |  | Frontal cortex | *LRRC37A* | 6.56E-13 |
|  |  | Cerebellum | *MAPK8IP1* | 2.56E-10 |
|  |  | Cerebellum | *MAPT* | 1.06E-09 |
|  |  | PBMC | *FALZ* | 1.81E-75 |
|  |  | PBMC | *LRRC37A4* | 5E-324 |
|  |  | PBMC | *MAPK8IP1* | 5E-324 |
|  |  | Whole Blood | *KIAA1267* | 3.77E-33 |
| rs3129890 | *HLA-DRA* | Whole Blood | *AOAH* | 1.20E-09 |
|  |  | Whole Blood | *HLA-DRA* | 1.40E-05 |
|  |  | Whole Blood | *HLA-DRB5/HLA-DRB1* | 4.80E-37 |
|  |  | Whole Blood | *HLA-DRB5* | 8.04E-07 |
|  |  | Whole Blood | *HLA-DRB6* | 1.70E-11 |
|  |  | Whole Blood | *HLA-DRA* | 3.76E-20 |
| rs6457590 | *HLA-DRA* | Whole Blood | *HLA-DRA* | 5.19E-39 |
| rs10484561 | *HLA-DQB1* | Lymphoblastoid | *HLA-DQA1* [probe 236203_at] | 1.80E-17 |
|  |  | Lymphoblastoid | *HLA-DQB1* [probe 209480_at] | 1.20E-07 |
|  |  | Lymphoblastoid | *HLA-DQB1* [probe 212999_x_at] | 4.80E-11 |
|  |  | PBMC | *HLA-DQA2* [probe 2903266] | 4.03E-10 |
|  |  | PBMC | *HLA-DQA2* [probe 2903258] | 5.83E-07 |
|  |  | Whole Blood | *HLA-DRB5* | 7.50E-157 |
|  |  | Whole Blood | *LIMS1* | 1.30E-14 |
|  |  | Whole Blood | *TAP2* | 2.10E-14 |
|  |  | Whole Blood | *HLA-DQB1* (probeID ILMN_1440296) | 7.53E-10 |
|  |  | Whole Blood | *HLA-DQA1* | 2.57E-07 |
|  |  | Whole Blood | *HLA-DRB6* | 3.77E-10 |
|  |  | Whole Blood | *HLA-DMB* | 3.18E-3 |
|  |  | Whole Blood | *PSMB9* | 5.51E-06 |
|  |  | Whole Blood | *TAP2* | 3.89E-60 |
|  |  | Whole Blood | *TAP2* | 4.44E-04 |

Abbreviation: PBMC, peripheral blood mononuclear cells
