# Supplementary material for: Immune-related genetic enrichment in frontotemporal dementia: An analysis of genome-wide association studies
Source: PLoS Med. 2018 Jan 9;15(1):e1002487. doi: 10.1371/journal.pmed.1002487 (PMC5760014; doi:10.1371/journal.pmed.1002487)
Supplement: S6 Table — (DOCX) [file pmed.1002487.s016.docx]

S6 Table. Physical interaction and gene co-expression networks for the pleiotropic genes with significant *cis*-eQTLs.

| Entity 1 | Entity 2 | Weight | Network group |
| --- | --- | --- | --- |
| *AOAH* | *HLA-F* | 0.008 | Co-expression |
| *AOAH* | *HLA-C* | 0.004 | Co-expression |
| *AOAH* | *HLA-F* | 0.010 | Co-expression |
| *AZI2* | *TBKBP1* | 0.500 | Shared protein domains |
| *AZI2* | *TBKBP1* | 0.500 | Shared protein domains |
| *B2M* | *HLA-C* | 0.039 | Co-expression |
| *B2M* | *HLA-F* | 0.020 | Co-expression |
| *B2M* | *HLA-A* | 0.024 | Co-expression |
| *B2M* | *HLA-G* | 0.034 | Co-expression |
| *B2M* | *HLA-B* | 0.027 | Co-expression |
| *B2M* | *HLA-C* | 0.029 | Co-expression |
| *B2M* | *HLA-A* | 0.025 | Co-expression |
| *B2M* | *HLA-DRA* | 0.009 | Co-expression |
| *B2M* | *HLA-B* | 0.023 | Co-expression |
| *B2M* | *HLA-A* | 0.013 | Co-expression |
| *B2M* | *HLA-C* | 0.010 | Co-expression |
| *B2M* | *HLA-F* | 0.010 | Co-expression |
| *B2M* | *HLA-A* | 0.012 | Co-expression |
| *B2M* | *HLA-DRA* | 0.010 | Co-expression |
| *B2M* | *HLA-C* | 0.015 | Co-expression |
| *B2M* | *HLA-A* | 0.010 | Co-expression |
| *B2M* | *HLA-B* | 0.010 | Co-expression |
| *B2M* | *HLA-A* | 0.020 | Co-expression |
| *B2M* | *HLA-DRA* | 0.017 | Co-expression |
| *B2M* | *HLA-C* | 0.027 | Co-expression |
| *B2M* | *HLA-F* | 0.023 | Co-expression |
| *B2M* | *HLA-A* | 0.025 | Co-expression |
| *B2M* | *HLA-G* | 0.029 | Co-expression |
| *B2M* | *HLA-B* | 0.023 | Co-expression |
| *B2M* | *HLA-C* | 0.024 | Co-expression |
| *B2M* | *HLA-A* | 0.024 | Co-expression |
| *B2M* | *HLA-G* | 0.021 | Co-expression |
| *B2M* | *HLA-B* | 0.021 | Co-expression |
| *B2M* | *HLA-F* | 0.011 | Co-expression |
| *B2M* | *HLA-A* | 0.011 | Co-expression |
| *B2M* | *HLA-DRA* | 0.009 | Co-expression |
| *B2M* | *HLA-G* | 0.009 | Co-expression |
| *B2M* | *HLA-F* | 0.018 | Co-expression |
| *B2M* | *AOAH* | 0.016 | Co-expression |
| *B2M* | *HLA-A* | 0.016 | Co-expression |
| *B2M* | *HLA-DRA* | 0.018 | Co-expression |
| *B2M* | *HLA-G* | 0.024 | Co-expression |
| *B2M* | *HLA-C* | 0.009 | Co-expression |
| *B2M* | *HLA-F* | 0.007 | Co-expression |
| *B2M* | *HLA-A* | 0.007 | Co-expression |
| *B2M* | *HLA-DRA* | 0.014 | Co-expression |
| *B2M* | *HLA-G* | 0.010 | Co-expression |
| *B2M* | *HLA-B* | 0.008 | Co-expression |
| *B2M* | *HLA-A* | 0.029 | Co-expression |
| *B2M* | *HLA-DRA* | 0.031 | Co-expression |
| *B2M* | *HLA-G* | 0.033 | Co-expression |
| *B2M* | *HLA-C* | 0.013 | Co-expression |
| *B2M* | *HLA-A* | 0.014 | Co-expression |
| *B2M* | *HLA-DRA* | 0.004 | Co-expression |
| *B2M* | *HLA-C* | 0.027 | Co-expression |
| *B2M* | *HLA-A* | 0.020 | Co-expression |
| *B2M* | *HLA-A* | 0.014 | Co-expression |
| *B2M* | *HLA-C* | 0.014 | Co-expression |
| *B2M* | *HLA-F* | 0.010 | Co-expression |
| *B2M* | *HLA-A* | 0.013 | Co-expression |
| *B2M* | *HLA-G* | 0.011 | Co-expression |
| *B2M* | *HLA-B* | 0.011 | Co-expression |
| *B2M* | *HLA-F* | 0.023 | Co-expression |
| *B2M* | *HLA-A* | 0.008 | Co-expression |
| *B2M* | *HLA-DRA* | 0.009 | Co-expression |
| *B2M* | *HLA-B* | 0.018 | Co-expression |
| *B2M* | *HLA-F* | 0.006 | Co-localization |
| *B2M* | *HLA-DRA* | 0.005 | Co-localization |
| *B2M* | *HLA-G* | 0.009 | Co-localization |
| *B2M* | *HLA-C* | 0.086 | Physical Interactions |
| *B2M* | *HLA-F* | 0.286 | Physical Interactions |
| *B2M* | *HLA-B* | 0.090 | Physical Interactions |
| *B2M* | *HLA-C* | 0.027 | Predicted |
| *B2M* | *HLA-A* | 0.028 | Predicted |
| *B2M* | *HLA-DRA* | 0.050 | Predicted |
| *B2M* | *HLA-C* | 0.015 | Shared protein domains |
| *B2M* | *HLA-F* | 0.017 | Shared protein domains |
| *B2M* | *HLA-A* | 0.017 | Shared protein domains |
| *B2M* | *HLA-DRA* | 0.018 | Shared protein domains |
| *B2M* | *HLA-G* | 0.017 | Shared protein domains |
| *B2M* | *HLA-B* | 0.017 | Shared protein domains |
| *B2M* | *HLA-C* | 0.019 | Shared protein domains |
| *B2M* | *HLA-F* | 0.020 | Shared protein domains |
| *B2M* | *HLA-A* | 0.019 | Shared protein domains |
| *B2M* | *HLA-DRA* | 0.023 | Shared protein domains |
| *B2M* | *HLA-G* | 0.020 | Shared protein domains |
| *B2M* | *HLA-B* | 0.019 | Shared protein domains |
| *CD74* | *HLA-C* | 0.011 | Co-expression |
| *CD74* | *HLA-F* | 0.010 | Co-expression |
| *CD74* | *HLA-A* | 0.011 | Co-expression |
| *CD74* | *HLA-DRA* | 0.007 | Co-expression |
| *CD74* | *HLA-G* | 0.010 | Co-expression |
| *CD74* | *HLA-B* | 0.011 | Co-expression |
| *CD74* | *HLA-E* | 0.010 | Co-expression |
| *CD74* | *HLA-DRB1* | 0.007 | Co-expression |
| *CD74* | *HLA-DPB1* | 0.009 | Co-expression |
| *CD74* | *HLA-DMA* | 0.007 | Co-expression |
| *CD74* | *HLA-DQB1* | 0.010 | Co-expression |
| *CD74* | *HLA-C* | 0.007 | Co-expression |
| *CD74* | *HLA-F* | 0.005 | Co-expression |
| *CD74* | *AOAH* | 0.004 | Co-expression |
| *CD74* | *HLA-A* | 0.006 | Co-expression |
| *CD74* | *HLA-DRA* | 0.004 | Co-expression |
| *CD74* | *HLA-G* | 0.006 | Co-expression |
| *CD74* | *HLA-B* | 0.007 | Co-expression |
| *CD74* | *B2M* | 0.007 | Co-expression |
| *CD74* | *HLA-E* | 0.004 | Co-expression |
| *CD74* | *TAP1* | 0.006 | Co-expression |
| *CD74* | *HLA-DRB1* | 0.004 | Co-expression |
| *CD74* | *HLA-DPB1* | 0.004 | Co-expression |
| *CD74* | *HLA-DMA* | 0.003 | Co-expression |
| *CD74* | *HLA-DQB1* | 0.008 | Co-expression |
| *CD74* | *HLA-DRA* | 0.020 | Co-expression |
| *CD74* | *HLA-DRB1* | 0.022 | Co-expression |
| *CD74* | *HLA-DPB1* | 0.023 | Co-expression |
| *CD74* | *HLA-DMA* | 0.023 | Co-expression |
| *CD74* | *HLA-DRA* | 0.032 | Co-expression |
| *CD74* | *HLA-DRB1* | 0.020 | Co-expression |
| *CD74* | *TBKBP1* | 0.013 | Co-expression |
| *CD74* | *HLA-A* | 0.009 | Co-expression |
| *CD74* | *HLA-F* | 0.018 | Co-expression |
| *CD74* | *HLA-A* | 0.020 | Co-expression |
| *CD74* | *HLA-DRA* | 0.021 | Co-expression |
| *CD74* | *B2M* | 0.015 | Co-expression |
| *CD74* | *TAP1* | 0.016 | Co-expression |
| *CD74* | *HLA-DRB1* | 0.021 | Co-expression |
| *CD74* | *HLA-DPB1* | 0.020 | Co-expression |
| *CD74* | *HLA-DMA* | 0.019 | Co-expression |
| *CD74* | *HLA-DQB1* | 0.020 | Co-expression |
| *CD74* | *HLA-DRA* | 0.022 | Co-expression |
| *CD74* | *HLA-DRB1* | 0.015 | Co-expression |
| *CD74* | *HLA-DPB1* | 0.011 | Co-expression |
| *CD74* | *HLA-DMA* | 0.015 | Co-expression |
| *CD74* | *HLA-DQB1* | 0.021 | Co-expression |
| *CD74* | *HLA-C* | 0.011 | Co-expression |
| *CD74* | *HLA-DRA* | 0.023 | Co-expression |
| *CD74* | *HLA-B* | 0.012 | Co-expression |
| *CD74* | *HLA-DRB1* | 0.021 | Co-expression |
| *CD74* | *HLA-DPB1* | 0.022 | Co-expression |
| *CD74* | *HLA-DMA* | 0.017 | Co-expression |
| *CD74* | *HLA-DQB1* | 0.024 | Co-expression |
| *CD74* | *HLA-F* | 0.009 | Co-expression |
| *CD74* | *AOAH* | 0.010 | Co-expression |
| *CD74* | *HLA-A* | 0.008 | Co-expression |
| *CD74* | *HLA-DRA* | 0.009 | Co-expression |
| *CD74* | *HLA-G* | 0.007 | Co-expression |
| *CD74* | *B2M* | 0.010 | Co-expression |
| *CD74* | *HLA-E* | 0.006 | Co-expression |
| *CD74* | *HLA-DPB1* | 0.012 | Co-expression |
| *CD74* | *HLA-DMA* | 0.009 | Co-expression |
| *CD74* | *HLA-DQB1* | 0.012 | Co-expression |
| *CD74* | *HLA-F* | 0.014 | Co-expression |
| *CD74* | *HLA-A* | 0.014 | Co-expression |
| *CD74* | *HLA-DRA* | 0.017 | Co-expression |
| *CD74* | *HLA-G* | 0.015 | Co-expression |
| *CD74* | *B2M* | 0.016 | Co-expression |
| *CD74* | *HLA-E* | 0.010 | Co-expression |
| *CD74* | *TAP1* | 0.008 | Co-expression |
| *CD74* | *HLA-DPB1* | 0.016 | Co-expression |
| *CD74* | *HLA-DMA* | 0.011 | Co-expression |
| *CD74* | *HLA-C* | 0.008 | Co-expression |
| *CD74* | *HLA-A* | 0.006 | Co-expression |
| *CD74* | *HLA-DRA* | 0.023 | Co-expression |
| *CD74* | *HLA-G* | 0.011 | Co-expression |
| *CD74* | *HLA-B* | 0.008 | Co-expression |
| *CD74* | *HLA-E* | 0.011 | Co-expression |
| *CD74* | *TAP1* | 0.009 | Co-expression |
| *CD74* | *HLA-DRB1* | 0.016 | Co-expression |
| *CD74* | *HLA-DPB1* | 0.031 | Co-expression |
| *CD74* | *HLA-DMA* | 0.013 | Co-expression |
| *CD74* | *HLA-DQB1* | 0.029 | Co-expression |
| *CD74* | *HLA-DRA* | 0.028 | Co-expression |
| *CD74* | *HLA-G* | 0.028 | Co-expression |
| *CD74* | *B2M* | 0.026 | Co-expression |
| *CD74* | *HLA-DRA* | 0.019 | Co-expression |
| *CD74* | *HLA-DRB1* | 0.020 | Co-expression |
| *CD74* | *HLA-DPB1* | 0.019 | Co-expression |
| *CD74* | *HLA-DMA* | 0.015 | Co-expression |
| *CD74* | *HLA-DQB1* | 0.021 | Co-expression |
| *CD74* | *HLA-C* | 0.008 | Co-expression |
| *CD74* | *HLA-F* | 0.006 | Co-expression |
| *CD74* | *HLA-A* | 0.008 | Co-expression |
| *CD74* | *HLA-DRA* | 0.009 | Co-expression |
| *CD74* | *HLA-G* | 0.006 | Co-expression |
| *CD74* | *HLA-B* | 0.007 | Co-expression |
| *CD74* | *HLA-E* | 0.007 | Co-expression |
| *CD74* | *HLA-DRB1* | 0.009 | Co-expression |
| *CD74* | *HLA-DPB1* | 0.009 | Co-expression |
| *CD74* | *HLA-DMA* | 0.008 | Co-expression |
| *CD74* | *HLA-DQB1* | 0.013 | Co-expression |
| *CD74* | *TBKBP1* | 0.011 | Co-expression |
| *CD74* | *HLA-A* | 0.009 | Co-expression |
| *CD74* | *HLA-DRA* | 0.010 | Co-expression |
| *CD74* | *HLA-DPB1* | 0.013 | Co-expression |
| *CD74* | *HLA-DMA* | 0.008 | Co-expression |
| *CD74* | *HLA-A* | 0.003 | Co-expression |
| *CD74* | *HLA-DRA* | 0.004 | Co-expression |
| *CD74* | *HLA-B* | 0.007 | Co-expression |
| *CD74* | *HLA-DRB1* | 0.006 | Co-expression |
| *CD74* | *HLA-DPB1* | 0.003 | Co-expression |
| *CD74* | *HLA-DQB1* | 0.003 | Co-expression |
| *CD74* | *HLA-F* | 0.006 | Co-localization |
| *CD74* | *HLA-DRA* | 0.005 | Co-localization |
| *CD74* | *HLA-G* | 0.008 | Co-localization |
| *CD74* | *B2M* | 0.006 | Co-localization |
| *CD74* | *HLA-E* | 0.005 | Co-localization |
| *CD74* | *TAP1* | 0.009 | Co-localization |
| *CD74* | *HLA-DPB1* | 0.006 | Co-localization |
| *CD74* | *HLA-DPB1* | 0.086 | Physical Interactions |
| *CD74* | *HLA-C* | 0.032 | Predicted |
| *CD74* | *HLA-G* | 0.044 | Predicted |
| *CD74* | *HLA-DRB1* | 0.040 | Predicted |
| *CD74* | *HLA-DPB1* | 0.127 | Predicted |
| *CD74* | *HLA-DMA* | 0.193 | Predicted |
| *HLA-A* | *HLA-C* | 0.037 | Co-expression |
| *HLA-A* | *HLA-F* | 0.038 | Co-expression |
| *HLA-A* | *HLA-C* | 0.034 | Co-expression |
| *HLA-A* | *HLA-F* | 0.017 | Co-expression |
| *HLA-A* | *HLA-C* | 0.034 | Co-expression |
| *HLA-A* | *HLA-F* | 0.030 | Co-expression |
| *HLA-A* | *HLA-C* | 0.029 | Co-expression |
| *HLA-A* | *HLA-F* | 0.024 | Co-expression |
| *HLA-A* | *HLA-C* | 0.021 | Co-expression |
| *HLA-A* | *HLA-F* | 0.019 | Co-expression |
| *HLA-A* | *PGBD5* | 0.007 | Co-expression |
| *HLA-A* | *TBKBP1* | 0.011 | Co-expression |
| *HLA-A* | *HLA-C* | 0.016 | Co-expression |
| *HLA-A* | *HLA-F* | 0.012 | Co-expression |
| *HLA-A* | *HLA-F* | 0.023 | Co-expression |
| *HLA-A* | *HLA-C* | 0.031 | Co-expression |
| *HLA-A* | *HLA-F* | 0.028 | Co-expression |
| *HLA-A* | *HLA-C* | 0.024 | Co-expression |
| *HLA-A* | *HLA-F* | 0.011 | Co-expression |
| *HLA-A* | *AOAH* | 0.011 | Co-expression |
| *HLA-A* | *HLA-F* | 0.020 | Co-expression |
| *HLA-A* | *HLA-C* | 0.003 | Co-expression |
| *HLA-A* | *HLA-F* | 0.003 | Co-expression |
| *HLA-A* | *HLA-C* | 0.013 | Co-expression |
| *HLA-A* | *HLA-C* | 0.027 | Co-expression |
| *HLA-A* | *HLA-F* | 0.025 | Co-expression |
| *HLA-A* | *HLA-C* | 0.009 | Co-expression |
| *HLA-A* | *HLA-F* | 0.006 | Co-expression |
| *HLA-A* | *HLA-F* | 0.019 | Co-expression |
| *HLA-A* | *HLA-C* | 0.005 | Co-expression |
| *HLA-A* | *HLA-C* | 0.027 | Physical Interactions |
| *HLA-A* | *HLA-C* | 0.017 | Predicted |
| *HLA-A* | *HLA-F* | 0.031 | Predicted |
| *HLA-A* | *HLA-C* | 0.044 | Shared protein domains |
| *HLA-A* | *HLA-F* | 0.034 | Shared protein domains |
| *HLA-A* | *HLA-C* | 0.055 | Shared protein domains |
| *HLA-A* | *HLA-F* | 0.032 | Shared protein domains |
| *HLA-B* | *HLA-C* | 0.039 | Co-expression |
| *HLA-B* | *HLA-F* | 0.036 | Co-expression |
| *HLA-B* | *HLA-A* | 0.037 | Co-expression |
| *HLA-B* | *HLA-DRA* | 0.009 | Co-expression |
| *HLA-B* | *HLA-G* | 0.037 | Co-expression |
| *HLA-B* | *HLA-C* | 0.032 | Co-expression |
| *HLA-B* | *HLA-F* | 0.017 | Co-expression |
| *HLA-B* | *HLA-A* | 0.029 | Co-expression |
| *HLA-B* | *HLA-DRA* | 0.010 | Co-expression |
| *HLA-B* | *HLA-G* | 0.024 | Co-expression |
| *HLA-B* | *HLA-C* | 0.039 | Co-expression |
| *HLA-B* | *HLA-F* | 0.031 | Co-expression |
| *HLA-B* | *HLA-A* | 0.030 | Co-expression |
| *HLA-B* | *HLA-G* | 0.039 | Co-expression |
| *HLA-B* | *HLA-C* | 0.029 | Co-expression |
| *HLA-B* | *HLA-F* | 0.024 | Co-expression |
| *HLA-B* | *HLA-A* | 0.024 | Co-expression |
| *HLA-B* | *HLA-G* | 0.031 | Co-expression |
| *HLA-B* | *HLA-C* | 0.031 | Co-expression |
| *HLA-B* | *HLA-F* | 0.036 | Co-expression |
| *HLA-B* | *HLA-A* | 0.012 | Co-expression |
| *HLA-B* | *HLA-DRA* | 0.013 | Co-expression |
| *HLA-B* | *HLA-G* | 0.024 | Co-expression |
| *HLA-B* | *HLA-C* | 0.029 | Co-expression |
| *HLA-B* | *HLA-F* | 0.026 | Co-expression |
| *HLA-B* | *HLA-A* | 0.032 | Co-expression |
| *HLA-B* | *HLA-G* | 0.027 | Co-expression |
| *HLA-B* | *HLA-C* | 0.027 | Co-expression |
| *HLA-B* | *HLA-A* | 0.029 | Co-expression |
| *HLA-B* | *HLA-DRA* | 0.022 | Co-expression |
| *HLA-B* | *HLA-G* | 0.023 | Co-expression |
| *HLA-B* | *HLA-C* | 0.004 | Co-expression |
| *HLA-B* | *HLA-F* | 0.003 | Co-expression |
| *HLA-B* | *HLA-A* | 0.003 | Co-expression |
| *HLA-B* | *HLA-DRA* | 0.006 | Co-expression |
| *HLA-B* | *HLA-G* | 0.005 | Co-expression |
| *HLA-B* | *HLA-C* | 0.029 | Co-expression |
| *HLA-B* | *HLA-A* | 0.019 | Co-expression |
| *HLA-B* | *HLA-C* | 0.008 | Co-expression |
| *HLA-B* | *HLA-F* | 0.005 | Co-expression |
| *HLA-B* | *HLA-A* | 0.007 | Co-expression |
| *HLA-B* | *HLA-DRA* | 0.006 | Co-expression |
| *HLA-B* | *HLA-G* | 0.006 | Co-expression |
| *HLA-B* | *HLA-A* | 0.006 | Co-expression |
| *HLA-B* | *HLA-DRA* | 0.007 | Co-expression |
| *HLA-B* | *HLA-C* | 0.012 | Physical Interactions |
| *HLA-B* | *HLA-A* | 0.029 | Physical Interactions |
| *HLA-B* | *HLA-C* | 0.012 | Predicted |
| *HLA-B* | *HLA-F* | 0.022 | Predicted |
| *HLA-B* | *HLA-A* | 0.012 | Predicted |
| *HLA-B* | *HLA-DRA* | 0.022 | Predicted |
| *HLA-B* | *HLA-G* | 0.017 | Predicted |
| *HLA-B* | *HLA-C* | 0.044 | Shared protein domains |
| *HLA-B* | *HLA-F* | 0.034 | Shared protein domains |
| *HLA-B* | *HLA-A* | 0.044 | Shared protein domains |
| *HLA-B* | *HLA-DRA* | 0.019 | Shared protein domains |
| *HLA-B* | *HLA-G* | 0.034 | Shared protein domains |
| *HLA-B* | *HLA-C* | 0.055 | Shared protein domains |
| *HLA-B* | *HLA-F* | 0.032 | Shared protein domains |
| *HLA-B* | *HLA-A* | 0.055 | Shared protein domains |
| *HLA-B* | *HLA-DRA* | 0.014 | Shared protein domains |
| *HLA-B* | *HLA-G* | 0.032 | Shared protein domains |
| *HLA-DMA* | *HLA-DRA* | 0.008 | Co-expression |
| *HLA-DMA* | *HLA-B* | 0.010 | Co-expression |
| *HLA-DMA* | *HLA-DRB1* | 0.007 | Co-expression |
| *HLA-DMA* | *HLA-DPB1* | 0.011 | Co-expression |
| *HLA-DMA* | *HLA-C* | 0.008 | Co-expression |
| *HLA-DMA* | *HLA-F* | 0.006 | Co-expression |
| *HLA-DMA* | *AOAH* | 0.005 | Co-expression |
| *HLA-DMA* | *HLA-DRA* | 0.005 | Co-expression |
| *HLA-DMA* | *HLA-G* | 0.007 | Co-expression |
| *HLA-DMA* | *HLA-B* | 0.007 | Co-expression |
| *HLA-DMA* | *HLA-E* | 0.005 | Co-expression |
| *HLA-DMA* | *TAP1* | 0.007 | Co-expression |
| *HLA-DMA* | *HLA-DRB1* | 0.004 | Co-expression |
| *HLA-DMA* | *HLA-DPB1* | 0.005 | Co-expression |
| *HLA-DMA* | *HLA-DRA* | 0.021 | Co-expression |
| *HLA-DMA* | *HLA-DRB1* | 0.022 | Co-expression |
| *HLA-DMA* | *HLA-DPB1* | 0.024 | Co-expression |
| *HLA-DMA* | *HLA-C* | 0.011 | Co-expression |
| *HLA-DMA* | *HLA-F* | 0.015 | Co-expression |
| *HLA-DMA* | *HLA-A* | 0.014 | Co-expression |
| *HLA-DMA* | *HLA-DRA* | 0.018 | Co-expression |
| *HLA-DMA* | *B2M* | 0.011 | Co-expression |
| *HLA-DMA* | *HLA-DRB1* | 0.015 | Co-expression |
| *HLA-DMA* | *HLA-DPB1* | 0.009 | Co-expression |
| *HLA-DMA* | *HLA-DRA* | 0.009 | Co-expression |
| *HLA-DMA* | *HLA-DPB1* | 0.016 | Co-expression |
| *HLA-DMA* | *HLA-F* | 0.021 | Co-expression |
| *HLA-DMA* | *HLA-A* | 0.019 | Co-expression |
| *HLA-DMA* | *HLA-DRA* | 0.022 | Co-expression |
| *HLA-DMA* | *B2M* | 0.014 | Co-expression |
| *HLA-DMA* | *TAP1* | 0.019 | Co-expression |
| *HLA-DMA* | *HLA-DRB1* | 0.024 | Co-expression |
| *HLA-DMA* | *HLA-DPB1* | 0.025 | Co-expression |
| *HLA-DMA* | *HLA-F* | 0.011 | Co-expression |
| *HLA-DMA* | *HLA-A* | 0.011 | Co-expression |
| *HLA-DMA* | *HLA-DRA* | 0.017 | Co-expression |
| *HLA-DMA* | *HLA-G* | 0.013 | Co-expression |
| *HLA-DMA* | *B2M* | 0.013 | Co-expression |
| *HLA-DMA* | *HLA-E* | 0.010 | Co-expression |
| *HLA-DMA* | *TAP1* | 0.008 | Co-expression |
| *HLA-DMA* | *HLA-DRB1* | 0.012 | Co-expression |
| *HLA-DMA* | *HLA-DPB1* | 0.008 | Co-expression |
| *HLA-DMA* | *HLA-C* | 0.012 | Co-expression |
| *HLA-DMA* | *HLA-DRA* | 0.021 | Co-expression |
| *HLA-DMA* | *HLA-B* | 0.011 | Co-expression |
| *HLA-DMA* | *HLA-DRB1* | 0.020 | Co-expression |
| *HLA-DMA* | *HLA-DPB1* | 0.019 | Co-expression |
| *HLA-DMA* | *HLA-F* | 0.008 | Co-expression |
| *HLA-DMA* | *AOAH* | 0.010 | Co-expression |
| *HLA-DMA* | *HLA-A* | 0.009 | Co-expression |
| *HLA-DMA* | *HLA-DRA* | 0.008 | Co-expression |
| *HLA-DMA* | *HLA-G* | 0.007 | Co-expression |
| *HLA-DMA* | *B2M* | 0.010 | Co-expression |
| *HLA-DMA* | *HLA-E* | 0.006 | Co-expression |
| *HLA-DMA* | *HLA-DPB1* | 0.011 | Co-expression |
| *HLA-DMA* | *HLA-F* | 0.009 | Co-expression |
| *HLA-DMA* | *AOAH* | 0.011 | Co-expression |
| *HLA-DMA* | *HLA-DRA* | 0.009 | Co-expression |
| *HLA-DMA* | *HLA-G* | 0.010 | Co-expression |
| *HLA-DMA* | *B2M* | 0.009 | Co-expression |
| *HLA-DMA* | *HLA-E* | 0.008 | Co-expression |
| *HLA-DMA* | *TAP1* | 0.007 | Co-expression |
| *HLA-DMA* | *HLA-DPB1* | 0.011 | Co-expression |
| *HLA-DMA* | *HLA-C* | 0.005 | Co-expression |
| *HLA-DMA* | *HLA-F* | 0.004 | Co-expression |
| *HLA-DMA* | *HLA-A* | 0.004 | Co-expression |
| *HLA-DMA* | *HLA-DRA* | 0.011 | Co-expression |
| *HLA-DMA* | *HLA-G* | 0.006 | Co-expression |
| *HLA-DMA* | *HLA-B* | 0.004 | Co-expression |
| *HLA-DMA* | *B2M* | 0.010 | Co-expression |
| *HLA-DMA* | *HLA-E* | 0.006 | Co-expression |
| *HLA-DMA* | *TAP1* | 0.006 | Co-expression |
| *HLA-DMA* | *HLA-DRB1* | 0.008 | Co-expression |
| *HLA-DMA* | *HLA-DPB1* | 0.015 | Co-expression |
| *HLA-DMA* | *HLA-A* | 0.007 | Co-expression |
| *HLA-DMA* | *HLA-DRA* | 0.003 | Co-expression |
| *HLA-DMA* | *HLA-DRB1* | 0.003 | Co-expression |
| *HLA-DMA* | *HLA-DPB1* | 0.003 | Co-expression |
| *HLA-DMA* | *HLA-DRA* | 0.016 | Co-expression |
| *HLA-DMA* | *HLA-DRB1* | 0.017 | Co-expression |
| *HLA-DMA* | *HLA-DPB1* | 0.017 | Co-expression |
| *HLA-DMA* | *HLA-DRA* | 0.030 | Co-expression |
| *HLA-DMA* | *HLA-DPB1* | 0.011 | Co-expression |
| *HLA-DMA* | *HLA-F* | 0.006 | Co-expression |
| *HLA-DMA* | *HLA-DRA* | 0.008 | Co-expression |
| *HLA-DMA* | *HLA-E* | 0.006 | Co-expression |
| *HLA-DMA* | *HLA-DRB1* | 0.009 | Co-expression |
| *HLA-DMA* | *HLA-DPB1* | 0.008 | Co-expression |
| *HLA-DMA* | *TBKBP1* | 0.012 | Co-expression |
| *HLA-DMA* | *HLA-DRA* | 0.010 | Co-expression |
| *HLA-DMA* | *HLA-DPB1* | 0.017 | Co-expression |
| *HLA-DMA* | *HLA-A* | 0.004 | Co-expression |
| *HLA-DMA* | *HLA-DRA* | 0.005 | Co-expression |
| *HLA-DMA* | *HLA-DRB1* | 0.008 | Co-expression |
| *HLA-DMA* | *HLA-DPB1* | 0.004 | Co-expression |
| *HLA-DMA* | *HLA-C* | 0.100 | Predicted |
| *HLA-DMA* | *HLA-DRB1* | 0.126 | Predicted |
| *HLA-DMA* | *HLA-C* | 0.018 | Shared protein domains |
| *HLA-DMA* | *HLA-F* | 0.019 | Shared protein domains |
| *HLA-DMA* | *HLA-A* | 0.019 | Shared protein domains |
| *HLA-DMA* | *HLA-DRA* | 0.042 | Shared protein domains |
| *HLA-DMA* | *HLA-G* | 0.019 | Shared protein domains |
| *HLA-DMA* | *HLA-B* | 0.019 | Shared protein domains |
| *HLA-DMA* | *B2M* | 0.018 | Shared protein domains |
| *HLA-DMA* | *HLA-E* | 0.019 | Shared protein domains |
| *HLA-DMA* | *HLA-DRB1* | 0.021 | Shared protein domains |
| *HLA-DMA* | *HLA-DPB1* | 0.021 | Shared protein domains |
| *HLA-DMA* | *HLA-C* | 0.014 | Shared protein domains |
| *HLA-DMA* | *HLA-F* | 0.015 | Shared protein domains |
| *HLA-DMA* | *HLA-A* | 0.014 | Shared protein domains |
| *HLA-DMA* | *HLA-DRA* | 0.078 | Shared protein domains |
| *HLA-DMA* | *HLA-G* | 0.015 | Shared protein domains |
| *HLA-DMA* | *HLA-B* | 0.014 | Shared protein domains |
| *HLA-DMA* | *B2M* | 0.023 | Shared protein domains |
| *HLA-DMA* | *HLA-E* | 0.014 | Shared protein domains |
| *HLA-DMA* | *HLA-DRB1* | 0.017 | Shared protein domains |
| *HLA-DMA* | *HLA-DPB1* | 0.017 | Shared protein domains |
| *HLA-DMB* | *AOAH* | 0.012 | Co-expression |
| *HLA-DMB* | *HLA-DRA* | 0.013 | Co-expression |
| *HLA-DMB* | *HLA-DRB1* | 0.010 | Co-expression |
| *HLA-DMB* | *HLA-DPB1* | 0.018 | Co-expression |
| *HLA-DMB* | *HLA-DMA* | 0.015 | Co-expression |
| *HLA-DMB* | *HLA-DPA1* | 0.010 | Co-expression |
| *HLA-DMB* | *HLA-DRB5* | 0.019 | Co-expression |
| *HLA-DMB* | *AOAH* | 0.008 | Co-expression |
| *HLA-DMB* | *HLA-DRA* | 0.007 | Co-expression |
| *HLA-DMB* | *HLA-DRB1* | 0.005 | Co-expression |
| *HLA-DMB* | *HLA-DPB1* | 0.006 | Co-expression |
| *HLA-DMB* | *HLA-DMA* | 0.006 | Co-expression |
| *HLA-DMB* | *HLA-DQB1* | 0.013 | Co-expression |
| *HLA-DMB* | *CD74* | 0.005 | Co-expression |
| *HLA-DMB* | *HLA-DPA1* | 0.009 | Co-expression |
| *HLA-DMB* | *HLA-DRA* | 0.019 | Co-expression |
| *HLA-DMB* | *HLA-DPB1* | 0.025 | Co-expression |
| *HLA-DMB* | *HLA-DMA* | 0.022 | Co-expression |
| *HLA-DMB* | *CD74* | 0.018 | Co-expression |
| *HLA-DMB* | *HLA-DPA1* | 0.022 | Co-expression |
| *HLA-DMB* | *HLA-DMA* | 0.025 | Co-expression |
| *HLA-DMB* | *HLA-DRA* | 0.007 | Co-expression |
| *HLA-DMB* | *HLA-DPB1* | 0.013 | Co-expression |
| *HLA-DMB* | *HLA-DMA* | 0.011 | Co-expression |
| *HLA-DMB* | *HLA-DQB1* | 0.009 | Co-expression |
| *HLA-DMB* | *HLA-DPA1* | 0.014 | Co-expression |
| *HLA-DMB* | *HLA-F* | 0.020 | Co-expression |
| *HLA-DMB* | *HLA-DRA* | 0.019 | Co-expression |
| *HLA-DMB* | *TAP1* | 0.018 | Co-expression |
| *HLA-DMB* | *HLA-DRB1* | 0.021 | Co-expression |
| *HLA-DMB* | *HLA-DPB1* | 0.024 | Co-expression |
| *HLA-DMB* | *HLA-DMA* | 0.022 | Co-expression |
| *HLA-DMB* | *HLA-DQB1* | 0.022 | Co-expression |
| *HLA-DMB* | *CD74* | 0.017 | Co-expression |
| *HLA-DMB* | *HLA-DRA* | 0.031 | Co-expression |
| *HLA-DMB* | *HLA-DRB1* | 0.020 | Co-expression |
| *HLA-DMB* | *HLA-DPB1* | 0.015 | Co-expression |
| *HLA-DMB* | *HLA-DMA* | 0.019 | Co-expression |
| *HLA-DMB* | *CD74* | 0.025 | Co-expression |
| *HLA-DMB* | *HLA-DPA1* | 0.016 | Co-expression |
| *HLA-DMB* | *HLA-DRB5* | 0.029 | Co-expression |
| *HLA-DMB* | *HLA-C* | 0.014 | Co-expression |
| *HLA-DMB* | *HLA-DRA* | 0.022 | Co-expression |
| *HLA-DMB* | *HLA-B* | 0.014 | Co-expression |
| *HLA-DMB* | *HLA-DRB1* | 0.020 | Co-expression |
| *HLA-DMB* | *HLA-DPB1* | 0.020 | Co-expression |
| *HLA-DMB* | *HLA-DMA* | 0.017 | Co-expression |
| *HLA-DMB* | *HLA-DQB1* | 0.022 | Co-expression |
| *HLA-DMB* | *CD74* | 0.017 | Co-expression |
| *HLA-DMB* | *HLA-DPA1* | 0.021 | Co-expression |
| *HLA-DMB* | *HLA-F* | 0.008 | Co-expression |
| *HLA-DMB* | *AOAH* | 0.011 | Co-expression |
| *HLA-DMB* | *HLA-A* | 0.008 | Co-expression |
| *HLA-DMB* | *HLA-DRA* | 0.009 | Co-expression |
| *HLA-DMB* | *HLA-G* | 0.007 | Co-expression |
| *HLA-DMB* | *B2M* | 0.010 | Co-expression |
| *HLA-DMB* | *HLA-E* | 0.006 | Co-expression |
| *HLA-DMB* | *TAP1* | 0.009 | Co-expression |
| *HLA-DMB* | *HLA-DPB1* | 0.012 | Co-expression |
| *HLA-DMB* | *HLA-DMA* | 0.009 | Co-expression |
| *HLA-DMB* | *HLA-DQB1* | 0.013 | Co-expression |
| *HLA-DMB* | *CD74* | 0.009 | Co-expression |
| *HLA-DMB* | *HLA-DPA1* | 0.010 | Co-expression |
| *HLA-DMB* | *HLA-F* | 0.007 | Co-expression |
| *HLA-DMB* | *AOAH* | 0.009 | Co-expression |
| *HLA-DMB* | *HLA-DRA* | 0.007 | Co-expression |
| *HLA-DMB* | *HLA-G* | 0.007 | Co-expression |
| *HLA-DMB* | *B2M* | 0.009 | Co-expression |
| *HLA-DMB* | *HLA-E* | 0.006 | Co-expression |
| *HLA-DMB* | *TAP1* | 0.005 | Co-expression |
| *HLA-DMB* | *HLA-DPB1* | 0.009 | Co-expression |
| *HLA-DMB* | *HLA-DMA* | 0.007 | Co-expression |
| *HLA-DMB* | *HLA-DQB1* | 0.008 | Co-expression |
| *HLA-DMB* | *CD74* | 0.008 | Co-expression |
| *HLA-DMB* | *HLA-DPA1* | 0.009 | Co-expression |
| *HLA-DMB* | *HLA-DRA* | 0.009 | Co-expression |
| *HLA-DMB* | *HLA-DRB1* | 0.006 | Co-expression |
| *HLA-DMB* | *HLA-DPB1* | 0.013 | Co-expression |
| *HLA-DMB* | *HLA-DMA* | 0.006 | Co-expression |
| *HLA-DMB* | *HLA-DQB1* | 0.013 | Co-expression |
| *HLA-DMB* | *CD74* | 0.011 | Co-expression |
| *HLA-DMB* | *HLA-DPA1* | 0.009 | Co-expression |
| *HLA-DMB* | *HLA-DMA* | 0.014 | Co-expression |
| *HLA-DMB* | *HLA-DPA1* | 0.019 | Co-expression |
| *HLA-DMB* | *HLA-DRA* | 0.017 | Co-expression |
| *HLA-DMB* | *HLA-DRB1* | 0.019 | Co-expression |
| *HLA-DMB* | *HLA-DPB1* | 0.018 | Co-expression |
| *HLA-DMB* | *HLA-DMA* | 0.015 | Co-expression |
| *HLA-DMB* | *HLA-DQB1* | 0.020 | Co-expression |
| *HLA-DMB* | *CD74* | 0.016 | Co-expression |
| *HLA-DMB* | *HLA-DPA1* | 0.019 | Co-expression |
| *HLA-DMB* | *AOAH* | 0.011 | Co-expression |
| *HLA-DMB* | *HLA-DRA* | 0.014 | Co-expression |
| *HLA-DMB* | *HLA-DPB1* | 0.007 | Co-expression |
| *HLA-DMB* | *HLA-DMA* | 0.016 | Co-expression |
| *HLA-DMB* | *HLA-DQB1* | 0.014 | Co-expression |
| *HLA-DMB* | *HLA-DPA1* | 0.009 | Co-expression |
| *HLA-DMB* | *HLA-DRA* | 0.013 | Co-expression |
| *HLA-DMB* | *HLA-DRB1* | 0.013 | Co-expression |
| *HLA-DMB* | *HLA-DPB1* | 0.013 | Co-expression |
| *HLA-DMB* | *HLA-DMA* | 0.012 | Co-expression |
| *HLA-DMB* | *HLA-DQB1* | 0.020 | Co-expression |
| *HLA-DMB* | *CD74* | 0.012 | Co-expression |
| *HLA-DMB* | *HLA-DPA1* | 0.010 | Co-expression |
| *HLA-DMB* | *HLA-DRB5* | 0.014 | Co-expression |
| *HLA-DMB* | *HLA-DRA* | 0.009 | Co-expression |
| *HLA-DMB* | *HLA-DPB1* | 0.017 | Co-expression |
| *HLA-DMB* | *HLA-DMA* | 0.022 | Co-expression |
| *HLA-DMB* | *HLA-DPA1* | 0.015 | Co-expression |
| *HLA-DMB* | *HLA-DRA* | 0.006 | Co-expression |
| *HLA-DMB* | *HLA-DRB1* | 0.009 | Co-expression |
| *HLA-DMB* | *HLA-DPB1* | 0.005 | Co-expression |
| *HLA-DMB* | *HLA-DMA* | 0.010 | Co-expression |
| *HLA-DMB* | *HLA-DQB1* | 0.005 | Co-expression |
| *HLA-DMB* | *HLA-DPA1* | 0.005 | Co-expression |
| *HLA-DMB* | *HLA-DRA* | 0.010 | Co-localization |
| *HLA-DMB* | *HLA-E* | 0.006 | Co-localization |
| *HLA-DMB* | *HLA-DPB1* | 0.012 | Co-localization |
| *HLA-DMB* | *CD74* | 0.009 | Co-localization |
| *HLA-DMB* | *HLA-DRA* | 0.082 | Physical Interactions |
| *HLA-DMB* | *B2M* | 0.072 | Predicted |
| *HLA-DMB* | *HLA-E* | 0.059 | Predicted |
| *HLA-DMB* | *HLA-DRB1* | 0.055 | Predicted |
| *HLA-DMB* | *CD74* | 0.084 | Predicted |
| *HLA-DMB* | *HLA-C* | 0.018 | Shared protein domains |
| *HLA-DMB* | *HLA-F* | 0.019 | Shared protein domains |
| *HLA-DMB* | *HLA-A* | 0.019 | Shared protein domains |
| *HLA-DMB* | *HLA-DRA* | 0.021 | Shared protein domains |
| *HLA-DMB* | *HLA-G* | 0.019 | Shared protein domains |
| *HLA-DMB* | *HLA-B* | 0.019 | Shared protein domains |
| *HLA-DMB* | *B2M* | 0.018 | Shared protein domains |
| *HLA-DMB* | *HLA-E* | 0.019 | Shared protein domains |
| *HLA-DMB* | *HLA-DRB1* | 0.031 | Shared protein domains |
| *HLA-DMB* | *HLA-DPB1* | 0.031 | Shared protein domains |
| *HLA-DMB* | *HLA-DMA* | 0.021 | Shared protein domains |
| *HLA-DMB* | *HLA-DQB1* | 0.031 | Shared protein domains |
| *HLA-DMB* | *HLA-DPA1* | 0.021 | Shared protein domains |
| *HLA-DMB* | *HLA-DRB5* | 0.031 | Shared protein domains |
| *HLA-DMB* | *HLA-C* | 0.014 | Shared protein domains |
| *HLA-DMB* | *HLA-F* | 0.015 | Shared protein domains |
| *HLA-DMB* | *HLA-A* | 0.014 | Shared protein domains |
| *HLA-DMB* | *HLA-DRA* | 0.017 | Shared protein domains |
| *HLA-DMB* | *HLA-G* | 0.015 | Shared protein domains |
| *HLA-DMB* | *HLA-B* | 0.014 | Shared protein domains |
| *HLA-DMB* | *B2M* | 0.023 | Shared protein domains |
| *HLA-DMB* | *HLA-E* | 0.014 | Shared protein domains |
| *HLA-DMB* | *HLA-DRB1* | 0.047 | Shared protein domains |
| *HLA-DMB* | *HLA-DPB1* | 0.047 | Shared protein domains |
| *HLA-DMB* | *HLA-DMA* | 0.017 | Shared protein domains |
| *HLA-DMB* | *HLA-DQB1* | 0.047 | Shared protein domains |
| *HLA-DMB* | *HLA-DPA1* | 0.017 | Shared protein domains |
| *HLA-DMB* | *HLA-DRB5* | 0.047 | Shared protein domains |
| *HLA-DPA1* | *HLA-C* | 0.008 | Co-expression |
| *HLA-DPA1* | *HLA-F* | 0.007 | Co-expression |
| *HLA-DPA1* | *AOAH* | 0.006 | Co-expression |
| *HLA-DPA1* | *HLA-DRA* | 0.006 | Co-expression |
| *HLA-DPA1* | *HLA-G* | 0.008 | Co-expression |
| *HLA-DPA1* | *HLA-B* | 0.007 | Co-expression |
| *HLA-DPA1* | *HLA-DRB1* | 0.005 | Co-expression |
| *HLA-DPA1* | *HLA-DPB1* | 0.009 | Co-expression |
| *HLA-DPA1* | *HLA-DMA* | 0.006 | Co-expression |
| *HLA-DPA1* | *HLA-DQB1* | 0.011 | Co-expression |
| *HLA-DPA1* | *CD74* | 0.005 | Co-expression |
| *HLA-DPA1* | *HLA-DRA* | 0.008 | Co-expression |
| *HLA-DPA1* | *HLA-DRB1* | 0.006 | Co-expression |
| *HLA-DPA1* | *HLA-DPB1* | 0.008 | Co-expression |
| *HLA-DPA1* | *HLA-DMA* | 0.006 | Co-expression |
| *HLA-DPA1* | *HLA-DQB1* | 0.016 | Co-expression |
| *HLA-DPA1* | *CD74* | 0.005 | Co-expression |
| *HLA-DPA1* | *HLA-DRA* | 0.025 | Co-expression |
| *HLA-DPA1* | *HLA-DRB1* | 0.022 | Co-expression |
| *HLA-DPA1* | *HLA-DMA* | 0.026 | Co-expression |
| *HLA-DPA1* | *HLA-DQB1* | 0.030 | Co-expression |
| *HLA-DPA1* | *CD74* | 0.028 | Co-expression |
| *HLA-DPA1* | *HLA-C* | 0.006 | Co-expression |
| *HLA-DPA1* | *HLA-A* | 0.007 | Co-expression |
| *HLA-DPA1* | *HLA-DRA* | 0.009 | Co-expression |
| *HLA-DPA1* | *B2M* | 0.005 | Co-expression |
| *HLA-DPA1* | *TAP1* | 0.006 | Co-expression |
| *HLA-DPA1* | *HLA-DRB1* | 0.008 | Co-expression |
| *HLA-DPA1* | *HLA-DPB1* | 0.005 | Co-expression |
| *HLA-DPA1* | *HLA-DMA* | 0.008 | Co-expression |
| *HLA-DPA1* | *HLA-DQB1* | 0.005 | Co-expression |
| *HLA-DPA1* | *HLA-DRA* | 0.019 | Co-expression |
| *HLA-DPA1* | *HLA-DMA* | 0.016 | Co-expression |
| *HLA-DPA1* | *CD74* | 0.016 | Co-expression |
| *HLA-DPA1* | *HLA-DRA* | 0.014 | Co-expression |
| *HLA-DPA1* | *HLA-DRB1* | 0.009 | Co-expression |
| *HLA-DPA1* | *HLA-DPB1* | 0.008 | Co-expression |
| *HLA-DPA1* | *HLA-DMA* | 0.009 | Co-expression |
| *HLA-DPA1* | *HLA-DQB1* | 0.011 | Co-expression |
| *HLA-DPA1* | *CD74* | 0.012 | Co-expression |
| *HLA-DPA1* | *HLA-C* | 0.016 | Co-expression |
| *HLA-DPA1* | *HLA-A* | 0.016 | Co-expression |
| *HLA-DPA1* | *HLA-DRA* | 0.028 | Co-expression |
| *HLA-DPA1* | *HLA-G* | 0.013 | Co-expression |
| *HLA-DPA1* | *HLA-B* | 0.016 | Co-expression |
| *HLA-DPA1* | *B2M* | 0.017 | Co-expression |
| *HLA-DPA1* | *HLA-DRB1* | 0.027 | Co-expression |
| *HLA-DPA1* | *HLA-DPB1* | 0.026 | Co-expression |
| *HLA-DPA1* | *HLA-DMA* | 0.020 | Co-expression |
| *HLA-DPA1* | *HLA-DQB1* | 0.030 | Co-expression |
| *HLA-DPA1* | *CD74* | 0.023 | Co-expression |
| *HLA-DPA1* | *HLA-F* | 0.009 | Co-expression |
| *HLA-DPA1* | *AOAH* | 0.010 | Co-expression |
| *HLA-DPA1* | *HLA-A* | 0.009 | Co-expression |
| *HLA-DPA1* | *HLA-DRA* | 0.010 | Co-expression |
| *HLA-DPA1* | *HLA-G* | 0.008 | Co-expression |
| *HLA-DPA1* | *B2M* | 0.011 | Co-expression |
| *HLA-DPA1* | *HLA-E* | 0.006 | Co-expression |
| *HLA-DPA1* | *HLA-DPB1* | 0.013 | Co-expression |
| *HLA-DPA1* | *HLA-DMA* | 0.010 | Co-expression |
| *HLA-DPA1* | *HLA-DQB1* | 0.014 | Co-expression |
| *HLA-DPA1* | *CD74* | 0.011 | Co-expression |
| *HLA-DPA1* | *HLA-DRA* | 0.016 | Co-expression |
| *HLA-DPA1* | *B2M* | 0.016 | Co-expression |
| *HLA-DPA1* | *HLA-E* | 0.012 | Co-expression |
| *HLA-DPA1* | *HLA-DPB1* | 0.017 | Co-expression |
| *HLA-DPA1* | *HLA-DMA* | 0.011 | Co-expression |
| *HLA-DPA1* | *HLA-DQB1* | 0.017 | Co-expression |
| *HLA-DPA1* | *CD74* | 0.016 | Co-expression |
| *HLA-DPA1* | *HLA-C* | 0.006 | Co-expression |
| *HLA-DPA1* | *HLA-F* | 0.005 | Co-expression |
| *HLA-DPA1* | *HLA-A* | 0.005 | Co-expression |
| *HLA-DPA1* | *HLA-DRA* | 0.016 | Co-expression |
| *HLA-DPA1* | *HLA-G* | 0.008 | Co-expression |
| *HLA-DPA1* | *HLA-B* | 0.006 | Co-expression |
| *HLA-DPA1* | *B2M* | 0.014 | Co-expression |
| *HLA-DPA1* | *HLA-E* | 0.008 | Co-expression |
| *HLA-DPA1* | *TAP1* | 0.008 | Co-expression |
| *HLA-DPA1* | *HLA-DRB1* | 0.012 | Co-expression |
| *HLA-DPA1* | *HLA-DPB1* | 0.021 | Co-expression |
| *HLA-DPA1* | *HLA-DMA* | 0.010 | Co-expression |
| *HLA-DPA1* | *HLA-DQB1* | 0.020 | Co-expression |
| *HLA-DPA1* | *CD74* | 0.019 | Co-expression |
| *HLA-DPA1* | *HLA-F* | 0.028 | Co-expression |
| *HLA-DPA1* | *HLA-DRA* | 0.025 | Co-expression |
| *HLA-DPA1* | *CD74* | 0.022 | Co-expression |
| *HLA-DPA1* | *HLA-C* | 0.003 | Co-expression |
| *HLA-DPA1* | *AOAH* | 0.003 | Co-expression |
| *HLA-DPA1* | *HLA-A* | 0.003 | Co-expression |
| *HLA-DPA1* | *HLA-DRA* | 0.002 | Co-expression |
| *HLA-DPA1* | *B2M* | 0.004 | Co-expression |
| *HLA-DPA1* | *HLA-DRB1* | 0.002 | Co-expression |
| *HLA-DPA1* | *HLA-DPB1* | 0.002 | Co-expression |
| *HLA-DPA1* | *HLA-DMA* | 0.003 | Co-expression |
| *HLA-DPA1* | *HLA-DQB1* | 0.002 | Co-expression |
| *HLA-DPA1* | *HLA-DRA* | 0.025 | Co-expression |
| *HLA-DPA1* | *HLA-DRB1* | 0.028 | Co-expression |
| *HLA-DPA1* | *HLA-DPB1* | 0.028 | Co-expression |
| *HLA-DPA1* | *HLA-DMA* | 0.019 | Co-expression |
| *HLA-DPA1* | *HLA-DQB1* | 0.033 | Co-expression |
| *HLA-DPA1* | *CD74* | 0.023 | Co-expression |
| *HLA-DPA1* | *AOAH* | 0.011 | Co-expression |
| *HLA-DPA1* | *HLA-DRA* | 0.019 | Co-expression |
| *HLA-DPA1* | *HLA-DPB1* | 0.010 | Co-expression |
| *HLA-DPA1* | *HLA-DMA* | 0.019 | Co-expression |
| *HLA-DPA1* | *HLA-DQB1* | 0.015 | Co-expression |
| *HLA-DPA1* | *LRRK2* | 0.006 | Co-expression |
| *HLA-DPA1* | *HLA-DRA* | 0.007 | Co-expression |
| *HLA-DPA1* | *HLA-DRB1* | 0.008 | Co-expression |
| *HLA-DPA1* | *HLA-DPB1* | 0.007 | Co-expression |
| *HLA-DPA1* | *HLA-DMA* | 0.007 | Co-expression |
| *HLA-DPA1* | *HLA-DQB1* | 0.011 | Co-expression |
| *HLA-DPA1* | *CD74* | 0.007 | Co-expression |
| *HLA-DPA1* | *HLA-DRA* | 0.020 | Co-expression |
| *HLA-DPA1* | *HLA-DPB1* | 0.024 | Co-expression |
| *HLA-DPA1* | *HLA-DMA* | 0.013 | Co-expression |
| *HLA-DPA1* | *CD74* | 0.013 | Co-expression |
| *HLA-DPA1* | *HLA-A* | 0.003 | Co-expression |
| *HLA-DPA1* | *HLA-DRA* | 0.004 | Co-expression |
| *HLA-DPA1* | *HLA-G* | 0.006 | Co-expression |
| *HLA-DPA1* | *HLA-B* | 0.006 | Co-expression |
| *HLA-DPA1* | *B2M* | 0.008 | Co-expression |
| *HLA-DPA1* | *TAP1* | 0.007 | Co-expression |
| *HLA-DPA1* | *HLA-DRB1* | 0.005 | Co-expression |
| *HLA-DPA1* | *HLA-DPB1* | 0.003 | Co-expression |
| *HLA-DPA1* | *HLA-DMA* | 0.005 | Co-expression |
| *HLA-DPA1* | *HLA-DQB1* | 0.003 | Co-expression |
| *HLA-DPA1* | *CD74* | 0.003 | Co-expression |
| *HLA-DPA1* | *HLA-F* | 0.019 | Physical Interactions |
| *HLA-DPA1* | *HLA-A* | 0.014 | Physical Interactions |
| *HLA-DPA1* | *HLA-DPB1* | 0.009 | Physical Interactions |
| *HLA-DPA1* | *HLA-C* | 0.020 | Predicted |
| *HLA-DPA1* | *HLA-F* | 0.036 | Predicted |
| *HLA-DPA1* | *HLA-A* | 0.021 | Predicted |
| *HLA-DPA1* | *HLA-G* | 0.027 | Predicted |
| *HLA-DPA1* | *HLA-B* | 0.015 | Predicted |
| *HLA-DPA1* | *B2M* | 0.033 | Predicted |
| *HLA-DPA1* | *HLA-E* | 0.027 | Predicted |
| *HLA-DPA1* | *HLA-DRB1* | 0.025 | Predicted |
| *HLA-DPA1* | *CD74* | 0.038 | Predicted |
| *HLA-DPA1* | *HLA-C* | 0.018 | Shared protein domains |
| *HLA-DPA1* | *HLA-F* | 0.019 | Shared protein domains |
| *HLA-DPA1* | *HLA-A* | 0.019 | Shared protein domains |
| *HLA-DPA1* | *HLA-DRA* | 0.042 | Shared protein domains |
| *HLA-DPA1* | *HLA-G* | 0.019 | Shared protein domains |
| *HLA-DPA1* | *HLA-B* | 0.019 | Shared protein domains |
| *HLA-DPA1* | *B2M* | 0.018 | Shared protein domains |
| *HLA-DPA1* | *HLA-E* | 0.019 | Shared protein domains |
| *HLA-DPA1* | *HLA-DRB1* | 0.021 | Shared protein domains |
| *HLA-DPA1* | *HLA-DPB1* | 0.021 | Shared protein domains |
| *HLA-DPA1* | *HLA-DMA* | 0.042 | Shared protein domains |
| *HLA-DPA1* | *HLA-DQB1* | 0.021 | Shared protein domains |
| *HLA-DPA1* | *HLA-C* | 0.014 | Shared protein domains |
| *HLA-DPA1* | *HLA-F* | 0.015 | Shared protein domains |
| *HLA-DPA1* | *HLA-A* | 0.014 | Shared protein domains |
| *HLA-DPA1* | *HLA-DRA* | 0.078 | Shared protein domains |
| *HLA-DPA1* | *HLA-G* | 0.015 | Shared protein domains |
| *HLA-DPA1* | *HLA-B* | 0.014 | Shared protein domains |
| *HLA-DPA1* | *B2M* | 0.023 | Shared protein domains |
| *HLA-DPA1* | *HLA-E* | 0.014 | Shared protein domains |
| *HLA-DPA1* | *HLA-DRB1* | 0.017 | Shared protein domains |
| *HLA-DPA1* | *HLA-DPB1* | 0.017 | Shared protein domains |
| *HLA-DPA1* | *HLA-DMA* | 0.078 | Shared protein domains |
| *HLA-DPA1* | *HLA-DQB1* | 0.017 | Shared protein domains |
| *HLA-DPB1* | *HLA-DRA* | 0.011 | Co-expression |
| *HLA-DPB1* | *HLA-DRB1* | 0.010 | Co-expression |
| *HLA-DPB1* | *HLA-C* | 0.009 | Co-expression |
| *HLA-DPB1* | *HLA-F* | 0.007 | Co-expression |
| *HLA-DPB1* | *HLA-DRA* | 0.006 | Co-expression |
| *HLA-DPB1* | *HLA-B* | 0.008 | Co-expression |
| *HLA-DPB1* | *B2M* | 0.009 | Co-expression |
| *HLA-DPB1* | *HLA-E* | 0.006 | Co-expression |
| *HLA-DPB1* | *HLA-DRB1* | 0.005 | Co-expression |
| *HLA-DPB1* | *HLA-DRA* | 0.022 | Co-expression |
| *HLA-DPB1* | *HLA-DRB1* | 0.022 | Co-expression |
| *HLA-DPB1* | *HLA-C* | 0.006 | Co-expression |
| *HLA-DPB1* | *HLA-F* | 0.007 | Co-expression |
| *HLA-DPB1* | *HLA-A* | 0.007 | Co-expression |
| *HLA-DPB1* | *HLA-DRA* | 0.008 | Co-expression |
| *HLA-DPB1* | *B2M* | 0.005 | Co-expression |
| *HLA-DPB1* | *TAP1* | 0.006 | Co-expression |
| *HLA-DPB1* | *HLA-DRB1* | 0.008 | Co-expression |
| *HLA-DPB1* | *HLA-DRA* | 0.015 | Co-expression |
| *HLA-DPB1* | *HLA-F* | 0.028 | Co-expression |
| *HLA-DPB1* | *HLA-A* | 0.022 | Co-expression |
| *HLA-DPB1* | *HLA-DRA* | 0.024 | Co-expression |
| *HLA-DPB1* | *TAP1* | 0.022 | Co-expression |
| *HLA-DPB1* | *HLA-DRB1* | 0.026 | Co-expression |
| *HLA-DPB1* | *HLA-DRA* | 0.014 | Co-expression |
| *HLA-DPB1* | *HLA-DRB1* | 0.009 | Co-expression |
| *HLA-DPB1* | *HLA-C* | 0.017 | Co-expression |
| *HLA-DPB1* | *HLA-A* | 0.017 | Co-expression |
| *HLA-DPB1* | *HLA-DRA* | 0.029 | Co-expression |
| *HLA-DPB1* | *HLA-G* | 0.013 | Co-expression |
| *HLA-DPB1* | *HLA-B* | 0.018 | Co-expression |
| *HLA-DPB1* | *HLA-DRB1* | 0.028 | Co-expression |
| *HLA-DPB1* | *HLA-F* | 0.010 | Co-expression |
| *HLA-DPB1* | *AOAH* | 0.012 | Co-expression |
| *HLA-DPB1* | *HLA-A* | 0.011 | Co-expression |
| *HLA-DPB1* | *HLA-DRA* | 0.011 | Co-expression |
| *HLA-DPB1* | *HLA-G* | 0.009 | Co-expression |
| *HLA-DPB1* | *B2M* | 0.011 | Co-expression |
| *HLA-DPB1* | *HLA-E* | 0.007 | Co-expression |
| *HLA-DPB1* | *AOAH* | 0.019 | Co-expression |
| *HLA-DPB1* | *HLA-A* | 0.014 | Co-expression |
| *HLA-DPB1* | *HLA-DRA* | 0.018 | Co-expression |
| *HLA-DPB1* | *HLA-E* | 0.014 | Co-expression |
| *HLA-DPB1* | *HLA-C* | 0.009 | Co-expression |
| *HLA-DPB1* | *HLA-F* | 0.008 | Co-expression |
| *HLA-DPB1* | *HLA-A* | 0.008 | Co-expression |
| *HLA-DPB1* | *HLA-DRA* | 0.022 | Co-expression |
| *HLA-DPB1* | *HLA-B* | 0.009 | Co-expression |
| *HLA-DPB1* | *HLA-E* | 0.012 | Co-expression |
| *HLA-DPB1* | *TAP1* | 0.013 | Co-expression |
| *HLA-DPB1* | *HLA-DRB1* | 0.016 | Co-expression |
| *HLA-DPB1* | *HLA-A* | 0.004 | Co-expression |
| *HLA-DPB1* | *HLA-DRA* | 0.002 | Co-expression |
| *HLA-DPB1* | *HLA-DRB1* | 0.002 | Co-expression |
| *HLA-DPB1* | *HLA-DRA* | 0.022 | Co-expression |
| *HLA-DPB1* | *HLA-DRB1* | 0.026 | Co-expression |
| *HLA-DPB1* | *AOAH* | 0.010 | Co-expression |
| *HLA-DPB1* | *HLA-DRA* | 0.013 | Co-expression |
| *HLA-DPB1* | *HLA-DRA* | 0.009 | Co-expression |
| *HLA-DPB1* | *HLA-DRB1* | 0.009 | Co-expression |
| *HLA-DPB1* | *TBKBP1* | 0.016 | Co-expression |
| *HLA-DPB1* | *HLA-DRA* | 0.018 | Co-expression |
| *HLA-DPB1* | *AOAH* | 0.005 | Co-expression |
| *HLA-DPB1* | *HLA-A* | 0.003 | Co-expression |
| *HLA-DPB1* | *HLA-DRA* | 0.003 | Co-expression |
| *HLA-DPB1* | *HLA-G* | 0.005 | Co-expression |
| *HLA-DPB1* | *HLA-B* | 0.006 | Co-expression |
| *HLA-DPB1* | *B2M* | 0.007 | Co-expression |
| *HLA-DPB1* | *TAP1* | 0.006 | Co-expression |
| *HLA-DPB1* | *HLA-DRB1* | 0.005 | Co-expression |
| *HLA-DPB1* | *HLA-F* | 0.005 | Co-localization |
| *HLA-DPB1* | *HLA-DRA* | 0.007 | Co-localization |
| *HLA-DPB1* | *HLA-G* | 0.008 | Co-localization |
| *HLA-DPB1* | *B2M* | 0.006 | Co-localization |
| *HLA-DPB1* | *HLA-E* | 0.004 | Co-localization |
| *HLA-DPB1* | *TAP1* | 0.010 | Co-localization |
| *HLA-DPB1* | *TANK* | 0.011 | Co-localization |
| *HLA-DPB1* | *HLA-E* | 0.016 | Physical Interactions |
| *HLA-DPB1* | *HLA-C* | 0.066 | Predicted |
| *HLA-DPB1* | *HLA-F* | 0.119 | Predicted |
| *HLA-DPB1* | *HLA-G* | 0.091 | Predicted |
| *HLA-DPB1* | *HLA-B* | 0.049 | Predicted |
| *HLA-DPB1* | *HLA-DRB1* | 0.083 | Predicted |
| *HLA-DPB1* | *HLA-C* | 0.018 | Shared protein domains |
| *HLA-DPB1* | *HLA-F* | 0.019 | Shared protein domains |
| *HLA-DPB1* | *HLA-A* | 0.019 | Shared protein domains |
| *HLA-DPB1* | *HLA-DRA* | 0.021 | Shared protein domains |
| *HLA-DPB1* | *HLA-G* | 0.019 | Shared protein domains |
| *HLA-DPB1* | *HLA-B* | 0.019 | Shared protein domains |
| *HLA-DPB1* | *B2M* | 0.018 | Shared protein domains |
| *HLA-DPB1* | *HLA-E* | 0.019 | Shared protein domains |
| *HLA-DPB1* | *HLA-DRB1* | 0.031 | Shared protein domains |
| *HLA-DPB1* | *HLA-C* | 0.014 | Shared protein domains |
| *HLA-DPB1* | *HLA-F* | 0.015 | Shared protein domains |
| *HLA-DPB1* | *HLA-A* | 0.014 | Shared protein domains |
| *HLA-DPB1* | *HLA-DRA* | 0.017 | Shared protein domains |
| *HLA-DPB1* | *HLA-G* | 0.015 | Shared protein domains |
| *HLA-DPB1* | *HLA-B* | 0.014 | Shared protein domains |
| *HLA-DPB1* | *B2M* | 0.023 | Shared protein domains |
| *HLA-DPB1* | *HLA-E* | 0.014 | Shared protein domains |
| *HLA-DPB1* | *HLA-DRB1* | 0.047 | Shared protein domains |
| *HLA-DQB1* | *AOAH* | 0.013 | Co-expression |
| *HLA-DQB1* | *HLA-DRA* | 0.014 | Co-expression |
| *HLA-DQB1* | *HLA-DRB1* | 0.011 | Co-expression |
| *HLA-DQB1* | *HLA-DPB1* | 0.017 | Co-expression |
| *HLA-DQB1* | *HLA-DPB1* | 0.012 | Co-expression |
| *HLA-DQB1* | *HLA-DRA* | 0.015 | Co-expression |
| *HLA-DQB1* | *HLA-DMA* | 0.015 | Co-expression |
| *HLA-DQB1* | *HLA-DRB1* | 0.012 | Co-expression |
| *HLA-DQB1* | *HLA-C* | 0.006 | Co-expression |
| *HLA-DQB1* | *HLA-F* | 0.008 | Co-expression |
| *HLA-DQB1* | *HLA-A* | 0.006 | Co-expression |
| *HLA-DQB1* | *HLA-DRA* | 0.009 | Co-expression |
| *HLA-DQB1* | *B2M* | 0.005 | Co-expression |
| *HLA-DQB1* | *TAP1* | 0.006 | Co-expression |
| *HLA-DQB1* | *HLA-DRB1* | 0.009 | Co-expression |
| *HLA-DQB1* | *HLA-DPB1* | 0.005 | Co-expression |
| *HLA-DQB1* | *HLA-DMA* | 0.010 | Co-expression |
| *HLA-DQB1* | *HLA-DMA* | 0.010 | Co-expression |
| *HLA-DQB1* | *HLA-F* | 0.026 | Co-expression |
| *HLA-DQB1* | *HLA-A* | 0.020 | Co-expression |
| *HLA-DQB1* | *HLA-DRA* | 0.023 | Co-expression |
| *HLA-DQB1* | *TAP1* | 0.020 | Co-expression |
| *HLA-DQB1* | *HLA-DRB1* | 0.027 | Co-expression |
| *HLA-DQB1* | *HLA-DPB1* | 0.031 | Co-expression |
| *HLA-DQB1* | *HLA-DMA* | 0.024 | Co-expression |
| *HLA-DQB1* | *HLA-DRA* | 0.024 | Co-expression |
| *HLA-DQB1* | *HLA-DRB1* | 0.017 | Co-expression |
| *HLA-DQB1* | *HLA-DPB1* | 0.010 | Co-expression |
| *HLA-DQB1* | *HLA-DMA* | 0.017 | Co-expression |
| *HLA-DQB1* | *HLA-C* | 0.021 | Co-expression |
| *HLA-DQB1* | *HLA-A* | 0.022 | Co-expression |
| *HLA-DQB1* | *HLA-DRA* | 0.034 | Co-expression |
| *HLA-DQB1* | *HLA-G* | 0.019 | Co-expression |
| *HLA-DQB1* | *HLA-B* | 0.021 | Co-expression |
| *HLA-DQB1* | *HLA-DRB1* | 0.035 | Co-expression |
| *HLA-DQB1* | *HLA-DPB1* | 0.030 | Co-expression |
| *HLA-DQB1* | *HLA-DMA* | 0.022 | Co-expression |
| *HLA-DQB1* | *HLA-DRA* | 0.012 | Co-expression |
| *HLA-DQB1* | *HLA-G* | 0.010 | Co-expression |
| *HLA-DQB1* | *HLA-DPB1* | 0.016 | Co-expression |
| *HLA-DQB1* | *HLA-DMA* | 0.013 | Co-expression |
| *HLA-DQB1* | *HLA-DRA* | 0.016 | Co-expression |
| *HLA-DQB1* | *HLA-DMA* | 0.011 | Co-expression |
| *HLA-DQB1* | *HLA-F* | 0.008 | Co-expression |
| *HLA-DQB1* | *HLA-DRA* | 0.022 | Co-expression |
| *HLA-DQB1* | *HLA-E* | 0.012 | Co-expression |
| *HLA-DQB1* | *TAP1* | 0.012 | Co-expression |
| *HLA-DQB1* | *HLA-DRB1* | 0.016 | Co-expression |
| *HLA-DQB1* | *HLA-DPB1* | 0.034 | Co-expression |
| *HLA-DQB1* | *HLA-DMA* | 0.015 | Co-expression |
| *HLA-DQB1* | *HLA-C* | 0.004 | Co-expression |
| *HLA-DQB1* | *HLA-A* | 0.004 | Co-expression |
| *HLA-DQB1* | *HLA-DRA* | 0.002 | Co-expression |
| *HLA-DQB1* | *HLA-DRB1* | 0.002 | Co-expression |
| *HLA-DQB1* | *HLA-DPB1* | 0.002 | Co-expression |
| *HLA-DQB1* | *HLA-DMA* | 0.003 | Co-expression |
| *HLA-DQB1* | *HLA-C* | 0.020 | Co-expression |
| *HLA-DQB1* | *HLA-A* | 0.014 | Co-expression |
| *HLA-DQB1* | *HLA-DRA* | 0.026 | Co-expression |
| *HLA-DQB1* | *HLA-DRB1* | 0.034 | Co-expression |
| *HLA-DQB1* | *HLA-DPB1* | 0.029 | Co-expression |
| *HLA-DQB1* | *HLA-DMA* | 0.020 | Co-expression |
| *HLA-DQB1* | *HLA-DPB1* | 0.013 | Co-expression |
| *HLA-DQB1* | *HLA-DRA* | 0.014 | Co-expression |
| *HLA-DQB1* | *HLA-DRB1* | 0.015 | Co-expression |
| *HLA-DQB1* | *HLA-DPB1* | 0.014 | Co-expression |
| *HLA-DQB1* | *HLA-DMA* | 0.014 | Co-expression |
| *HLA-DQB1* | *HLA-A* | 0.003 | Co-expression |
| *HLA-DQB1* | *HLA-DRA* | 0.003 | Co-expression |
| *HLA-DQB1* | *HLA-G* | 0.005 | Co-expression |
| *HLA-DQB1* | *HLA-B* | 0.005 | Co-expression |
| *HLA-DQB1* | *B2M* | 0.007 | Co-expression |
| *HLA-DQB1* | *HLA-DRB1* | 0.005 | Co-expression |
| *HLA-DQB1* | *HLA-DPB1* | 0.003 | Co-expression |
| *HLA-DQB1* | *HLA-DMA* | 0.004 | Co-expression |
| *HLA-DQB1* | *HLA-C* | 0.018 | Shared protein domains |
| *HLA-DQB1* | *HLA-F* | 0.019 | Shared protein domains |
| *HLA-DQB1* | *HLA-A* | 0.019 | Shared protein domains |
| *HLA-DQB1* | *HLA-DRA* | 0.021 | Shared protein domains |
| *HLA-DQB1* | *HLA-G* | 0.019 | Shared protein domains |
| *HLA-DQB1* | *HLA-B* | 0.019 | Shared protein domains |
| *HLA-DQB1* | *B2M* | 0.018 | Shared protein domains |
| *HLA-DQB1* | *HLA-E* | 0.019 | Shared protein domains |
| *HLA-DQB1* | *HLA-DRB1* | 0.031 | Shared protein domains |
| *HLA-DQB1* | *HLA-DPB1* | 0.031 | Shared protein domains |
| *HLA-DQB1* | *HLA-DMA* | 0.021 | Shared protein domains |
| *HLA-DQB1* | *HLA-C* | 0.014 | Shared protein domains |
| *HLA-DQB1* | *HLA-F* | 0.015 | Shared protein domains |
| *HLA-DQB1* | *HLA-A* | 0.014 | Shared protein domains |
| *HLA-DQB1* | *HLA-DRA* | 0.017 | Shared protein domains |
| *HLA-DQB1* | *HLA-G* | 0.015 | Shared protein domains |
| *HLA-DQB1* | *HLA-B* | 0.014 | Shared protein domains |
| *HLA-DQB1* | *B2M* | 0.023 | Shared protein domains |
| *HLA-DQB1* | *HLA-E* | 0.014 | Shared protein domains |
| *HLA-DQB1* | *HLA-DRB1* | 0.047 | Shared protein domains |
| *HLA-DQB1* | *HLA-DPB1* | 0.047 | Shared protein domains |
| *HLA-DQB1* | *HLA-DMA* | 0.017 | Shared protein domains |
| *HLA-DRA* | *HLA-C* | 0.012 | Co-expression |
| *HLA-DRA* | *HLA-F* | 0.009 | Co-expression |
| *HLA-DRA* | *AOAH* | 0.007 | Co-expression |
| *HLA-DRA* | *HLA-C* | 0.011 | Co-expression |
| *HLA-DRA* | *HLA-F* | 0.007 | Co-expression |
| *HLA-DRA* | *AOAH* | 0.006 | Co-expression |
| *HLA-DRA* | *HLA-C* | 0.011 | Co-expression |
| *HLA-DRA* | *HLA-A* | 0.014 | Co-expression |
| *HLA-DRA* | *HLA-F* | 0.022 | Co-expression |
| *HLA-DRA* | *HLA-A* | 0.022 | Co-expression |
| *HLA-DRA* | *HLA-C* | 0.018 | Co-expression |
| *HLA-DRA* | *HLA-A* | 0.019 | Co-expression |
| *HLA-DRA* | *HLA-F* | 0.008 | Co-expression |
| *HLA-DRA* | *AOAH* | 0.009 | Co-expression |
| *HLA-DRA* | *HLA-A* | 0.008 | Co-expression |
| *HLA-DRA* | *AOAH* | 0.014 | Co-expression |
| *HLA-DRA* | *HLA-A* | 0.014 | Co-expression |
| *HLA-DRA* | *LRRK2* | 0.014 | Co-expression |
| *HLA-DRA* | *HLA-C* | 0.007 | Co-expression |
| *HLA-DRA* | *HLA-F* | 0.005 | Co-expression |
| *HLA-DRA* | *HLA-A* | 0.005 | Co-expression |
| *HLA-DRA* | *HLA-A* | 0.025 | Co-expression |
| *HLA-DRA* | *HLA-C* | 0.004 | Co-expression |
| *HLA-DRA* | *AOAH* | 0.004 | Co-expression |
| *HLA-DRA* | *HLA-A* | 0.004 | Co-expression |
| *HLA-DRA* | *HLA-F* | 0.006 | Co-expression |
| *HLA-DRA* | *HLA-A* | 0.003 | Co-expression |
| *HLA-DRA* | *HLA-F* | 0.004 | Co-localization |
| *HLA-DRA* | *HLA-C* | 0.018 | Shared protein domains |
| *HLA-DRA* | *HLA-F* | 0.019 | Shared protein domains |
| *HLA-DRA* | *HLA-A* | 0.019 | Shared protein domains |
| *HLA-DRA* | *HLA-C* | 0.014 | Shared protein domains |
| *HLA-DRA* | *HLA-F* | 0.015 | Shared protein domains |
| *HLA-DRA* | *HLA-A* | 0.014 | Shared protein domains |
| *HLA-DRB1* | *HLA-C* | 0.008 | Co-expression |
| *HLA-DRB1* | *HLA-F* | 0.008 | Co-expression |
| *HLA-DRB1* | *HLA-A* | 0.008 | Co-expression |
| *HLA-DRB1* | *HLA-DRA* | 0.007 | Co-expression |
| *HLA-DRB1* | *HLA-B* | 0.009 | Co-expression |
| *HLA-DRB1* | *HLA-C* | 0.009 | Co-expression |
| *HLA-DRB1* | *HLA-F* | 0.006 | Co-expression |
| *HLA-DRB1* | *AOAH* | 0.005 | Co-expression |
| *HLA-DRB1* | *HLA-A* | 0.007 | Co-expression |
| *HLA-DRB1* | *HLA-DRA* | 0.005 | Co-expression |
| *HLA-DRB1* | *HLA-G* | 0.007 | Co-expression |
| *HLA-DRB1* | *HLA-B* | 0.008 | Co-expression |
| *HLA-DRB1* | *B2M* | 0.007 | Co-expression |
| *HLA-DRB1* | *HLA-E* | 0.005 | Co-expression |
| *HLA-DRB1* | *HLA-DRA* | 0.019 | Co-expression |
| *HLA-DRB1* | *HLA-DRA* | 0.022 | Co-expression |
| *HLA-DRB1* | *HLA-C* | 0.009 | Co-expression |
| *HLA-DRB1* | *HLA-F* | 0.014 | Co-expression |
| *HLA-DRB1* | *HLA-DRA* | 0.015 | Co-expression |
| *HLA-DRB1* | *B2M* | 0.008 | Co-expression |
| *HLA-DRB1* | *TAP1* | 0.011 | Co-expression |
| *HLA-DRB1* | *HLA-F* | 0.023 | Co-expression |
| *HLA-DRB1* | *HLA-A* | 0.023 | Co-expression |
| *HLA-DRB1* | *HLA-DRA* | 0.024 | Co-expression |
| *HLA-DRB1* | *B2M* | 0.016 | Co-expression |
| *HLA-DRB1* | *TAP1* | 0.019 | Co-expression |
| *HLA-DRB1* | *HLA-F* | 0.010 | Co-expression |
| *HLA-DRB1* | *HLA-A* | 0.011 | Co-expression |
| *HLA-DRB1* | *HLA-DRA* | 0.019 | Co-expression |
| *HLA-DRB1* | *B2M* | 0.012 | Co-expression |
| *HLA-DRB1* | *TAP1* | 0.007 | Co-expression |
| *HLA-DRB1* | *HLA-C* | 0.020 | Co-expression |
| *HLA-DRB1* | *HLA-A* | 0.020 | Co-expression |
| *HLA-DRB1* | *HLA-DRA* | 0.031 | Co-expression |
| *HLA-DRB1* | *HLA-G* | 0.015 | Co-expression |
| *HLA-DRB1* | *HLA-B* | 0.021 | Co-expression |
| *HLA-DRB1* | *LRRK2* | 0.011 | Co-expression |
| *HLA-DRB1* | *HLA-C* | 0.005 | Co-expression |
| *HLA-DRB1* | *HLA-F* | 0.004 | Co-expression |
| *HLA-DRB1* | *HLA-A* | 0.004 | Co-expression |
| *HLA-DRB1* | *HLA-DRA* | 0.012 | Co-expression |
| *HLA-DRB1* | *HLA-G* | 0.007 | Co-expression |
| *HLA-DRB1* | *HLA-B* | 0.005 | Co-expression |
| *HLA-DRB1* | *B2M* | 0.012 | Co-expression |
| *HLA-DRB1* | *HLA-E* | 0.007 | Co-expression |
| *HLA-DRB1* | *TAP1* | 0.007 | Co-expression |
| *HLA-DRB1* | *HLA-C* | 0.004 | Co-expression |
| *HLA-DRB1* | *AOAH* | 0.004 | Co-expression |
| *HLA-DRB1* | *HLA-A* | 0.004 | Co-expression |
| *HLA-DRB1* | *HLA-DRA* | 0.002 | Co-expression |
| *HLA-DRB1* | *HLA-DRA* | 0.024 | Co-expression |
| *HLA-DRB1* | *LRRK2* | 0.007 | Co-expression |
| *HLA-DRB1* | *HLA-F* | 0.007 | Co-expression |
| *HLA-DRB1* | *HLA-DRA* | 0.009 | Co-expression |
| *HLA-DRB1* | *HLA-B* | 0.007 | Co-expression |
| *HLA-DRB1* | *HLA-E* | 0.007 | Co-expression |
| *HLA-DRB1* | *HLA-A* | 0.004 | Co-expression |
| *HLA-DRB1* | *HLA-DRA* | 0.006 | Co-expression |
| *HLA-DRB1* | *HLA-B* | 0.011 | Co-expression |
| *HLA-DRB1* | *B2M* | 0.012 | Co-expression |
| *HLA-DRB1* | *HLA-DRA* | 0.045 | Physical Interactions |
| *HLA-DRB1* | *HLA-C* | 0.021 | Predicted |
| *HLA-DRB1* | *HLA-F* | 0.037 | Predicted |
| *HLA-DRB1* | *HLA-A* | 0.021 | Predicted |
| *HLA-DRB1* | *HLA-DRA* | 0.038 | Predicted |
| *HLA-DRB1* | *HLA-G* | 0.028 | Predicted |
| *HLA-DRB1* | *HLA-B* | 0.015 | Predicted |
| *HLA-DRB1* | *B2M* | 0.034 | Predicted |
| *HLA-DRB1* | *HLA-E* | 0.028 | Predicted |
| *HLA-DRB1* | *HLA-C* | 0.018 | Shared protein domains |
| *HLA-DRB1* | *HLA-F* | 0.019 | Shared protein domains |
| *HLA-DRB1* | *HLA-A* | 0.019 | Shared protein domains |
| *HLA-DRB1* | *HLA-DRA* | 0.021 | Shared protein domains |
| *HLA-DRB1* | *HLA-G* | 0.019 | Shared protein domains |
| *HLA-DRB1* | *HLA-B* | 0.019 | Shared protein domains |
| *HLA-DRB1* | *B2M* | 0.018 | Shared protein domains |
| *HLA-DRB1* | *HLA-E* | 0.019 | Shared protein domains |
| *HLA-DRB1* | *HLA-C* | 0.014 | Shared protein domains |
| *HLA-DRB1* | *HLA-F* | 0.015 | Shared protein domains |
| *HLA-DRB1* | *HLA-A* | 0.014 | Shared protein domains |
| *HLA-DRB1* | *HLA-DRA* | 0.017 | Shared protein domains |
| *HLA-DRB1* | *HLA-G* | 0.015 | Shared protein domains |
| *HLA-DRB1* | *HLA-B* | 0.014 | Shared protein domains |
| *HLA-DRB1* | *B2M* | 0.023 | Shared protein domains |
| *HLA-DRB1* | *HLA-E* | 0.014 | Shared protein domains |
| *HLA-DRB5* | *HLA-F* | 0.016 | Co-expression |
| *HLA-DRB5* | *AOAH* | 0.011 | Co-expression |
| *HLA-DRB5* | *HLA-DRA* | 0.013 | Co-expression |
| *HLA-DRB5* | *HLA-G* | 0.016 | Co-expression |
| *HLA-DRB5* | *HLA-DRB1* | 0.012 | Co-expression |
| *HLA-DRB5* | *HLA-DPB1* | 0.017 | Co-expression |
| *HLA-DRB5* | *HLA-DMA* | 0.012 | Co-expression |
| *HLA-DRB5* | *HLA-DQB1* | 0.027 | Co-expression |
| *HLA-DRB5* | *CD74* | 0.011 | Co-expression |
| *HLA-DRB5* | *HLA-DPA1* | 0.010 | Co-expression |
| *HLA-DRB5* | *HLA-C* | 0.007 | Co-expression |
| *HLA-DRB5* | *HLA-F* | 0.007 | Co-expression |
| *HLA-DRB5* | *HLA-A* | 0.007 | Co-expression |
| *HLA-DRB5* | *HLA-DRA* | 0.009 | Co-expression |
| *HLA-DRB5* | *B2M* | 0.006 | Co-expression |
| *HLA-DRB5* | *TAP1* | 0.007 | Co-expression |
| *HLA-DRB5* | *HLA-DRB1* | 0.009 | Co-expression |
| *HLA-DRB5* | *HLA-DPB1* | 0.006 | Co-expression |
| *HLA-DRB5* | *HLA-DMA* | 0.009 | Co-expression |
| *HLA-DRB5* | *HLA-DQB1* | 0.006 | Co-expression |
| *HLA-DRB5* | *HLA-DPA1* | 0.006 | Co-expression |
| *HLA-DRB5* | *HLA-G* | 0.019 | Co-expression |
| *HLA-DRB5* | *HLA-DRB1* | 0.013 | Co-expression |
| *HLA-DRB5* | *HLA-DQB1* | 0.021 | Co-expression |
| *HLA-DRB5* | *HLA-DRA* | 0.030 | Co-expression |
| *HLA-DRB5* | *HLA-DRB1* | 0.021 | Co-expression |
| *HLA-DRB5* | *HLA-DPB1* | 0.013 | Co-expression |
| *HLA-DRB5* | *HLA-DMA* | 0.017 | Co-expression |
| *HLA-DRB5* | *HLA-DQB1* | 0.027 | Co-expression |
| *HLA-DRB5* | *CD74* | 0.023 | Co-expression |
| *HLA-DRB5* | *HLA-DPA1* | 0.013 | Co-expression |
| *HLA-DRB5* | *HLA-DRA* | 0.004 | Co-expression |
| *HLA-DRB5* | *B2M* | 0.009 | Co-expression |
| *HLA-DRB5* | *HLA-DRB1* | 0.004 | Co-expression |
| *HLA-DRB5* | *HLA-DPB1* | 0.004 | Co-expression |
| *HLA-DRB5* | *HLA-DMA* | 0.007 | Co-expression |
| *HLA-DRB5* | *HLA-DQB1* | 0.004 | Co-expression |
| *HLA-DRB5* | *HLA-DPA1* | 0.004 | Co-expression |
| *HLA-DRB5* | *HLA-F* | 0.007 | Co-expression |
| *HLA-DRB5* | *HLA-DRA* | 0.010 | Co-expression |
| *HLA-DRB5* | *HLA-E* | 0.008 | Co-expression |
| *HLA-DRB5* | *HLA-DRB1* | 0.011 | Co-expression |
| *HLA-DRB5* | *HLA-DPB1* | 0.010 | Co-expression |
| *HLA-DRB5* | *HLA-DMA* | 0.010 | Co-expression |
| *HLA-DRB5* | *HLA-DQB1* | 0.016 | Co-expression |
| *HLA-DRB5* | *CD74* | 0.010 | Co-expression |
| *HLA-DRB5* | *HLA-DPA1* | 0.008 | Co-expression |
| *HLA-DRB5* | *HLA-A* | 0.128 | Physical Interactions |
| *HLA-DRB5* | *HLA-DRA* | 0.067 | Physical Interactions |
| *HLA-DRB5* | *HLA-DRB1* | 0.176 | Physical Interactions |
| *HLA-DRB5* | *HLA-C* | 0.018 | Shared protein domains |
| *HLA-DRB5* | *HLA-F* | 0.019 | Shared protein domains |
| *HLA-DRB5* | *HLA-A* | 0.019 | Shared protein domains |
| *HLA-DRB5* | *HLA-DRA* | 0.021 | Shared protein domains |
| *HLA-DRB5* | *HLA-G* | 0.019 | Shared protein domains |
| *HLA-DRB5* | *HLA-B* | 0.019 | Shared protein domains |
| *HLA-DRB5* | *B2M* | 0.018 | Shared protein domains |
| *HLA-DRB5* | *HLA-E* | 0.019 | Shared protein domains |
| *HLA-DRB5* | *HLA-DRB1* | 0.031 | Shared protein domains |
| *HLA-DRB5* | *HLA-DPB1* | 0.031 | Shared protein domains |
| *HLA-DRB5* | *HLA-DMA* | 0.021 | Shared protein domains |
| *HLA-DRB5* | *HLA-DQB1* | 0.031 | Shared protein domains |
| *HLA-DRB5* | *HLA-DPA1* | 0.021 | Shared protein domains |
| *HLA-DRB5* | *HLA-C* | 0.014 | Shared protein domains |
| *HLA-DRB5* | *HLA-F* | 0.015 | Shared protein domains |
| *HLA-DRB5* | *HLA-A* | 0.014 | Shared protein domains |
| *HLA-DRB5* | *HLA-DRA* | 0.017 | Shared protein domains |
| *HLA-DRB5* | *HLA-G* | 0.015 | Shared protein domains |
| *HLA-DRB5* | *HLA-B* | 0.014 | Shared protein domains |
| *HLA-DRB5* | *B2M* | 0.023 | Shared protein domains |
| *HLA-DRB5* | *HLA-E* | 0.014 | Shared protein domains |
| *HLA-DRB5* | *HLA-DRB1* | 0.047 | Shared protein domains |
| *HLA-DRB5* | *HLA-DPB1* | 0.047 | Shared protein domains |
| *HLA-DRB5* | *HLA-DMA* | 0.017 | Shared protein domains |
| *HLA-DRB5* | *HLA-DQB1* | 0.047 | Shared protein domains |
| *HLA-DRB5* | *HLA-DPA1* | 0.017 | Shared protein domains |
| *HLA-E* | *HLA-C* | 0.026 | Co-expression |
| *HLA-E* | *HLA-F* | 0.026 | Co-expression |
| *HLA-E* | *HLA-A* | 0.030 | Co-expression |
| *HLA-E* | *HLA-G* | 0.026 | Co-expression |
| *HLA-E* | *HLA-B* | 0.028 | Co-expression |
| *HLA-E* | *HLA-C* | 0.013 | Co-expression |
| *HLA-E* | *HLA-F* | 0.009 | Co-expression |
| *HLA-E* | *HLA-A* | 0.012 | Co-expression |
| *HLA-E* | *HLA-DRA* | 0.006 | Co-expression |
| *HLA-E* | *HLA-G* | 0.010 | Co-expression |
| *HLA-E* | *HLA-B* | 0.012 | Co-expression |
| *HLA-E* | *B2M* | 0.010 | Co-expression |
| *HLA-E* | *HLA-G* | 0.032 | Co-expression |
| *HLA-E* | *HLA-C* | 0.020 | Co-expression |
| *HLA-E* | *HLA-F* | 0.028 | Co-expression |
| *HLA-E* | *HLA-C* | 0.029 | Co-expression |
| *HLA-E* | *HLA-F* | 0.026 | Co-expression |
| *HLA-E* | *HLA-A* | 0.031 | Co-expression |
| *HLA-E* | *HLA-G* | 0.028 | Co-expression |
| *HLA-E* | *HLA-B* | 0.029 | Co-expression |
| *HLA-E* | *B2M* | 0.024 | Co-expression |
| *HLA-E* | *HLA-F* | 0.007 | Co-expression |
| *HLA-E* | *AOAH* | 0.007 | Co-expression |
| *HLA-E* | *HLA-A* | 0.007 | Co-expression |
| *HLA-E* | *HLA-DRA* | 0.006 | Co-expression |
| *HLA-E* | *HLA-G* | 0.005 | Co-expression |
| *HLA-E* | *HLA-F* | 0.013 | Co-expression |
| *HLA-E* | *HLA-DRA* | 0.009 | Co-expression |
| *HLA-E* | *HLA-G* | 0.012 | Co-expression |
| *HLA-E* | *B2M* | 0.010 | Co-expression |
| *HLA-E* | *HLA-C* | 0.004 | Co-expression |
| *HLA-E* | *HLA-F* | 0.004 | Co-expression |
| *HLA-E* | *HLA-A* | 0.004 | Co-expression |
| *HLA-E* | *HLA-DRA* | 0.009 | Co-expression |
| *HLA-E* | *HLA-G* | 0.006 | Co-expression |
| *HLA-E* | *HLA-B* | 0.004 | Co-expression |
| *HLA-E* | *B2M* | 0.010 | Co-expression |
| *HLA-E* | *HLA-F* | 0.035 | Co-expression |
| *HLA-E* | *HLA-C* | 0.008 | Co-expression |
| *HLA-E* | *HLA-F* | 0.006 | Co-expression |
| *HLA-E* | *HLA-A* | 0.007 | Co-expression |
| *HLA-E* | *HLA-DRA* | 0.006 | Co-expression |
| *HLA-E* | *HLA-G* | 0.006 | Co-expression |
| *HLA-E* | *HLA-B* | 0.006 | Co-expression |
| *HLA-E* | *B2M* | 0.011 | Co-expression |
| *HLA-E* | *HLA-G* | 0.013 | Co-expression |
| *HLA-E* | *HLA-F* | 0.005 | Co-localization |
| *HLA-E* | *HLA-DRA* | 0.004 | Co-localization |
| *HLA-E* | *HLA-G* | 0.007 | Co-localization |
| *HLA-E* | *B2M* | 0.005 | Co-localization |
| *HLA-E* | *HLA-C* | 0.011 | Physical Interactions |
| *HLA-E* | *HLA-F* | 0.035 | Physical Interactions |
| *HLA-E* | *HLA-A* | 0.025 | Physical Interactions |
| *HLA-E* | *HLA-B* | 0.011 | Physical Interactions |
| *HLA-E* | *HLA-C* | 0.022 | Predicted |
| *HLA-E* | *HLA-F* | 0.040 | Predicted |
| *HLA-E* | *HLA-A* | 0.023 | Predicted |
| *HLA-E* | *HLA-DRA* | 0.041 | Predicted |
| *HLA-E* | *HLA-G* | 0.030 | Predicted |
| *HLA-E* | *HLA-B* | 0.016 | Predicted |
| *HLA-E* | *HLA-C* | 0.044 | Shared protein domains |
| *HLA-E* | *HLA-F* | 0.034 | Shared protein domains |
| *HLA-E* | *HLA-A* | 0.044 | Shared protein domains |
| *HLA-E* | *HLA-DRA* | 0.019 | Shared protein domains |
| *HLA-E* | *HLA-G* | 0.034 | Shared protein domains |
| *HLA-E* | *HLA-B* | 0.044 | Shared protein domains |
| *HLA-E* | *B2M* | 0.017 | Shared protein domains |
| *HLA-E* | *HLA-C* | 0.055 | Shared protein domains |
| *HLA-E* | *HLA-F* | 0.032 | Shared protein domains |
| *HLA-E* | *HLA-A* | 0.055 | Shared protein domains |
| *HLA-E* | *HLA-DRA* | 0.014 | Shared protein domains |
| *HLA-E* | *HLA-G* | 0.032 | Shared protein domains |
| *HLA-E* | *HLA-B* | 0.055 | Shared protein domains |
| *HLA-E* | *B2M* | 0.019 | Shared protein domains |
| *HLA-F* | *HLA-C* | 0.033 | Co-expression |
| *HLA-F* | *HLA-C* | 0.019 | Co-expression |
| *HLA-F* | *HLA-C* | 0.031 | Co-expression |
| *HLA-F* | *HLA-C* | 0.030 | Co-expression |
| *HLA-F* | *HLA-C* | 0.016 | Co-expression |
| *HLA-F* | *HLA-C* | 0.030 | Co-expression |
| *HLA-F* | *HLA-C* | 0.026 | Co-expression |
| *HLA-F* | *HLA-C* | 0.003 | Co-expression |
| *HLA-F* | *HLA-C* | 0.007 | Co-expression |
| *HLA-F* | *HLA-C* | 0.039 | Physical Interactions |
| *HLA-F* | *HLA-C* | 0.030 | Predicted |
| *HLA-F* | *HLA-C* | 0.034 | Shared protein domains |
| *HLA-F* | *HLA-C* | 0.032 | Shared protein domains |
| *HLA-G* | *HLA-C* | 0.050 | Co-expression |
| *HLA-G* | *HLA-F* | 0.033 | Co-expression |
| *HLA-G* | *AOAH* | 0.011 | Co-expression |
| *HLA-G* | *HLA-A* | 0.044 | Co-expression |
| *HLA-G* | *HLA-DRA* | 0.011 | Co-expression |
| *HLA-G* | *HLA-C* | 0.027 | Co-expression |
| *HLA-G* | *HLA-F* | 0.016 | Co-expression |
| *HLA-G* | *HLA-A* | 0.024 | Co-expression |
| *HLA-G* | *HLA-DRA* | 0.009 | Co-expression |
| *HLA-G* | *HLA-C* | 0.035 | Co-expression |
| *HLA-G* | *HLA-F* | 0.030 | Co-expression |
| *HLA-G* | *HLA-A* | 0.034 | Co-expression |
| *HLA-G* | *HLA-C* | 0.036 | Co-expression |
| *HLA-G* | *HLA-F* | 0.031 | Co-expression |
| *HLA-G* | *HLA-A* | 0.036 | Co-expression |
| *HLA-G* | *HLA-F* | 0.025 | Co-expression |
| *HLA-G* | *HLA-DRA* | 0.009 | Co-expression |
| *HLA-G* | *HLA-C* | 0.034 | Co-expression |
| *HLA-G* | *HLA-F* | 0.027 | Co-expression |
| *HLA-G* | *HLA-A* | 0.029 | Co-expression |
| *HLA-G* | *HLA-C* | 0.020 | Co-expression |
| *HLA-G* | *HLA-A* | 0.022 | Co-expression |
| *HLA-G* | *HLA-DRA* | 0.016 | Co-expression |
| *HLA-G* | *HLA-F* | 0.008 | Co-expression |
| *HLA-G* | *AOAH* | 0.008 | Co-expression |
| *HLA-G* | *HLA-A* | 0.009 | Co-expression |
| *HLA-G* | *HLA-DRA* | 0.007 | Co-expression |
| *HLA-G* | *HLA-F* | 0.020 | Co-expression |
| *HLA-G* | *HLA-A* | 0.017 | Co-expression |
| *HLA-G* | *HLA-DRA* | 0.013 | Co-expression |
| *HLA-G* | *HLA-C* | 0.005 | Co-expression |
| *HLA-G* | *HLA-F* | 0.004 | Co-expression |
| *HLA-G* | *HLA-A* | 0.004 | Co-expression |
| *HLA-G* | *HLA-DRA* | 0.009 | Co-expression |
| *HLA-G* | *HLA-A* | 0.033 | Co-expression |
| *HLA-G* | *HLA-DRA* | 0.031 | Co-expression |
| *HLA-G* | *HLA-A* | 0.020 | Co-expression |
| *HLA-G* | *HLA-F* | 0.040 | Co-expression |
| *HLA-G* | *HLA-A* | 0.021 | Co-expression |
| *HLA-G* | *HLA-C* | 0.008 | Co-expression |
| *HLA-G* | *HLA-F* | 0.006 | Co-expression |
| *HLA-G* | *HLA-A* | 0.007 | Co-expression |
| *HLA-G* | *HLA-F* | 0.027 | Co-expression |
| *HLA-G* | *HLA-A* | 0.027 | Co-expression |
| *HLA-G* | *HLA-A* | 0.006 | Co-expression |
| *HLA-G* | *HLA-DRA* | 0.006 | Co-expression |
| *HLA-G* | *HLA-F* | 0.009 | Co-localization |
| *HLA-G* | *HLA-DRA* | 0.006 | Co-localization |
| *HLA-G* | *HLA-C* | 0.267 | Physical Interactions |
| *HLA-G* | *HLA-C* | 0.023 | Predicted |
| *HLA-G* | *HLA-F* | 0.041 | Predicted |
| *HLA-G* | *HLA-A* | 0.023 | Predicted |
| *HLA-G* | *HLA-C* | 0.034 | Shared protein domains |
| *HLA-G* | *HLA-F* | 0.035 | Shared protein domains |
| *HLA-G* | *HLA-A* | 0.034 | Shared protein domains |
| *HLA-G* | *HLA-DRA* | 0.019 | Shared protein domains |
| *HLA-G* | *HLA-C* | 0.032 | Shared protein domains |
| *HLA-G* | *HLA-F* | 0.034 | Shared protein domains |
| *HLA-G* | *HLA-A* | 0.032 | Shared protein domains |
| *HLA-G* | *HLA-DRA* | 0.015 | Shared protein domains |
| *MAP2* | *PGBD5* | 0.004 | Co-expression |
| *MAP2* | *AOAH* | 0.009 | Co-expression |
| *MAP2* | *PGBD5* | 0.005 | Co-localization |
| *MAP2* | *MAPT* | 0.140 | Predicted |
| *MAP2* | *MAPT* | 0.309 | Shared protein domains |
| *MAP2* | *MAP4* | 0.309 | Shared protein domains |
| *MAP2* | *MAPT* | 0.305 | Shared protein domains |
| *MAP2* | *MAP4* | 0.305 | Shared protein domains |
| *MAP4* | *MAPT* | 0.634 | Shared protein domains |
| *MAP4* | *MAPT* | 0.634 | Shared protein domains |
| *MAPT* | *PGBD5* | 0.005 | Co-expression |
| *MAPT* | *PGBD5* | 0.008 | Co-localization |
| *PGBD2* | *PGBD5* | 0.283 | Shared protein domains |
| *PGBD2* | *PGBD3* | 0.283 | Shared protein domains |
| *PGBD2* | *PGBD4* | 0.283 | Shared protein domains |
| *PGBD2* | *PGBD5* | 0.272 | Shared protein domains |
| *PGBD2* | *PGBD3* | 0.272 | Shared protein domains |
| *PGBD2* | *PGBD4* | 0.272 | Shared protein domains |
| *PGBD3* | *PGBD5* | 0.283 | Shared protein domains |
| *PGBD3* | *PGBD5* | 0.272 | Shared protein domains |
| *PGBD4* | *PGBD5* | 0.283 | Shared protein domains |
| *PGBD4* | *PGBD3* | 0.283 | Shared protein domains |
| *PGBD4* | *PGBD5* | 0.272 | Shared protein domains |
| *PGBD4* | *PGBD3* | 0.272 | Shared protein domains |
| *TANK* | *HLA-B* | 0.008 | Co-expression |
| *TANK* | *TAP1* | 0.007 | Co-expression |
| *TANK* | *HLA-DRA* | 0.009 | Co-localization |
| *TANK* | *TBKBP1* | 0.500 | Shared protein domains |
| *TANK* | *AZI2* | 0.500 | Shared protein domains |
| *TANK* | *TBKBP1* | 0.500 | Shared protein domains |
| *TANK* | *AZI2* | 0.500 | Shared protein domains |
| *TAP1* | *HLA-C* | 0.019 | Co-expression |
| *TAP1* | *HLA-F* | 0.018 | Co-expression |
| *TAP1* | *HLA-G* | 0.019 | Co-expression |
| *TAP1* | *HLA-C* | 0.020 | Co-expression |
| *TAP1* | *HLA-F* | 0.014 | Co-expression |
| *TAP1* | *AOAH* | 0.009 | Co-expression |
| *TAP1* | *HLA-A* | 0.017 | Co-expression |
| *TAP1* | *HLA-G* | 0.017 | Co-expression |
| *TAP1* | *HLA-B* | 0.019 | Co-expression |
| *TAP1* | *HLA-E* | 0.009 | Co-expression |
| *TAP1* | *HLA-C* | 0.022 | Co-expression |
| *TAP1* | *HLA-F* | 0.023 | Co-expression |
| *TAP1* | *HLA-A* | 0.020 | Co-expression |
| *TAP1* | *HLA-F* | 0.016 | Co-expression |
| *TAP1* | *HLA-G* | 0.014 | Co-expression |
| *TAP1* | *HLA-C* | 0.018 | Co-expression |
| *TAP1* | *HLA-F* | 0.019 | Co-expression |
| *TAP1* | *HLA-A* | 0.021 | Co-expression |
| *TAP1* | *HLA-DRA* | 0.014 | Co-expression |
| *TAP1* | *B2M* | 0.009 | Co-expression |
| *TAP1* | *HLA-F* | 0.013 | Co-expression |
| *TAP1* | *HLA-F* | 0.029 | Co-expression |
| *TAP1* | *HLA-A* | 0.021 | Co-expression |
| *TAP1* | *HLA-DRA* | 0.017 | Co-expression |
| *TAP1* | *HLA-C* | 0.011 | Co-expression |
| *TAP1* | *HLA-F* | 0.012 | Co-expression |
| *TAP1* | *HLA-A* | 0.013 | Co-expression |
| *TAP1* | *HLA-G* | 0.014 | Co-expression |
| *TAP1* | *HLA-B* | 0.012 | Co-expression |
| *TAP1* | *B2M* | 0.013 | Co-expression |
| *TAP1* | *HLA-E* | 0.012 | Co-expression |
| *TAP1* | *HLA-F* | 0.011 | Co-expression |
| *TAP1* | *HLA-A* | 0.012 | Co-expression |
| *TAP1* | *HLA-DRA* | 0.008 | Co-expression |
| *TAP1* | *HLA-G* | 0.009 | Co-expression |
| *TAP1* | *B2M* | 0.011 | Co-expression |
| *TAP1* | *HLA-E* | 0.007 | Co-expression |
| *TAP1* | *HLA-F* | 0.011 | Co-expression |
| *TAP1* | *HLA-A* | 0.008 | Co-expression |
| *TAP1* | *HLA-G* | 0.012 | Co-expression |
| *TAP1* | *B2M* | 0.011 | Co-expression |
| *TAP1* | *HLA-C* | 0.005 | Co-expression |
| *TAP1* | *HLA-F* | 0.004 | Co-expression |
| *TAP1* | *HLA-A* | 0.004 | Co-expression |
| *TAP1* | *HLA-DRA* | 0.008 | Co-expression |
| *TAP1* | *HLA-G* | 0.006 | Co-expression |
| *TAP1* | *HLA-B* | 0.005 | Co-expression |
| *TAP1* | *B2M* | 0.010 | Co-expression |
| *TAP1* | *HLA-E* | 0.006 | Co-expression |
| *TAP1* | *HLA-C* | 0.010 | Co-expression |
| *TAP1* | *HLA-A* | 0.012 | Co-expression |
| *TAP1* | *B2M* | 0.012 | Co-expression |
| *TAP1* | *HLA-A* | 0.017 | Co-expression |
| *TAP1* | *HLA-F* | 0.023 | Co-expression |
| *TAP1* | *HLA-G* | 0.019 | Co-expression |
| *TAP1* | *HLA-C* | 0.009 | Co-expression |
| *TAP1* | *HLA-F* | 0.007 | Co-expression |
| *TAP1* | *HLA-A* | 0.008 | Co-expression |
| *TAP1* | *HLA-G* | 0.007 | Co-expression |
| *TAP1* | *HLA-B* | 0.007 | Co-expression |
| *TAP1* | *HLA-E* | 0.007 | Co-expression |
| *TAP1* | *HLA-F* | 0.019 | Co-expression |
| *TAP1* | *HLA-A* | 0.017 | Co-expression |
| *TAP1* | *HLA-G* | 0.018 | Co-expression |
| *TAP1* | *HLA-A* | 0.007 | Co-expression |
| *TAP1* | *HLA-DRA* | 0.008 | Co-expression |
| *TAP1* | *B2M* | 0.026 | Co-expression |
| *TAP1* | *HLA-F* | 0.008 | Co-localization |
| *TAP1* | *HLA-DRA* | 0.009 | Co-localization |
| *TAP1* | *HLA-E* | 0.007 | Co-localization |
| *TAP1* | *HLA-F* | 0.069 | Predicted |
| *TAP1* | *HLA-A* | 0.039 | Predicted |
| *TAP1* | *HLA-G* | 0.052 | Predicted |
| *TAP1* | *HLA-B* | 0.028 | Predicted |
| *TAP1* | *HLA-E* | 0.052 | Predicted |
